# A low-cost, open-source device to evaluate limb stiffness in a rabbit model of cerebral palsy

**DOI:** 10.3389/fbioe.2025.1554775

**Published:** 2025-06-05

**Authors:** Preston R. Steele, Joel Feldmann, Katharina A. Quinlan, Marin Manuel

**Affiliations:** ^1^ Department of Biomedical and Pharmaceutical Sciences, College of Pharmacy, University of Rhode Island, Kingston, RI, United States; ^2^ George and Anne Ryan Institute for Neuroscience, University of Rhode Island, Kingston, RI, United States

**Keywords:** cerebral palsy, joint stiffness, torquemeter, spasticity, dystonia, preclinical research, open-source device, arduino

## Abstract

**Background:**

Movement disorders such as cerebral palsy (CP) are frequently associated with joint and muscle stiffness, often evaluated using subjective clinical methods like the Modified Ashworth Scale or Tardieu Scale. These approaches lack precision and reproducibility, particularly in preclinical models, limiting their utility in translational research.

**Methods:**

This study presents the development of a low-cost, open-source torquemeter device tailored for use in a neonatal rabbit model of CP. The device is designed to quantify joint stiffness objectively by measuring torque across a range of controlled joint rotation speeds, a key factor in evaluating hypertonia associated with spasticity and dystonia. The construction process is straightforward, with all components being either commercially available or 3D-printable and requiring only basic assembly tools.

**Results:**

The torquemeter demonstrated precise, reproducible measurements of torque and joint stiffness in pilot studies, validating its applicability in preclinical settings. By eliminating subjective biases, the device provides robust data to assess the effectiveness of therapeutic interventions targeting spasticity.

**Conclusion:**

This low-cost torquemeter offers an accessible, reliable tool for preclinical movement disorder research. Its ability to quantify limb stiffness with high precision enhances the evaluation of treatment strategies in CP models, paving the way for improved therapeutic development and outcomes.

## Background

Movement disorders such as cerebral palsy, stroke and spinal cord injury often include components of hypertonia, spasticity, dystonia and rigidity. To make advances in treating each disorder and its components, using animal models, deficits animal models must be carefully quantified using specialized devices. Measurement of torque is most useful for stiffness, rigidity and dystonia (particularly hypertonia). Previously, torque measurements were used to characterize hypertonia in animals (Drobyshevsky et al., 2012), but details on how such a device was constructed were unclear, and no device for torque measurement in animals is commercially available. Therefore, we are sharing methods to create a low-cost, open source device that we use to evaluate limb stiffness in a rabbit model of cerebral palsy, in hopes that it will be useful to others studying motor dysfunction in animal models.

While there are many methods used for quantifying hypertonia, spasticity, dystonia and rigidity across humans and animals, few methods are consistently used in both clinical and preclinical studies. Spasticity is historically defined as velocity-dependent increase in muscle stretch reflexes associated with increased muscle tone as a component of upper motor neuron syndrome (Lance, 1980; Sanger et al., 2003). There is less agreement on the exact definitions of dystonia and rigidity, and whether they are different phenomena, but both involve stretch-and-effort-unrelated, involuntary muscle activity including co contraction of antagonistic muscle groups (Sanger et al., 2003; Lorentzen et al., 2018). Clinicians may use one or more diagnostic tools or assessments available to classify spasticity, dystonia and rigidity (Australian Spasticity Assessment Scale [ASAS] (Love et al., 2016), Barry Albright Dystonia Scale [BADS] (Barry, VanSwearingen, and Albright, 1999), Dyskinesia Impairment Scale [DIS] (Monbaliu et al., 2012), Dyskinetic Cerebral Palsy Functional Impact Scale [D-FIS], and the Hypertonia Assessment Tool [HAT] (Marsico et al., 2017; Stewart et al., 2021). The gold standard is assessment of neurologic exam videos by expert consensus (Albanese et al., 2013; Luc and Querubin, 2017; Albanese et al., 2023), but this qualitative analysis is difficult to translate to animal models. Another potential complication in rigorous quantification is the high degree of variability in presentation, including which limbs are affected, the severity of the impairment, and under which conditions the disordered movement is most prominent. Thus, it is important to evaluate both sustained and stretch-evoked stiffness of the limbs for clinical categorization, as well as to assess any changes that might result from different activities or therapy.

Historically, modified Ashworth and Tardieu scales have been used to quantify dysfunction of the affected limbs in cerebral palsy. They are easily accessible ways to score the stiffness of a joint during passive movement (Akpinar et al., 2017; Held and Pierrot-Deseilligny, 1969). These assessments do not require any specialized equipment and are quick and simple to perform, making them popular choices in clinics and research labs. Modified Ashworth simply involves rating the stiffness or resistance to passive movement around a joint on a subjective scale of 0–4. The Tardieu scale improves upon the Ashworth scale in the inclusion of velocity as a factor that can modify stiffness due to the activation of stretch receptors (Patrick and Ada, 2006), but still depends on the clinician to rotate the limbs of the subject at 3 (subjective) speeds and measure the catch angle with a goniometer. Dynamic range of motion and the pendulum tests are similar but both typically include use an accelerometer to more precisely measure the acceleration of the limb and to control for varying range of motion in slow/passive conditions and fast conditions which evoke a spastic catch (Jóźwiak, 2001; Szopa et al., 2014; Fowler et al., 2000). However, there are factors in all of the above scales that are highly subject to variability between those performing the scoring, including the speed of rotation around the joint, and the impact that the rater has in assessing spasticity (Nourizadeh et al., 2024).

Quantitative modes of testing hypertonia and rigidity have also been developed, including measurement of active vs. passive contributions to stiffness, but rarely are these tests applied to animal models of cerebral palsy. Noteworthy among clinical tests for stiffness are measurements of muscle elasticity using acoustic radiation force impulse (ARFI) elastography (Bilgici et al., 2018), real time sonography (Park and Kwon, 2011) and shear wave ultrasound elastography (Lee et al., 2016; Kwon and Kwon, 2021); measurements of active contractions of the agonist and/or antagonist muscles vs. passive stiffness of the joint or muscle using electromyograms (EMGs); measurement of catch angles more precisely than Tardieu using inertial sensors (van den Noort et al., 2009); and measurement of the contribution of stretch reflexes using tendon indentation (Chardon, Suresh, and Rymer, 2010) via an actuator. Many labs use torque to assess active and passive muscle/joint stiffness, starting with foundational studies in the laboratories of Thomas Sinkjær, Zev Rymer, Jens Bo Nielsen, and Robert Kearney (Galiana, Fung, and Kearney, 2005; Mirbagheri et al., 2007; Mirbagheri, Barbeau, and Kearney, 2000; Sinkjær and Magnussen, 1994; Nielsen et al., 1994; Nielsen and Kagamihara, 1993; Toft, Sinkjær, and Andreassen, 1989; Powers, Campbell, and Rymer, 1989; Powers, Marder-Meyer, and Rymer, 1988; Sinkjær et al., 1988). With use of a torquemeter, it is possible to avoid the subjectiveness of the modified Ashworth and better control the speed of rotation around a joint than the modified Tardieu scale. This is particularly important for clinical assessment of cerebral palsy, in which the speed of rotation around the joint can have an impact on the evoked spasticity (Wu et al., 2010; Sloot et al., 2021). Many studies use measurement of torque in and out of the laboratory setting, during passive leg movement, while evoking reflex responses or other perturbations and recording muscle activity using EMGs (Galiana, Fung, and Kearney, 2005; Mirbagheri et al., 2007; Mirbagheri, Barbeau, and Kearney, 2000; Wu et al., 2010; Drobyshevsky et al., 2015; Willerslev-Olsen et al., 2018). Some have used a motorized instrument to study passive resistance during ankle dorsiflexion at controlled speeds (Åhblom et al., 2024). One study even assessed leg stiffness using a robotic-assisted gait orthosis (Derrick et al., 2004) with a built-in torquemeter. Similar devices have also been employed in the study of stroke (Pennati et al., 2023), Parkinson’s Disease (Linn-Evans et al., 2020) and ALS (Stikvoort García et al., 2024). However, very few studies have applied measurement of torque to animal models of cerebral palsy, or spasticity more generally, making it more difficult to compare clinical and preclinical data. Careful use of joint torque measurements, along with other measurements can allow quantification of sustained rigidity of limbs, velocity-dependent spasticity, the active and passive properties that contribute to each, and their development over time in animal models (Cavarsan, Gorassini, and Quinlan, 2019).

**FIGURE 1:**
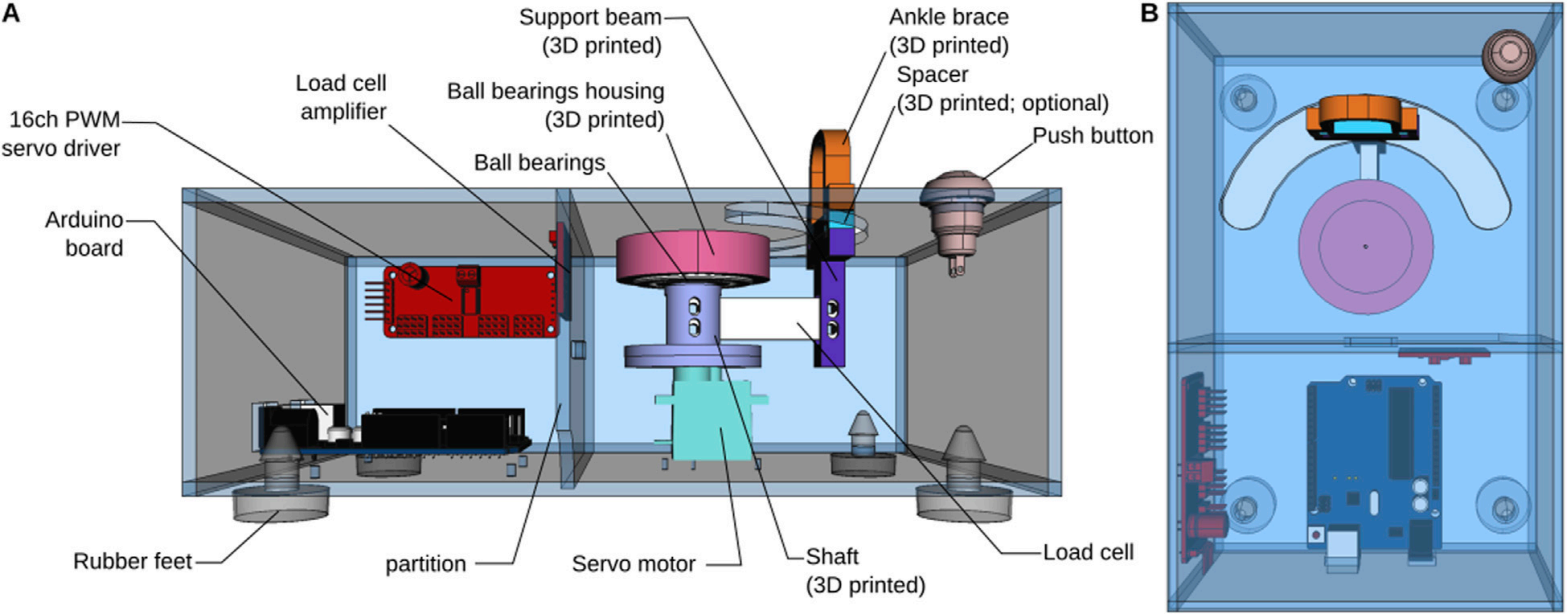
Overview of the torque meter design. **(A)** View from the front, with the front side of the enclosure removed. **(B)** Top view.

In this paper, we describe the construction and specialization of a torquemeter (Figure 1), appropriately sized for newborn rabbits (or “kits”) between 1 and 18 days old, and which can be used to compare joint stiffness in an animal model of cerebral palsy. Newborn rabbits exposed to prenatal hypoxia-ischemia have prominent brain and spinal cord damage and motor dysfunction that is likely best described as rigidity or dystonia since there is not a prominent “catch angle” and limbs are somewhat fixed in abnormal postures (Cavarsan, Gorassini, and Quinlan, 2019). First, we will provide step by step instructions on how to build this device, how to calibrate the readings and how to operate it with live rabbit kits. We will then characterize the performance of the device in terms of speed and range of motion. Finally, we will show sample data that we have collected from our rabbits using our low cost, open source device.

## Methods

All experiments described in this paper were performed according to guidelines of the National Institutes of Health guide for the care and use of Laboratory animals (NIH Publications No. 8023, revised 1978) and have been authorized by the University of Rhode Island’s Animal Care and Use Committee (Protocol AN1718-001). Details of the procedures have been published previously (Cavarsan, Gorassini, and Quinlan, 2019; Reedich et al., 2023; Derrick et al., 2004).

### Build instructions

All the files used for 3D printing and laser cutting are open-source and freely available (see Data availability statement). A bill of material for building the device is provided in Supplementary Table S1.

#### Assemble the partition

**FIGURE 2:**
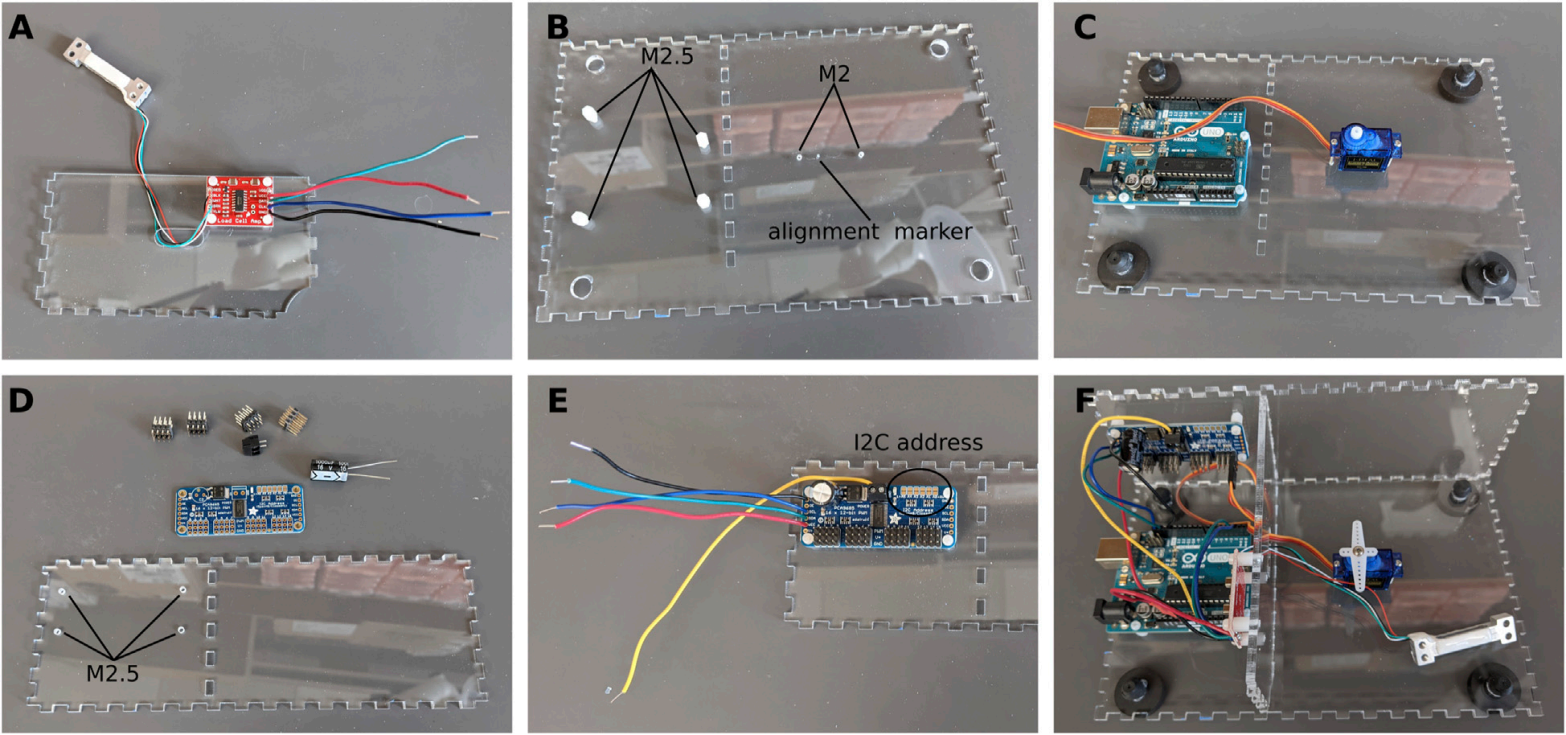
Assembly of the electronics. **(A)** Load cell and amplifier. **(B)** Preparing the bottom plate. **(C)** Installing the servo motor and Arduino board. **(D)** Preparing the PWM driver. **(E)** PWM driver in place. **(F)** All the electronics are in place.

The partition is used to separate the electronic components and protect them from fur and other projections that might fall through the opening on the top of the enclosure. Cut M2.5 threads in the four holes. Install nylon standoffs. Solder the Load Cell to the Load Cell Amplifier following the wire color code (the load cell we are using does not have a yellow wire. If using a different load cell, refer to the manufacturer’s datasheet for proper assembly). Solder four wires (approximately 10 cm in length) to the four pads on the other side of the amplifier (VCC and VDD are tied together). Attach the amplifier to the acrylic using the nylon standoffs. Slide the Load Cell through the slot in the partition wall so that it ends up on the other side of the partition (Figure 2A).

#### Assemble the bottom plate

Tap the four holes for the Arduino Uno with M2.5 threads. Tap the two holes for the servo motor with M2 threads (Figure 2B). Install nylon standoffs and secure the Arduino Uno on the standoffs. Install the servo motor so that the axis is over with the alignment marker (this marker is included in the enclosure file and will be cut by the laser cutter) and the wires are pointing towards the Arduino Uno. Secure the servo motor using the M2 × 22 screws (Figure 2C). Snap in the rubber feet in the holes at each corner.

#### Assemble the back wall and PWM driver

Tap the four holes on the back wall of the enclosure with M2.5 threads for the nylon standoffs (Figure 2D). Assemble the PWM driver as per the instructions of the manufacturer. We added a large 1000 μF electrolytic capacitor to the board to prevent any dip in supply voltage. Solder 4 wires (approximately 10 cm in length) to the VCC, SCL, SDA, and GND pads on the left of the board. Insert a fifth wire (15 cm in length) to the V+ terminal block at the top of the board. Make a note of the I2C address of the board as it will be needed later in the Arduino sketches. By default, the address is 0×40 (Figure 2E).

#### Put the three pieces together

Using cyanoacrylate glue, assemble the three parts above as shown in Figure 2F. Connect all wires to the Arduino Uno as shown in the schematics. Make a note of the channel to which the servo motor is connected. In our case, the servo motor is connected to channel 15 (Figure 2F).

At this stage the three other walls can be glued in place. The box should now be fully assembled apart from the top lid.

#### Lid

**FIGURE 3:**
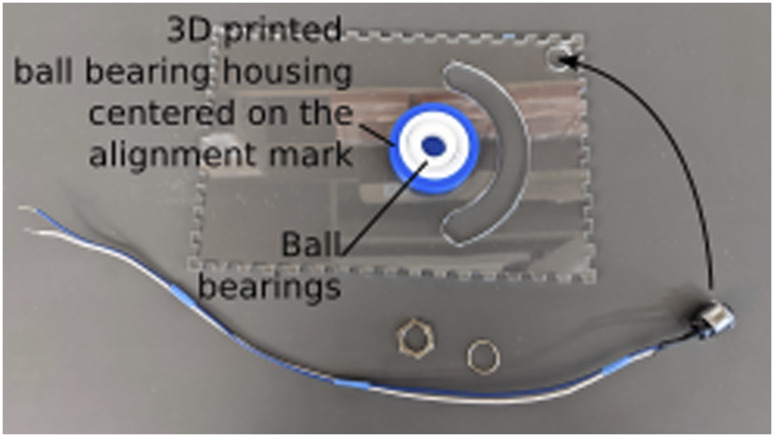
Assembly of the lid. The lid is placed upside-down on a flat surface to glue the ball bearing housing and insert the ball bearings. Care is taken to align the ball bearing housing with the alignment mark cut into the lid as part of the laser cutting process.

Using a push pin to align the center of the 3D printed ball bearing housing with the alignment marker (Figure 2B), glue the housing to the bottom of the lid using cyanoacrylate glue. Once the glue is dry, insert the ball bearings in the housing (Figure 3). Using two 20–25 cm long wires, assemble the push button and install it in the hole in the corner of the lid.

#### Support beam and ankle brace

**FIGURE 4:**
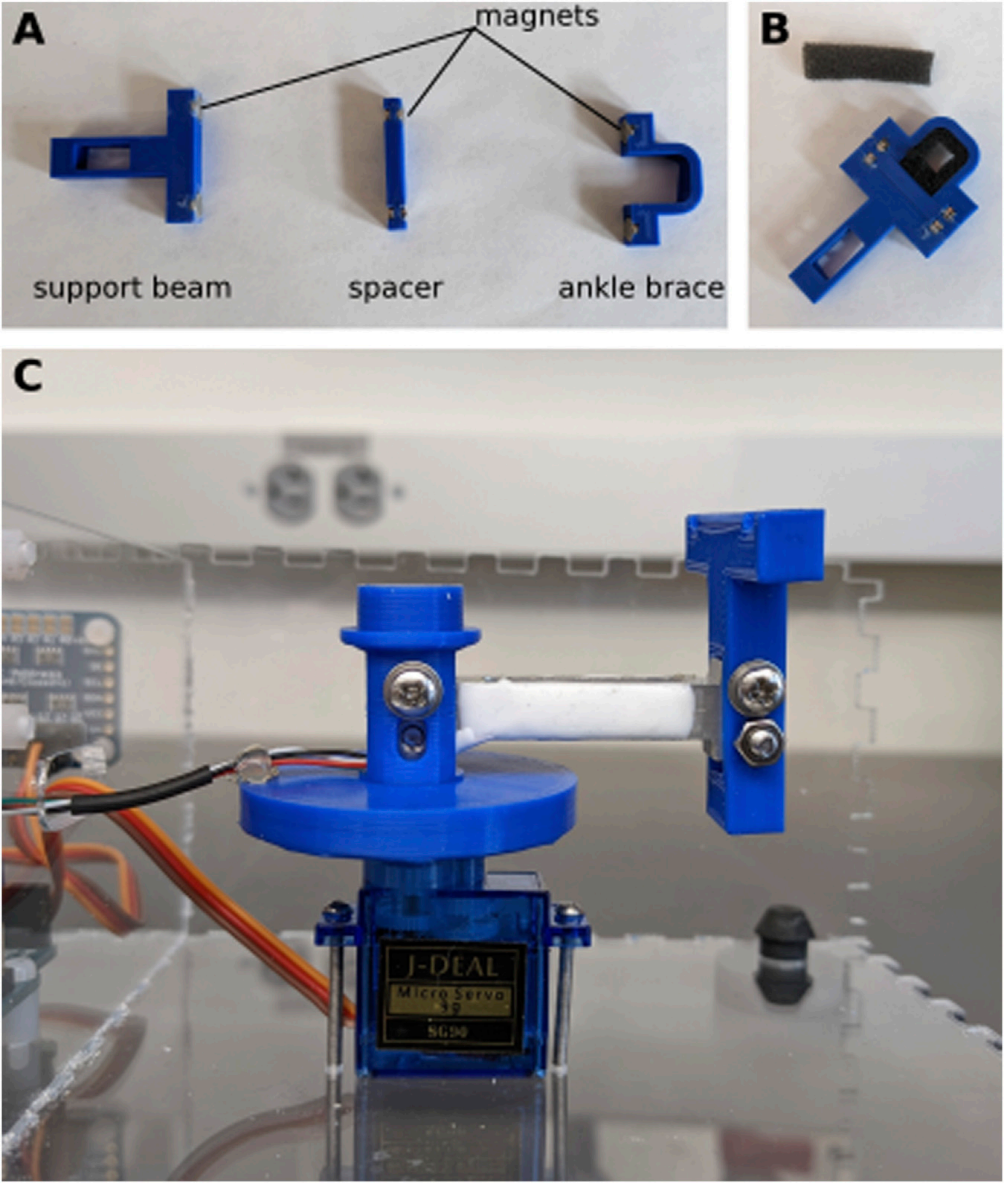
Assembly of support beam. **(A)** Neodymium magnets are glued into the 3D printed pieces. **(B)** Small pieces of foam are glued to the inside of the ankle brace and the top of the spacer. **(C)** The load cell is secured to the shaft of one side and the support beam on the other.

Using cyanoacrylate glue, glue neodymium magnets in the slots of the support beam, spacer, and ankle brace. Pay attention to the orientation of the magnets so that the pieces all snap together (Figure 4A). When the glue has fully cured, cut a small strip of foam and glue it to the inside of the ankle brace, and on the top of the spacer (Figure 4B). The foam is about 5 mm thick, and should leave a small space at the center of the ankle brace for the ankle. The foam prevents bruising of the ankle during the experiment.

Attach the support beam to the end of the load cell (the end without wires) using two M3 bolts and nuts. Place the other end of the load cell (side with the wires) inside the shaft and secure it with two M3 bolts and nuts (Figure 4C). To provide strain relief, use hot glue to attach the wires to the brim of the shaft.

#### Upload firmware

**FIGURE 5:**
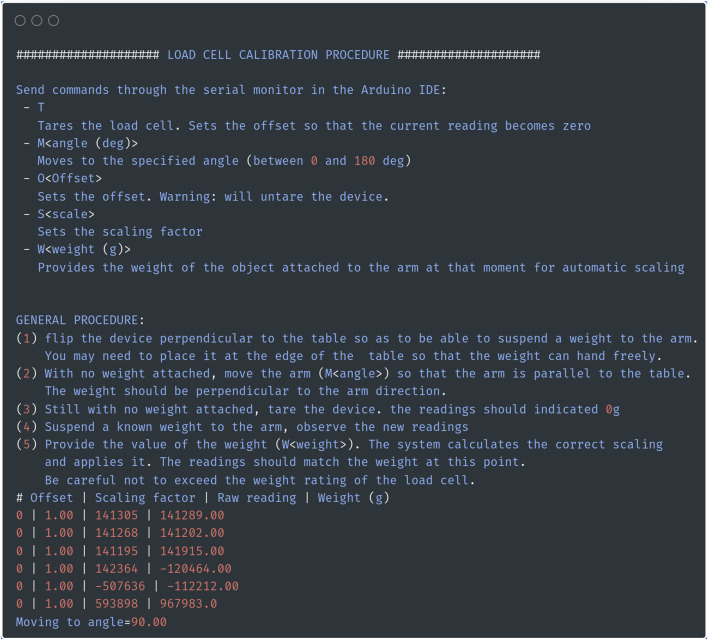
Position the servo motor in the right position. Screenshot of the serial output of the calibration sketch. In addition to providing calibration, the sketch allows you to move the servo motor to the desired rest position by sending the command “M<angle>” through the serial monitor.

Before continuing with the build, edit the Arduino sketch “CalibTorqueMeter.ino” on your computer. Make sure that the I2C address for the PWM (constant “PWM_ADDRESS”) and the channel to which the servo motor is connected (constant “SERVO_PIN”) are correct. The firmware requires two external libraries, which can be installed using the library manager in the Arduino IDE (bogde, 2014; Adafruit, 2023). Compile and upload the sketch to the Arduino Uno board, then open the serial monitor tool of the Arduino IDE. You should obtain a screen similar to the one shown in Figure 5. Send the command “M90” to move the servo motor to 90° (substitute 90 by the angle you would like to use as the resting, baseline position of the arm when it is not moving).

#### Finish assembly

Place the lid close to the assembled box. Route the wires of the button along the front wall and through the opening at the bottom corner of the partition wall. The two wires are plugged into Pin 11 and GND of the Arduino board (it does not matter which wire is connected to which pin).

Now that the servo motor is in the correct position, seat the shaft on top of the servo motor (Figure 4C) and close the lid so that the top of the shaft is inserted into the ball bearings. The apparatus is now fully assembled. We recommend not gluing the lid in case adjustments need to be made, but it can easily be taped in place.

#### Calibration

**FIGURE 6:**
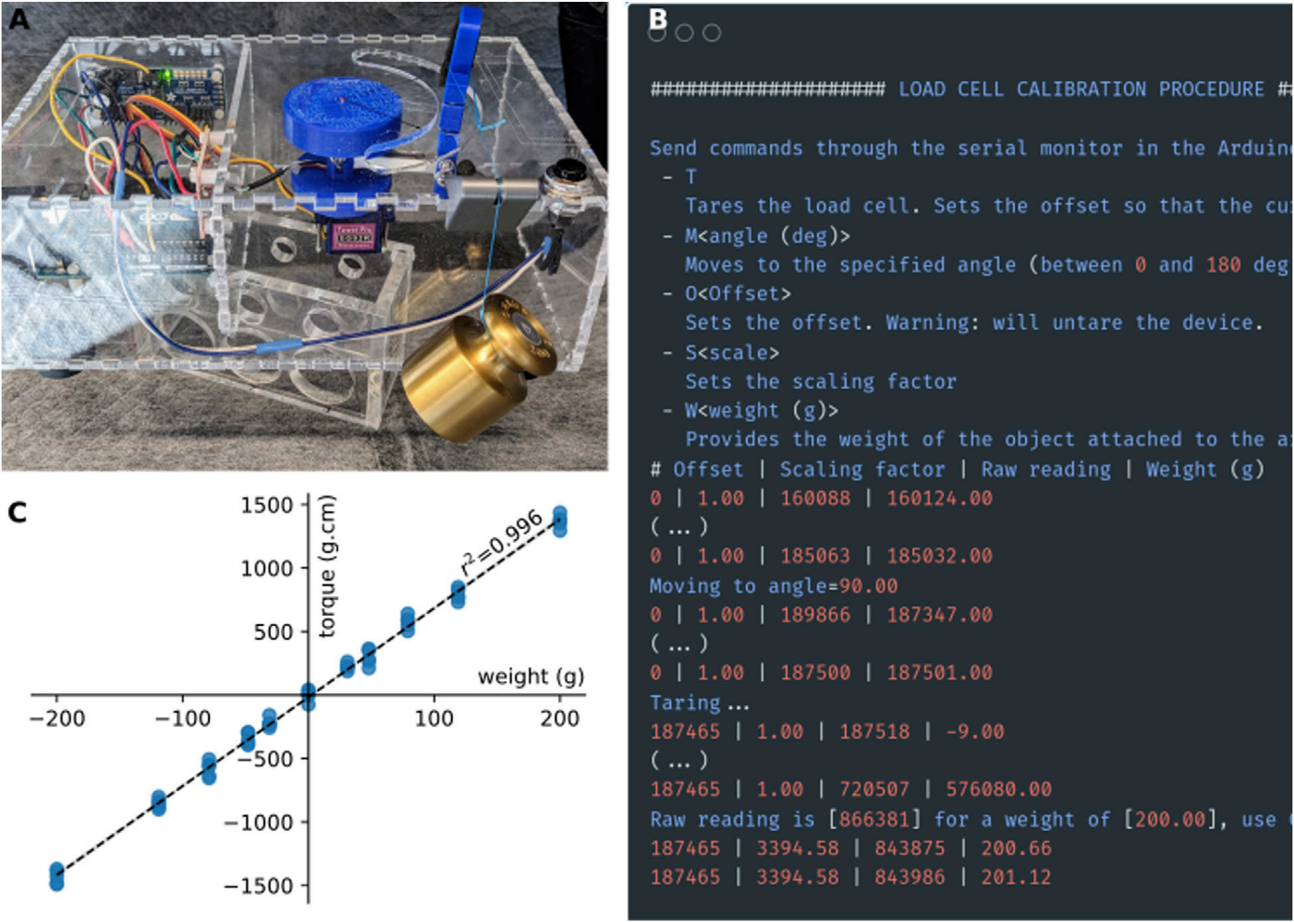
Calibration procedure. **(A)** For calibration, a weight is attached to the strain gauge via a thread and a pulley system. The torquemeter was elevated above the table to prevent the weight from touching table **(B)**. Screenshot of the output of the calibration sketch. **(C)** Calibration curve of the strain gauge obtained using a series of weights between 10 and 200 g. Positive weights on the x-axis indicate force applied in the clockwise direction, while negative values indicate forces applied in the counter-clockwise direction.

The first step is to calibrate the load cell so that the system outputs a value in gram-force or Newton (as desired). With the Arduino sketch “CalibTorqueMeter.ino” still loaded, as in Figure 4, flip the box to its side (Figure 6A). If it is not already, move the servo motor by sending the string “M<angle value>” (i.e., “M90” to move to the 90deg position) through the serial monitor, so that the load cell is parallel to the ground and the force exerted on the load cell is vertical (Figure 6B). With nothing but the ankle brace attached to the load cell, tare the load cell by sending a “T” through the serial monitor (Figure 6B). Attach a weight (appropriately sized for the maximum capacity of the load cell) to the ankle brace (Figure 6A). Send the command “W<weight in grams>” (i.e., “W200” for a 200 g weight). Check that the value displayed in the serial monitor [column “Weight (g)”] matches the weight that you used. The serial monitor will display a message showing the value of the scaling factor to use (3,394.58 in the example shown in Figure 6B). Calibration with only one weight is sufficient given the highly linear behavior of the strain gauge (Figure 6C).

Edit the Arduino sketch “ArduinoTorqueMeter.ino” on your computer. As previously, make sure the constants defined at the top of the sketch (PWM_ADDRESS, SERVO_PIN, BAUDRATE, LOADCELL_DOUT_PIN, LOADCELL_CLK_PIN, BTN_PIN) match the configuration of your device. Enter the calibration factor obtained during the calibration procedure as the constant CALIBRATION_FACTOR (line 46). Compile and upload the sketch to the Arduino board. The device is now ready for use.

### Operation instructions

**FIGURE 7:**
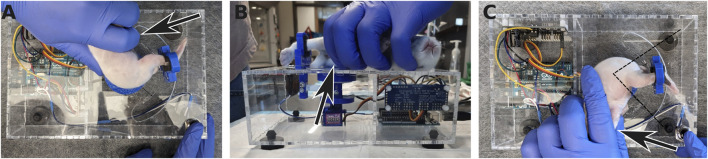
Positioning the rabbit kit in the apparatus. **(A)** The rabbit kit is laid down on the top of the torquemeter with its hip at the center of the axis of rotation. **(B)** The foot on the bottom is tucked under the body and held in place to avoid kicking (arrows). The leg on top is extended and secured in the ankle brace. **(C)** Once measurements for the first leg have been acquired, the rabbit is flipped on the other side and the same procedure is performed with the other leg, now on top. The dashed lines indicate the range of motion of the leg during the measurements.

The subject is laid on its side on top of the enclosure, taking care to place the hip joint at the center of rotation of the apparatus (Figure 7). The device is used in the same way whether the subject is awake or anesthetized (as would be done to measure non-neurally-driven contributions to stiffness). If the subject is awake, habituation to the device may require a few minutes. However, the whole advantage of the mechanical device is to remove variability due to the experimenter. The sole role of the experimenter is to gently hold the bunny kit in place, while the device goes through the motions and measurements without human intervention. One leg is extended and held at the ankle by a 3D-printed brace. This brace is held magnetically to a support beam, attached to one end of a rectangular load cell. The magnets were chosen so that the brace can be installed easily and one-handed, while the other hand is gently restraining the subject. A critical part of a good restraint is to tuck and hold the bottom leg under the body of the subject in order to avoid kicks with the leg not being tested (Figure 7). In case of kicking from either of the two legs, the trial is discarded and a new series of stretches is started until about 10 sweeps can be recorded without interference.

The operation of the device is very simple. When the button on the lid is pressed, the arm makes a predefined back-and forth movement (the range is defined in the “ArduinoTorqueMeter.ino” sketch file). The movement continues as long as the button is pressed. While the arm is moving, the torque exerted on the load cell is measured and the angle and the torque values are output as tabular data on the serial port. Any software able to capture the data stream from the serial port could be used to record the data. We provide a simple software called “TorquePlotter” that allows saving the stream of data to a text file for offline analysis as well as plotting the data in real time for quick visualization.

**FIGURE 8:**
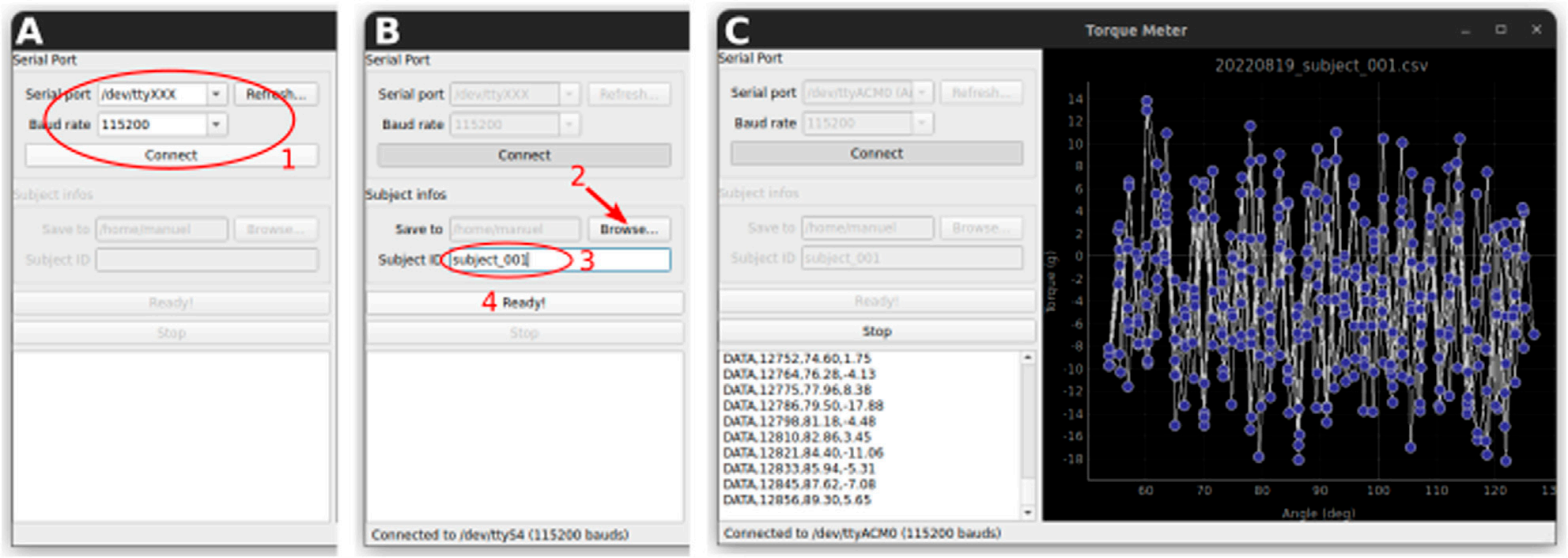
Data acquisition with the TorquePlotter software. **(A)** Setup of the serial connection **(B)**. Select a folder for saving the data and a unique identifier for the subject that will be used to generate the data filename. **(C)** The data is streamed to the software and plotted in real time.

1. Start the TorquePlotter software. Choose the appropriate serial port and baud rate and click connect (Figure 8A).
2. Select the folder in which the data will be saved (Figure 8B).
3. Enter the subject ID. This ID will form part of the data file name (in addition to the date and time) (Figure 8B).
4. Click “Ready!” (Figure 8B). The software is now ready to receive data from the Torque Meter.
5. Install the subject in the device. The hip joint should be placed at the center of the semicircle cutout, and the ankle should be held securely in the ankle brace. To accommodate subjects of different sizes, spacers of various heights are available.
6. Press the button on the Torque Meter. Data is streamed to the TorquePlotter software (Figure 8C). We typically collect 10 rotations per leg per subject.
7. When done, press the “Stop” button in the TorquePlotter software. This closes the data file and saves it to disk.
8. Repeat with the remaining subjects.

### Statistical analysis

To evaluate device performance, the travel duration and travel distance of the ankle brace were recorded for more than 10 cycles at each preset speed. We assessed the accuracy of the system by comparing the effective cycle period and effective cycle amplitude to their corresponding target values. We tested whether the measured values fell within a predefined acceptable margin of error (±5%) from the corresponding theoretical value. Specifically, we used a one-sided one-sample t-test to determine whether the absolute difference between the measured and theoretical value was significantly less than 5% of the theoretical value. This test was performed separately for each speed condition and for each metric (cycle period and amplitude). A Bonferroni correction was applied to control for multiple comparisons, with significance set at α = 0.05/n (where n = 15 is the number of speed conditions). All tests were performed with python 3.12.8 and pingouin 0.5.5 (Vallat, 2018).

## Results

The torque meter is, by design, very simple. It uses off-the-shelf components that are robust, inexpensive, and pre-assembled, minimizing the skill-level required to assemble the device, and the risk of mistakes during assembly.

The torque meter consists of an enclosure, on the top of which a semi-circular cutout has been cut. The neonate rabbit subject (hereafter “kit”) is placed on top of the device and one of its legs is secured in an ankle brace (see below, Operation instructions) attached to one end of a load cell. The other end of the load cell is attached to a hobby servo motor in such a way that rotation of the servo causes the load cell to describe an arc of a circle, thereby moving the leg of the subject at the hip joint, along the semi-circular cutout of the enclosure (Figure 1). The load cell is secured to the servo by a 3D-printed shaft. The shaft is directly attached to the servo’s horn on one end and held vertically by a ball bearing attached to the top of the enclosure. We have observed that controlling the servo motor directly using one of the Arduino’s PWM pins while at the same time communicating with the load cell amplifier (see below) resulted in unacceptable jerks in the movements of the load cell. These jerks could elicit potential strong, unpredictable, and uncontrollable muscle contractions, particularly in cerebral palsy subjects, which could perturb the measurements. To ensure a smooth movement, we therefore chose to control the servo through a separate i2c-controlled PWM driver with a built-in clock. Although we are only controlling one servo, the model that we chose has 16 channels, which may seem excessive. However, as explained above, this driver has the advantage of being mostly pre-assembled, while remaining inexpensive, which makes it very easy to use even for novices.

The strain exerted on the load cell causes small deformations of the strain gauges, and therefore a small change in electrical resistance. This signal is measured, amplified, and digitized by the popular HX711 precision 24-bit analog to-digital converter. Again, this choice was made because there exist inexpensive boards with this chip and all its auxiliary components already pre-installed and configured, making it much easier to use. The data is sent as digital information to an Arduino, which then sends it to a recording computer.

Because the servo motor may draw more current than can be supplied by the Arduino +5 V pin, particularly when stalled, the servo is powered directly by the external power supply that also powers the Arduino board. The rotation of the servo motor is controlled by the Arduino. However, movements must be started only when the ankle of the subject is securely attached to the brace. Therefore, a button is conveniently placed on the top of the enclosure, close to the hand of the experimenter so that they may press the button with their free hand as soon as the subject is correctly positioned.

The whole system is supervised by an Arduino Uno, which, upon button press, initiates the rotation of the servo motor, collects the data from the load cell, and sends both data to a host computer via the serial line.

All the electronic components are bought pre-assembled. Only a handful of wires need to be soldered. The mechanical components are 3D-printed and can be adjusted to accommodate different sizes of animals. The enclosure is made of laser-cut acrylic, although a 3D printed version could easily be created, if a laser cutter is unavailable. All materials were 3D-printed in PLA and exhibited adequate strength of the age range studied here.

### Validation and characterization

To further validate and characterize the device, we evaluated the hardware’s capability to operate at different velocities. To do so, we obtained high-speed video recordings using a smartphone (Pixel 4a, Google LLC) in slow-motion mode, capturing footage at 240 frames per second (Google, 2020) of the movement of the brace relative to the center of rotation. Using automatic analysis of the recorded videos [DeepLabCut v.2.3.5 (Mathis et al., 2018)], we determined the actual angular displacement at different time intervals. To evaluate the hardware’s performance, we then compared the effective angular displacement against the expected trajectory (derived from the parameters entered in the code of the software, i.e., initial angle, maximal and minimal angle, and cycle period).

**FIGURE 9:**
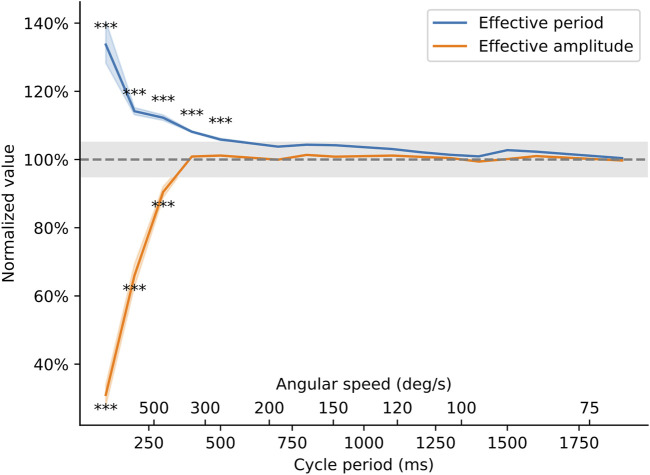
Behavior of the torquemeter at different cycle periods. The effective period and amplitude of the oscillations (as measured on the high-speed video recording) was compared to the theoretical movement of the arm as programmed in the Arduino sketch. Effective period is the period of the oscillatory movement of the ankle brace (in seconds) normalized by the value entered in the Arduino sketch. Effective amplitude is the peak-to-peak amplitude of the movement (in degrees) normalized by the amplitude entered in the Arduino sketch. The axes show how these two quantities vary as the speed of the torquemeter varies from 2 s/cycle (equivalent to an angular speed of 45 deg/s) to 200 ms/cycle (or an angular speed of approximately 700 deg/s). Asterisks indicate speeds at which the effective amplitude or effective period deviated by more than 5% away from their target value (p < 0.0033).

The results of this analysis are summarized in Figure 9. Below 500 m of cycle period, the amplitude of the movement becomes truncated compared to the desired amplitude, and the arm moves too slowly, resulting in longer oscillation periods than requested in the software. However, for cycle periods longer than 500 ms (corresponding to speeds below ∼300 deg/sec), the device is able to faithfully follow the movement that was programmed in software. Although the servo motor is unable to generate very fast oscillations, the device’s performance is amply sufficient to meet the speeds commonly cited in the literature [20–270 deg/sec (Wu et al., 2010; Cavarsan, Gorassini, and Quinlan, 2019)].

**FIGURE 10:**
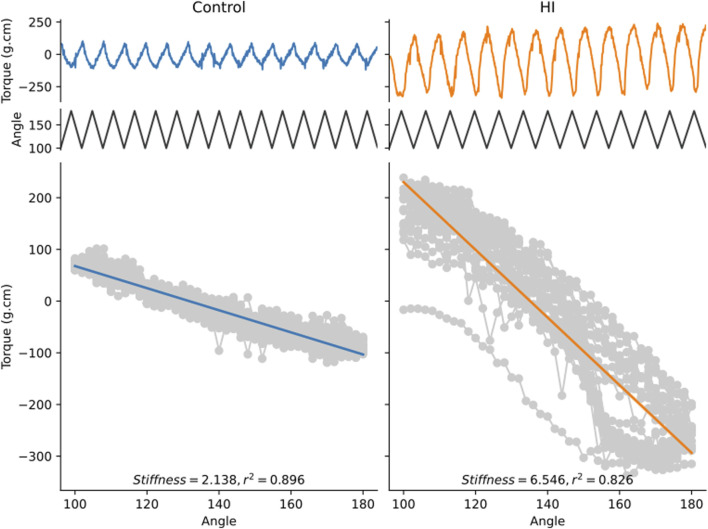
Examples of recordings obtained from a control kit and a kit having experienced a prenatal hypoxia-ischemia injury. Both kits were 11 days old on the day of the measurement. The top trace shows the torque value measured by the apparatus as a function of time. The middle trace shows the angular displacement of the ankle brace as a function of time. The bottom gray curve is the torque value plotted against the angular displacement at every point in time. The thick line is the linear best fit line. The stiffness value corresponds to the absolute value of the slope of the regression line.

Based on these results, we investigated the limb stiffness of control rabbit kits and rabbit kits having suffered a prenatal hypoxia-ischemia injury, a model of cerebral palsy (Derrick et al., 2004; Cavarsan, Gorassini, and Quinlan, 2019), in response to a passive stretch with a cycle period of 1 s. Figure 10 shows two examples of such recordings. The top traces are the raw data from the torquemeter, while the bottom traces show the torque values plotted against the angle of the ankle bracket. The limb stiffness corresponds to the absolute value of the slope of the linear regression line (Drobyshevsky et al., 2012). These traces are presented to illustrate the typical performance of the device during experimental use. These examples are not intended for comparative statistical analysis but rather serve to highlight the consistency and resolution of the measurements achievable in practice. The apparatus is capable of generating passive stretches of the kit leg around the hip and is capable of detecting differences in passive force between control animals and rabbit kit having experienced a prenatal hypoxia-ischemia injury.

## Discussion

We present a novel, low-cost, open-source device specifically developed for the investigation of hypertonia in a rabbit model of cerebral palsy. This device has several advantages: first, it is inexpensive and easy to build, making it accessible to researchers with limited budgets. Second, it is open source, meaning that the design files are freely available and can be modified to suit specific needs. Third, it is precise and reproducible, providing reliable measurements of torque and joint rotation speed. Finally, the device is designed for use with a rabbit model of cerebral palsy (and is an appropriate size for use in adult rats) and can be used in combination with EMG and other techniques, allowing researchers to study spasticity, dystonia, and rigidity in a controlled environment.

Previous studies using torque in animals have not generally provided a guide for construction of the torquemeters they have used. One torquemeter that was described in the literature was used to measure joint torque and sarcomere length in an *in vitro* study of the isolated frog leg (Lieber and Shoemaker, 1992; Lieber and Boakes, 1988). Another study briefly described a torquemeter they constructed for use in cats (Tarler and Mortimer, 2003). More closely related to the current study, torquemeters have been constructed for use in rats and rabbits (Just, Pawlak, and Heppelmann, 2000; Leumann et al., 2015; Usuba et al., 2007; Nakazato, Song, and Waga, 2007) but designed for use in terminal or anesthetized preparations, and/or without enough detail to fully replicate construction.

Rabbits exposed to prenatal hypoxia-ischemia have been described as hypertonic (Tan et al., 2005; Drobyshevsky et al., 2012; Drobyshevsky and Quinlan, 2017; Synowiec et al., 2019). Motor deficits are variable, but limb stiffness is most common and they are hyperreflexic based on several measures [larger H/M ratio of the Hoffman reflex, and less rate dependent depression (Synowiec et al., 2019)]. Previous work has demonstrated both neural and non-neural contributions to hypertonia by measuring torque before and after anesthesia (Synowiec et al., 2019). Brain injuries in the rabbits are variable: roughly one-quarter of the kits that show motor deficits have cortical damage, while damage to the thalamus, basal ganglia, midbrain and brain stem are more common (Drobyshevsky et al., 2012). Aligning the presentation of each rabbit to a more commonly used clinical definition would be helpful to put both the rabbit model and our experimental results in context for the field of cerebral palsy research. However, there is no broadly agreed upon definition of what constitutes hypertonia that is not velocity dependent (as it appears not to be in the rabbit). It has at times been defined as “spastic dystonia” (Lorentzen et al., 2018; Denny-Brown, 1966), and as either rigidity or dystonia (Jethwa et al., 2010). Relevant to this classification, in a consensus statement from 2003, Sanger and colleagues stated (Sanger et al., 2003)

“Spasticity” is defined as hypertonia in which 1 or both of the following signs are present: 1) resistance to externally imposed movement increases with increasing speed of stretch and varies with the direction of joint movement, and/or 2) resistance to externally imposed movement rises rapidly above a threshold speed or joint angle. “Dystonia” is defined as a movement disorder in which involuntary sustained or intermittent muscle contractions cause twisting and repetitive movements, abnormal postures, or both.

and rigidity was described as

hypertonia in which all of the following are true: 1) the resistance to externally imposed joint movement is present at very low speeds of movement, does not depend on imposed speed, and does not exhibit a speed or angle threshold; 2) simultaneous co-contraction of agonists and antagonists may occur, and this is reflected in an immediate resistance to a reversal of the direction of movement about a joint; 3) the limb does not tend to return toward a particular fixed posture or extreme joint angle; and 4) voluntary activity in distant muscle groups does not lead to involuntary movements about the rigid joints, although rigidity may worsen.

According to these classifications, we would classify rabbits showing motor dysfunction after prenatal hypoxia ischemia as having dystonia (involuntary sustained muscle contractions causing abnormal postures), not rigidity, due to the tendency of the affected limbs to return toward a particular fixed posture (point #3 above). Based on these guidelines the neonatal rabbits would not necessarily be classified as spastic, despite the fact that they are hyperreflexic based on several measures, because there is no catch angle and we have not yet observed any change in torque with speed. Measurement of torque as described here is well suited to distinguish between spasticity (because the torque measurement would capture a catch angle and a change in stiffness at different speeds) and dystonia (because torque can also measure a sustained or chronic increase in stiffness, as shown in Figure 10). Another aspect of dystonia is involuntary movement evoked by sensory input to an unrelated body part. In order to thoroughly measure this feature, several distant muscle groups could be recorded with surface electromyograms (EMG) simultaneously to torque measurement. Thus, it is possible to incorporate torque measurements as described here into careful assessments of motor dysfunction in a preclinical animal model.

Measurement of joint torque can improve our ability to study mechanisms of hypertonia. One way to assess other neural contributions to stiffness are to perform torque measurements before and after sedation or with EMGs to assess contribution of altered activity of motoneurons/motoneuron drive (Willerslev-Olsen et al., 2018; Willerslev-Olsen et al., 2013; Synowiec et al., 2019). Agonist/antagonist co-contraction can result in neurally-driven hypertonia that is not related to stretch reflex activity. Additionally, sustained, or chronic hypertonia could be mediated by non-neural factors. Passive stiffness in the connective tissue of muscles contributes significantly to muscle stiffness (Willerslev-Olsen et al., 2018; Willerslev-Olsen et al., 2013). Spasticity is presumably mediated through disinhibition of spinal reflexes by loss of corticospinal-driven inhibitory tone in the spinal cord, though this has not been directly tested. Performing torque measurements before and after dorsal rhizotomy can distinguish between reflex-driven hypertonia and non-reflex driven hypertonia (Denny-Brown, 1966). Thus, measurement of torque in different experimental conditions can be used to assess relative contributions from these mechanisms in animal models.

Here we share here the methods to construct a torque meter easily and at low cost, in hopes that the availability of this device in research labs will advance our understanding of the mechanisms of hypertonia, and more accurately distinguish clinical presentation of rigidity, spasticity and dystonia. The device is, by design, easy to build and easy to use. A small period of habituation and training is nevertheless required for the experimenter. The only difficulty in using this device is to gently handle the kit to be able to place its foot in the brace, while at the same time preventing kicking (either using the foot in the brace or the opposite limb). With training and patience, it is possible to gently cup the body in one hand, restrain the opposite limb with the thumb of that same hand, leaving one hand free to manipulate the limb to be tested, place the foot in the brace using the convenient magnetic coupling, and press on the go button once everything is in place. Moreover, as this operation is repeated over several days, animals become accustomed to being manipulated and easier to handle.

One of the limitations of the current design is that it is devised for testing stiffness in the hindlimbs only. However, similarly to human patients (Sanger et al., 2003), rabbit kits can develop hypertonia in both forelimbs and hindlimbs after hypoxia-ischemia injury (Drobyshevsky et al., 2012). A future direction for this device is therefore to adapt it to allow it to be used for both limbs.

## Conclusion

This paper presents a novel, low-cost, open-source device specifically developed for the investigation of hypertonia in a rabbit model of cerebral palsy. This device is inexpensive and easy to build, making it accessible to researchers with limited budgets. It is open source, meaning that the design files are freely available and can be modified to suit specific needs. It is precise and reproducible, providing reliable measurements of torque and joint rotation speed.

This torque meter offers researchers an accessible and reliable tool for preclinical movement disorder research. Its ability to quantify limb stiffness with high precision enhances the evaluation of treatment strategies in Cerebral Palsy models, paving the way for improved therapeutic development and outcomes.

## Data Availability

Publicly available datasets were analyzed in this study. This data can be found here: https://zenodo.org/doi/10.5281/zenodo.10393564.

## References

[B1] Adafruit (2023). “PWM servo driver library.” C++. Adafruit industries. Available online at: https://github.com/adafruit/Adafruit-PWM-Servo-Driver-Library.

[B2] Åhblom A. Pontén E. Destro A. Petersson S. von Walden F. Wang R. (2024). Exploration of the triceps surae muscle in ambulatory children with cerebral palsy using instrumented measurements of stiffness and diffusion tensor magnetic resonance imaging for muscle architecture. BMC Musculoskelet. Disord. 25 (1), 803. 10.1186/s12891-024-07890-4 39394126 PMC11468337

[B3] Akpinar P. Atici A. Ozkan F. U. Aktas I. Kulcu D. G. Sarı A. (2017). Reliability of the modified Ashworth scale and modified Tardieu scale in patients with spinal cord injuries. Spinal Cord. 55 (10), 944–949. 10.1038/sc.2017.48 28485384

[B4] Albanese A. Bhatia K. Bressman S. B. DeLong M. R. Fahn S. Fung V. S. C. (2013). Phenomenology and classification of dystonia: a consensus update. Mov. Disord. 28 (7), 863–873. 10.1002/mds.25475 23649720 PMC3729880

[B5] Albanese A. Bhatia K. P. Cardoso F. Comella C. Defazio G. Fung V. S. C. (2023). Isolated cervical dystonia: diagnosis and classification. Mov. Disord. 38 (8), 1367–1378. 10.1002/mds.29387 36989390 PMC10528915

[B6] Barry M. J. VanSwearingen J. M. Albright A. L. (1999). Reliability and responsiveness of the barry–albright dystonia scale. Dev. Med. and Child Neurology 41 (6), 404–411. 10.1017/S0012162299000870 10400175

[B7] Bilgici M. C. Bekci T. Ulus Y. Bilgici A. Tomak L. Selcuk M. B. (2018). Quantitative assessment of muscle stiffness with acoustic radiation force impulse elastography after botulinum toxin A injection in children with cerebral palsy. J. Med. Ultrasonics 45 (1), 137–141. 10.1007/s10396-017-0780-y 28271231

[B8] bogde (2014). 2023. “HX711.” C++. Available online at: https://github.com/bogde/HX711.

[B9] Cavarsan C. F. Gorassini M. A. Quinlan K. A. (2019). Animal models of developmental motor disorders: parallels to human motor dysfunction in cerebral palsy. J. Neurophysiology 122 (3), 1238–1253. 10.1152/jn.00233.2019 31411933 PMC6766736

[B10] Chardon M. K. Suresh N. L. Rymer W. Z. (2010). “An evaluation of passive properties of spastic muscles in hemiparetic stroke survivors,” in Annual International Conference of the IEEE Engineering in Medicine and Biology Society. IEEE Engineering in Medicine and Biology Society. Annual International Conference 2010, Buenos Aires, Argentina, 31 August 2010 - 04 September 2010 (IEEE), 2993–2996. 10.1109/IEMBS.2010.5626155 21095718

[B11] Denny-Brown D. (1966). The cerebral control of movement. Liverpool, United Kingdom: Liverpool University Press.

[B12] Derrick M. Luo N. L. Bregman J. C. Jilling T. Ji X. Fisher K. (2004). Preterm fetal hypoxia-ischemia causes hypertonia and motor deficits in the neonatal rabbit: a model for human cerebral palsy? J. Neurosci. Official J. Soc. Neurosci. 24 (1), 24–34. 10.1523/JNEUROSCI.2816-03.2004 PMC6729589 14715934

[B13] Drobyshevsky A. Derrick M. Luo K. Zhang L.-Q. Wu Y.-N. Honda Takada S. (2012). Near-term fetal hypoxia–ischemia in rabbits: MRI can predict muscle tone abnormalities and deep brain injury. Stroke 43 (10), 2757–2763. 10.1161/STROKEAHA.112.653857 22829546 PMC3458142

[B14] Drobyshevsky A. Quinlan K. A. (2017). Spinal cord injury in hypertonic newborns after antenatal hypoxia-ischemia in a rabbit model of cerebral palsy. Exp. Neurol. 293 (July), 13–26. 10.1016/j.expneurol.2017.03.017 28347765 PMC5509441

[B15] Drobyshevsky A. Silvia H. T. Luo K. Derrick M. Lei Y. Quinlan K. A. (2015). Elevated spinal monoamine neurotransmitters after antenatal hypoxia-ischemia in rabbit cerebral palsy model. J. Neurochem. 132 (4), 394–402. 10.1111/jnc.12997 25421613 PMC4329027

[B16] Fowler E. G. Nwigwe A. I. Ho T. W. (2000). Sensitivity of the pendulum test for assessing spasticity in persons with cerebral palsy. Dev. Med. and Child Neurology 42 (3), 182–189. 10.1017/S0012162200000323 10755458

[B17] Galiana L. Fung J. Kearney R. (2005). Identification of intrinsic and reflex ankle stiffness components in stroke patients. Exp. Brain Res. 165 (4), 422–434. 10.1007/s00221-005-2320-z 15991034

[B18] Google (2020). Pixel 4a hardware specs - Google store. Available online at: https://web.archive.org/web/20200804002340/https://store.google.com/product/pixel_4a_specs August 4, 2020).

[B19] Held J.-P. Pierrot-Deseilligny E. (1969). “Rééducation motrice des affections neurologiques,” in Précis du praticien (Paris: Baillière Paris).

[B20] Jethwa A. Mink J. Macarthur C. Knights S. Fehlings T. Fehlings D. (2010). Development of the hypertonia assessment tool (HAT): a discriminative tool for hypertonia in children. Dev. Med. Child Neurology 52 (5), e83–e87. 10.1111/j.1469-8749.2009.03483.x 20540176

[B21] Jóźwiak M. (2001). The clinical evaluation of spasticity: a methodology for the orthopaedic examination of children with cerebral palsy. Ortop. Traumatol. Rehabil. 3 (4), 490–495. 17984904

[B22] Just S. Pawlak M. Bernd H. (2000). Responses of fine primary afferent nerve fibres innervating the rat knee joint to defined torque. J. Neurosci. Methods 103 (2), 157–162. 10.1016/S0165-0270(00)00310-1 11084208

[B23] Kwon D. R. Kwon D. G. (2021). Botulinum toxin a injection combined with radial extracorporeal shock wave therapy in children with spastic cerebral palsy: shear wave sonoelastographic findings in the medial gastrocnemius muscle, preliminary study. Child. Basel, Switz. 8 (11), 1059. 10.3390/children8111059 PMC8622460 34828772

[B24] Lance J. W. (1980). “Symposium synopsis,” in Spasticity: disordered motor control.

[B25] Lee S. S. M. Gaebler-Spira D. Zhang L.-Q. Rymer W. Z. Steele K. M. (2016). Use of shear wave ultrasound elastography to quantify muscle properties in cerebral palsy. Clin. Biomech. 31 (January), 20–28. 10.1016/j.clinbiomech.2015.10.006 PMC4729598 26490641

[B26] Leumann A. Fortuna R. Leonard T. Valderrabano V. Herzog W. (2015). Tibiofemoral loss of contact area but No changes in peak pressures after meniscectomy in a lapine *in vivo* quadriceps force transfer model. Knee Surg. Sports Traumatol. Arthrosc. 23 (1), 65–73. 10.1007/s00167-014-3338-1 25274087

[B27] Lieber R. L. Boakes J. L. (1988). Sarcomere length and joint kinematics during torque production in frog hindlimb. Am. J. Physiology-Cell Physiology 254 (6), C759–C768. 10.1152/ajpcell.1988.254.6.C759 3259840

[B28] Lieber R. L. Shoemaker S. D. (1992). Muscle, joint, and tendon contributions to the torque profile of frog hip joint. Am. J. Physiology-Regulatory, Integr. Comp. Physiology 263 (3), R586–R590. 10.1152/ajpregu.1992.263.3.R586 1415645

[B29] Linn-Evans M. E. Petrucci M. N. Amundsen Huffmaster S. L. Chung J. W. Tuite P. J. Howell M. J. (2020). REM sleep without atonia is associated with increased rigidity in patients with mild to moderate Parkinson’s Disease. Clin. Neurophysiol. 131 (8), 2008–2016. 10.1016/j.clinph.2020.04.017 32451296 PMC7363578

[B30] Lorentzen J. Pradines M. Gracies J.-M. Nielsen J. B. (2018). On Denny-Brown’s ‘spastic dystonia’ - what is it and what causes it? Clin. Neurophysiology Official J. Int. Fed. Clin. Neurophysiology 129 (1), 89–94. 10.1016/j.clinph.2017.10.023 29161622

[B31] Love S. Gibson N. Smith N. Bear N. Blair E. the Australian Cerebral Palsy Register Group (2016). Interobserver reliability of the Australian spasticity assessment scale (ASAS). Dev. Med. and Child Neurology 58 (S2), 18–24. 10.1111/dmcn.13000 26762706

[B32] Luc Q. N. Querubin J. (2017). Clinical management of dystonia in childhood. Pediatr. Drugs 19 (5), 447–461. 10.1007/s40272-017-0243-3 28620849

[B33] Marsico P. Frontzek-Weps V. Balzer J. Van Hedel H. J. A. (2017). Hypertonia assessment tool: reliability and validity in children with neuromotor disorders. J. Child Neurology 32 (1), 132–138. 10.1177/0883073816671681 27742862

[B34] Mathis A. Mamidanna P. Cury K. M. Abe T. Murthy V. N. Mathis M. W. (2018). DeepLabCut: markerless pose estimation of user-defined body parts with deep learning. Nat. Neurosci. 21 (9), 1281–1289. 10.1038/s41593-018-0209-y 30127430

[B35] Mirbagheri M. M. Barbeau H. Kearney R. E. (2000). Intrinsic and reflex contributions to human ankle stiffness: variation with activation level and position. Exp. Brain Res. 135 (4), 423–436. 10.1007/s002210000534 11156307

[B36] Mirbagheri M. M. Settle K. Harvey R. Rymer W. Z. (2007). Neuromuscular abnormalities associated with spasticity of upper extremity muscles in hemiparetic stroke. J. Neurophysiology 98 (2), 629–637. 10.1152/JN.00049.2007 17537910

[B37] Monbaliu E. Ortibus E. De Cat J. Dan B. Heyrman L. Prinzie P. (2012). The Dyskinesia impairment scale: a new instrument to measure dystonia and choreoathetosis in dyskinetic cerebral palsy. Dev. Med. and Child Neurology 54 (3), 278–283. 10.1111/j.1469-8749.2011.04209.x 22428172

[B38] Nakazato K. Song H. Waga T. (2007). Dietary apple polyphenols enhance gastrocnemius function in wistar rats. Med. and Sci. Sports and Exerc. 39 (6), 934–940. 10.1249/mss.0b013e31803df4bc 17545882

[B39] Nielsen J. Kagamihara Y. (1993). Differential projection of the sural nerve to early and late recruited human tibialis anterior motor units: change of recruitment gain. Acta Physiol. Scand. 147 (4), 385–401. 10.1111/j.1748-1716.1993.tb09515.x 8493875

[B40] Nielsen J. Sinkjær T. Toft E. Kagamihara Y. (1994). Segmental reflexes and ankle joint stiffness during Co-contraction of antagonistic ankle muscles in man. Exp. Brain Res. 102 (2), 350–358. 10.1007/BF00227521 7705512

[B41] Nourizadeh M. Shadgan B. Abbasidezfouli S. Juricic M. Mulpuri K. (2024). Methods of muscle spasticity assessment in children with cerebral palsy: a scoping review. J. Orthop. Surg. Res. 19 (1), 401. 10.1186/s13018-024-04894-7 38992701 PMC11238363

[B42] Park G.-Y. Kwon D. R. (2011). Application of real-time sonoelastography in musculoskeletal diseases related to physical medicine and rehabilitation. Am. J. Phys. Med. and Rehabilitation 90 (11), 875–886. 10.1097/PHM.0b013e31821a6f8d 21552109

[B43] Patrick E. Ada L. (2006). The Tardieu scale differentiates contracture from spasticity whereas the Ashworth scale is confounded by it. Clin. Rehabil. 20 (2), 173–182. 10.1191/0269215506cr922oa 16541938

[B44] PennatiG. V. CarmentL. GodboltA. K. PlantinJ. BorgJ. LindbergP. G. (2023). Validity, intra-rater reliability and normative data of the Neuroflexor^TM^ device to measure spasticity of the ankle plantar flexors after stroke. J. Rehabilitation Med. 55 (March), jrm00356. 10.2340/jrm.v54.2067 36867093

[B45] Powers R. K. Campbell D. L. Rymer W. Z. (1989). Stretch reflex dynamics in spastic elbow flexor muscles. Ann. Neurology 25 (1), 32–42. 10.1002/ana.410250106 2913927

[B46] Powers R. K. Marder‐Meyer J. Rymer W. Z. (1988). Quantitative relations between hypertonia and stretch reflex threshold in spastic hemiparesis. Ann. Neurology 23 (2), 115–124. 10.1002/ana.410230203 3377434

[B47] Reedich E. J. Genry L. T. Steele P. R. Mena Avila E. Dowaliby L. Alexander D. (2023). Spinal motoneurons respond aberrantly to serotonin in a rabbit model of cerebral palsy. J. Physiology 601 (19), 4271–4289. 10.1113/JP284803 PMC10543617 37584461

[B48] Sanger T. D. Delgado M. R. Gaebler-Spira D. Hallett M. Mink J. W. Task Force on Childhood Motor Disorders (2003). Classification and definition of disorders causing hypertonia in childhood. Pediatrics 111 (1), e89–e97. 10.1542/peds.111.1.e89 12509602

[B49] Sinkjær T. Magnussen I. (1994). Passive, intrinsic and reflex-mediated stiffness in the ankle extensors of hemiparetic patients. Brain 117 (2), 355–363. 10.1093/brain/117.2.355 8186961

[B50] Sinkjær T. Toft E. Andreassen S. Hornemann B. C. (1988). Muscle stiffness in human ankle dorsiflexors: intrinsic and reflex components. J. Neurophysiology 60 (3), 1110–1121. 10.1152/jn.1988.60.3.1110 3171659

[B51] Sloot L. H. Weide G. Van Der Krogt M. M. Desloovere K. Harlaar J. Buizer A. I. (2021). Applying stretch to evoke hyperreflexia in spasticity testing: velocity vs. Acceleration. Front. Bioeng. Biotechnol. 8 (February), 591004. 10.3389/fbioe.2020.591004 33665186 PMC7921693

[B52] Stewart K. Lewis J. Wallen M. Bear N. Harvey A. (2021). The dyskinetic cerebral palsy functional impact scale: development and validation of a new tool. Dev. Med. and Child Neurology 63 (12), 1469–1475. 10.1111/dmcn.14960 34145577

[B53] Stikvoort García D. J. L. Sleutjes B. T. H. M. Mugge W. Plouvier J. J. Goedee H. S. Schouten A. C. (2024). Instrumented assessment of lower and upper motor neuron signs in amyotrophic lateral sclerosis using robotic manipulation: an explorative study. J. NeuroEngineering Rehabilitation 21 (1), 193. 10.1186/s12984-024-01485-9 PMC11520903 39472924

[B54] Synowiec S. Lu J. Yu L. Goussakov I. Lieber R. Drobyshevsky A. (2019). Spinal hyper-excitability and altered muscle structure contribute to muscle hypertonia in newborns after antenatal hypoxia-ischemia in a rabbit cerebral palsy model. Front. Neurology 9 (January), 1183. 10.3389/fneur.2018.01183 PMC6344443 30705663

[B55] Szopa A. Domagalska–Szopa M. Kidoń Z. Syczewska M. (2014). Quadriceps femoris spasticity in children with cerebral palsy: measurement with the pendulum test and relationship with gait abnormalities. J. NeuroEngineering Rehabilitation 11 (1), 166. 10.1186/1743-0003-11-166 PMC4277843 25516151

[B56] Tan S. Drobyshevsky A. Jilling T. Ji X. Ullman L. M. Englof I. (2005). Model of cerebral palsy in the perinatal rabbit. J. Child Neurology 20 (12), 972–979. 10.1177/08830738050200120801 16417845

[B57] Tarler M. D. Mortimer J. T. (2003). Comparison of joint torque evoked with monopolar and tripolar-cuff electrodes. IEEE Trans. Neural Syst. Rehabilitation Eng. 11 (3), 227–235. 10.1109/TNSRE.2003.816867 14518785

[B58] Toft E. Sinkjær T. Andreassen S. (1989). Mechanical and electromyographic responses to stretch of the human anterior tibial muscle at different levels of contraction. Exp. Brain Res. 74 (1), 213–219. 10.1007/BF00248294 2924837

[B59] Usuba M. Akai M. Shirasaki Y. Miyakawa S. (2007). Experimental joint contracture correction with low torque-long duration repeated stretching. Clin. Orthop. and Relat. Res. 456 (March), 70–78. 10.1097/BLO.0b013e31803212bf 17224840

[B60] Vallat R. (2018). Pingouin: statistics in Python. J. Open Source Softw. 3 (31), 1026. 10.21105/joss.01026

[B61] van den Noort J. C. Scholtes V. A. Harlaar J. (2009). Evaluation of clinical spasticity assessment in cerebral palsy using inertial sensors. Gait and Posture 30 (2), 138–143. 10.1016/j.gaitpost.2009.05.011 19525113

[B62] Willerslev-Olsen M. Lorentzen J. Sinkjær T. Nielsen J. B. O. (2013). Passive muscle properties are altered in children with cerebral palsy before the age of 3 Years and are difficult to distinguish clinically from spasticity. Dev. Med. and Child Neurology 55 (7), 617–623. 10.1111/dmcn.12124 23517272

[B63] Willerslev-Olsen M. Lund M. C. Lorentzen J. Barber L. Kofoed-Hansen M. Nielsen J. B. (2018). Impaired muscle growth precedes development of increased stiffness of the triceps surae musculotendinous unit in children with cerebral palsy. Dev. Med. Child Neurology 60 (7), 672–679. 10.1111/dmcn.13729 29573407

[B64] Wu Y.-N. Ren Y. Goldsmith A. Gaebler D. Liu S. Q. Zhang L.-Q. (2010). Characterization of spasticity in cerebral palsy: dependence of catch angle on velocity. Dev. Med. Child Neurology 52 (6), 563–569. 10.1111/j.1469-8749.2009.03602.x 20132137

