# Supplementary material for: A low-cost, open-source device to evaluate limb stiffness in a rabbit model of cerebral palsy
Source: Front Bioeng Biotechnol. 2025 Jun 5;13:1554775. doi: 10.3389/fbioe.2025.1554775 (PMC12177462; doi:10.3389/fbioe.2025.1554775)
Supplement: Supplementary file 1 [file Table1.docx]

| **Designator** | **Component** | **Number** | **Total cost** | **Source of materials** |
| --- | --- | --- | --- | --- |
| PLA 3D-printing filament | Prusament PLA Blend Royal Blue | 1 | $29.99 | https://www.prusa3d.com/en/product/prusament-pla-royal-blue-blend-970g/ |
| Arduino | Arduino Uno | 1 | $27.60 | https://store-usa.arduino.cc/collections/boards/products/arduino-uno-rev3 |
| Load cell | Sparkfun Mini Load Cell - 500g, Straight Bar (TAL221) | 1 | $10.95 | https://www.sparkfun.com/products/14728 |
| Load cell amplifier | Sparkfun HX711 | 1 | $10.95 | https://www.sparkfun.com/products/13879 |
| 16ch PWM servo driver | Adafruit PCA9685 | 1 | $14.95 | https://www.adafruit.com/product/815 |
| Momentary switch | Digi-Key EG1900-ND | 1 | $2.39 | https://www.digikey.com/en/products/detail/e-switch/RP3502ABLK/280446 |
| Servo motor | Adafruit Micro servo | 1 | $5.95 | https://www.adafruit.com/product/169 |
| Ball bearings | McMaster-Carr 6455K27 | 1 | $6.95 | https://www.mcmaster.com/6455K27/ |
| Neodymium magnets | McMaster-Carr 5862K141 | 20 | $13.00 | https://www.mcmaster.com/5862K141/ |
| Rubber feet | McMaster-Carr 9309K79 | 4 | $3.72 | https://www.mcmaster.com/9309K79/ |
| Screws for securing the servo motor to the base plate | McMaster-Carr 91292A312 | 2 | $0.82 | https://www.mcmaster.com/91292A312/ |
| Screws for securing the load cell | McMaster-Carr 92095A187 | 4 | $0.64 | https://www.mcmaster.com/92095A187/ |
| Nuts for securing the load cell | McMaster-Carr 91828A211 | 4 | $0.20 | https://www.mcmaster.com/91828A211/ |
| Nylon Standoffs | Litorange 320 PCS M2.5 Male Female Nylon Hex Spacer Standoff | 12 | $0.50 | https://www.amazon.com/gp/product/B08HS7694T |
| Solid core wire |  |  |  |  |
| Foam | Polyurethane Foam Sheet | 1 | $5 | https://www.amazon.com/DECOHS-Polyurethane-Sheet-16x12x1-Cuttable-Cases-Packing/dp/B0C3VMKWXF |
| Cyanoacrylate glue | Loctite 1364076 | 1 | $3.64 | https://www.amazon.com/Loctite-1364076-Super-Squeeze-Liquid/dp/B0006HUJCQ |
| Computer |  |  |  | A computer is required for data acquisition. The software is python-based and cross platform. Any computer with a USB port and capable of running python 3.9 and PyQt5 can be used. We have tested computers running Windows 10, Windows 11, and Linux Mint 21.3 Cinnamon. |

**Supplemental Table 1: Bill of materials**
