# Supplementary material for: A low-cost, open-source device to evaluate limb stiffness in a rabbit model of cerebral palsy
Source: Front Bioeng Biotechnol. 2025 Jun 5;13:1554775. doi: 10.3389/fbioe.2025.1554775 (PMC12177462; doi:10.3389/fbioe.2025.1554775)
Supplement: Supplementary file 2 [file DataSheet1.zip › MarinManuel-TorqueMeter-772995c/Assets/Datasheets/16-channel-pwm-servo-driver.pdf]

Adafruit PCA9685 16-Channel Servo Driver

Created by Bill Earl

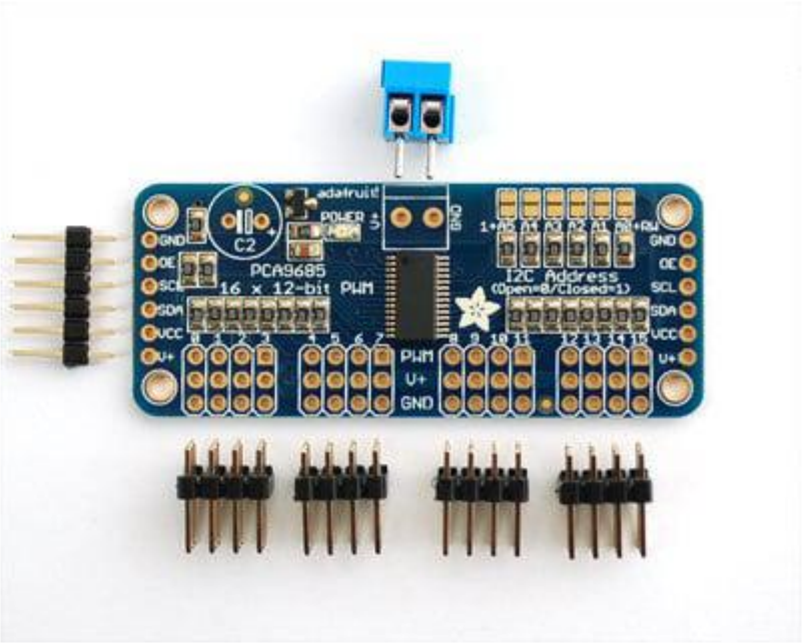

https://learn.adafruit.com/16-channel-pwm-servo-driver

Last updated on 2021-11-15 05:52:14 PM EST

Table of Contents

Overview 5

Pinouts 6
• Power Pins 6
• Control Pins 6
• Output Ports 7

Assembly 7
• Install the Servo Headers 7
• Solder all pins 7
• Add Headers for Control 8
• Install Power Terminals 8

Hooking it Up 8
• Connecting to the Arduino 8
• Power for the Servos 10
• Adding a Capacitor to the thru-hole capacitor slot 10
• Connecting a Servo 11
• Adding More Servos 11

Chaining Drivers 12
• Addressing the Boards 12

Using the Adafruit Library 14
• Install Adafruit PCA9685 library 14
• Test with the Example Code: 14
• Connect a Servo 15
• Calibrating your Servos 16
• Converting from Degrees to Pulse Length 16

Library Reference 16
• setPWMFreq(freq) 16
• Description 16
• setPWM(channel, on, off) 17
• Using as GPIO 18

Arduino Library Docs 18

Python & CircuitPython 18
• CircuitPython Microcontroller Wiring 18
• Python Computer Wiring 19
• CircuitPython Installation of PCA9685 and ServoKit Libraries 20
• Python Installation of PCA9685 and ServoKit Libraries 21
• CircuitPython & Python Usage 21
• Dimming LEDs 22
• Full Example Code 24
• Controlling Servos 24
• Standard Servos 25
• Continuous Rotation Servos 27
• Full Example Code 28

Python Docs 28

Python Docs: ServoKit 28

Downloads 28

- Files 28
- Schematic & Fabrication Print 29

FAQ 29

Overview

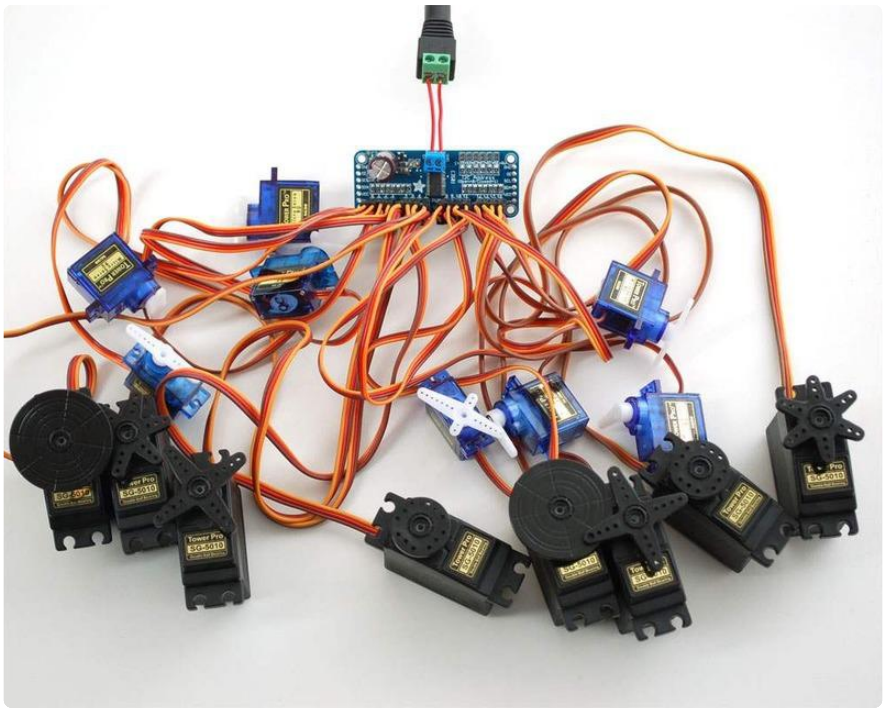

Driving servo motors with the Arduino Servo library is pretty easy, but each one consumes a precious pin - not to mention some Arduino processing power. The Adafruit 16-Channel 12-bit PWM/Servo Driver will drive up to 16 servos over I2C with only 2 pins. The on-board PWM controller will drive all 16 channels simultaneously with no additional Arduino processing overhead. What's more, you can chain up to 62 of them to control up to 992 servos - all with the same 2 pins!

The Adafruit PWM/Servo Driver is the perfect solution for any project that requires a lot of servos.

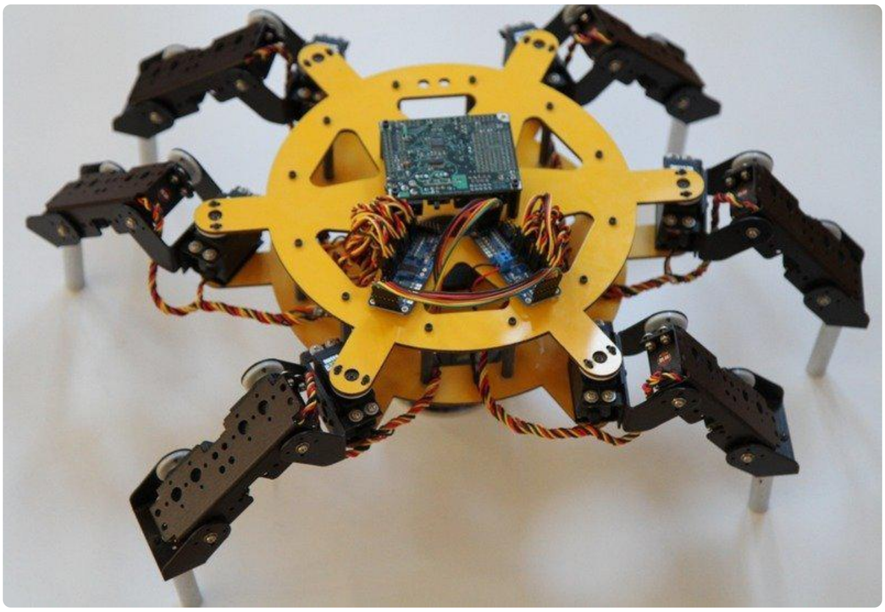

Pinouts

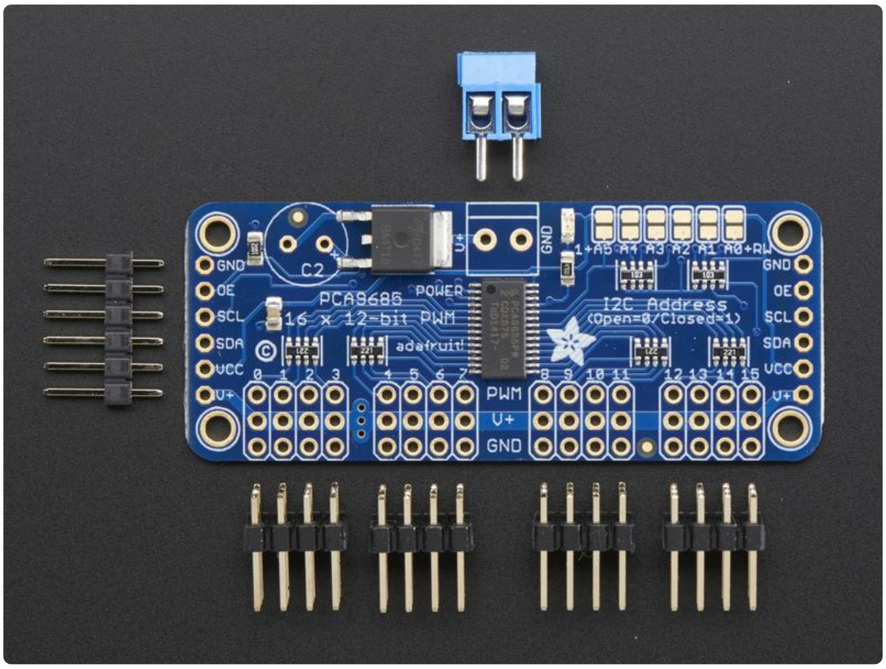

There are two sets of control input pins on either side. Both sides of the pins are identical! Use whichever side you like, you can also easily chain by connecting up two side-by-side

Power Pins

- GND - This is the power and signal ground pin, must be connected
- VCC - This is the logic power pin, connect this to the logic level you want to use for the PCA9685 output, should be 3 - 5V max! It's also used for the 10K pullups on SCL/SDA so unless you have your own pullups, have it match the microcontroller's logic level too!
- V+ - This is an optional power pin that will supply distributed power to the servos. If you are not using for servos you can leave disconnected. It is not used at all by the chip. You can also inject power from the 2-pin terminal block at the top of the board. You should provide 5-6VDC if you are using servos. If you have to, you can go higher to 12VDC, but if you mess up and connect VCC to V+ you could damage your board!

Control Pins

- SCL - I2C clock pin, connect to your microcontrollers I2C clock line. Can use 3V or 5V logic, and has a weak pullup to VCC

- SDA - I2C data pin, connect to your microcontrollers I2C data line. Can use 3V or 5V logic, and has a weak pullup to VCC
- OE - Output enable. Can be used to quickly disable all outputs. When this pin is l ow all pins are enabled. When the pin is high the outputs are disabled. Pulled low by default so it's an optional pin!

Output Ports

There are 16 output ports. Each port has 3 pins: V+, GND and the PWM output. Each PWM runs completely independently but they must all have the same PWM frequency. That is, for LEDs you probably want 1.0 KHz but servos need 60 Hz - so you cannot use half for LEDs @ 1.0 KHz and half @ 60 Hz.

They're set up for servos but you can use them for LEDs! Max current per pin is 25mA.

There are 220 ohm resistors in series with all PWM Pins and the output logic is the same as VCC so keep that in mind if using LEDs.

Assembly

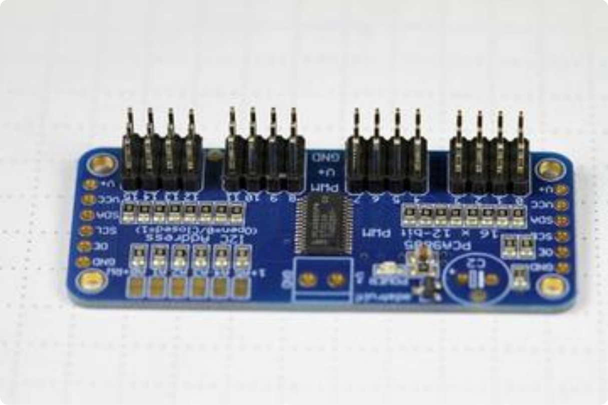

Install the Servo Headers

Install 4 3x4 pin male headers into the marked positions along the edge of the board.

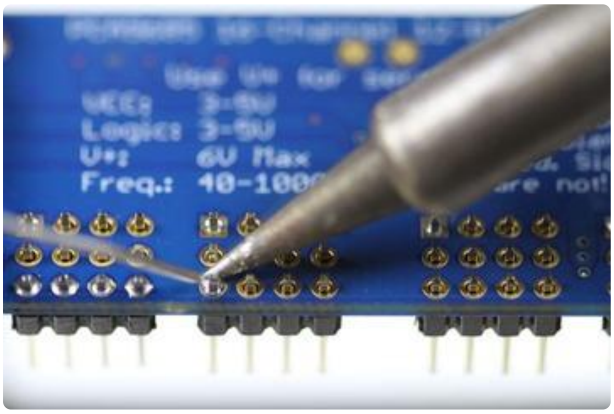

Solder all pins

There are a lot of them!

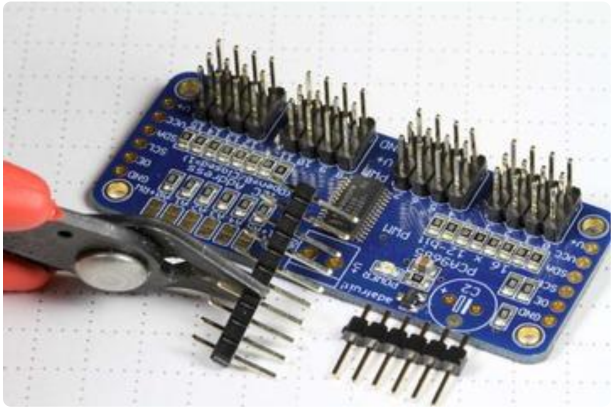

Add Headers for Control

A strip of male header is included. Where you want to install headers and on what side depends a little on use:

- For breadboard (http://adafru.it/239) use, install headers on the bottom of the board.
- For use with jumper wires (http://adafru.it/758), install the headers on top of the board.
- For use with our 6-pin cable (http://adafru.it/206), install the headers on top of the board.

If you are chaining multiple driver boards, you will want headers on both ends.

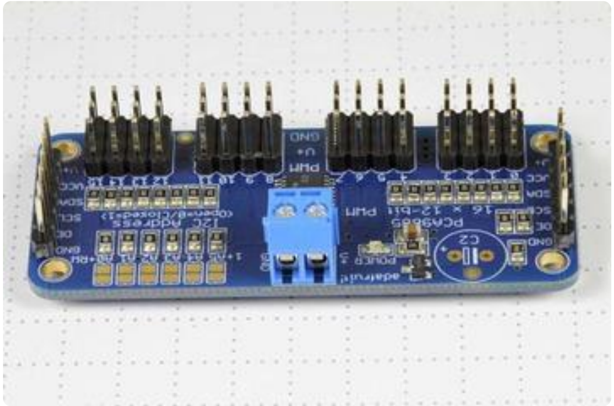

Install Power Terminals

If you are chaining multiple driver boards, you only need a power terminal on the first one.

Hooking it Up

Connecting to the Arduino

The PWM/Servo Driver uses I2C so it take only 4 wires to connect to your Arduino:

"Classic" Arduino wiring:

- +5v -> VCC (this is power for the BREAKOUT only, NOT the servo power!)
- GND -> GND
- Analog 4 -> SDA
- Analog 5 -> SCL

Older Mega wiring:

- +5v -> VCC (this is power for the BREAKOUT only, NOT the servo power!)
- GND -> GND
- Digital 20 -> SDA
- Digital 21 -> SCL

R3 and later Arduino wiring (Uno, Mega & Leonardo):
(These boards have dedicated SDA & SCL pins on the header nearest the USB connector)

- +5v -> VCC (this is power for the BREAKOUT only, NOT the servo power!)
- GND -> GND
- SDA -> SDA
- SCL -> SCL

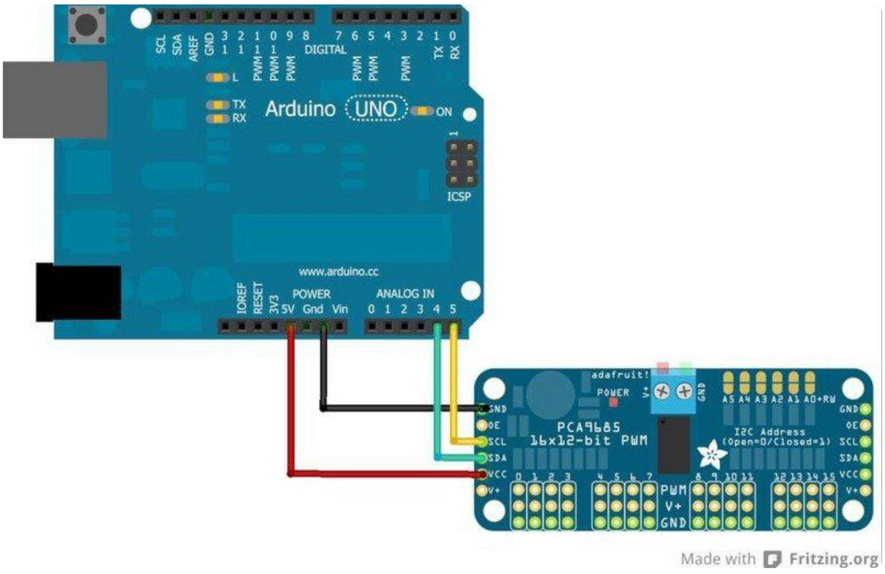

The VCC pin is just power for the chip itself. If you want to connect servos or LEDs that use the V+ pins, you MUST connect the V+ pin as well. The V+ pin can be as high as 6V even if VCC is 3.3V (the chip is 5V safe). We suggest connecting power through the blue terminal block since it is polarity protected.

Power for the Servos

Most servos are designed to run on about 5 or 6v. Keep in mind that a lot of servos moving at the same time (particularly large powerful ones) will need a lot of current. Even micro servos will draw several hundred mA when moving. Some High-torque servos will draw more than 1A each under load.

Good power choices are:

- 5v 2A switching power supply (http://adafru.it/276)
- 5v 10A switching power supply (http://adafru.it/658)
- 4xAA Battery Holder (http://adafru.it/830) - 6v with Alkaline cells. 4.8v with NiMH rechargeable cells.
- 4.8 or 6v Rechargeable RC battery packs from a hobby store.

It is not a good idea to use the Arduino 5v pin to power your servos. Electrical noise and 'brownouts' from excess current draw can cause your Arduino to act erratically, reset and/or overheat.

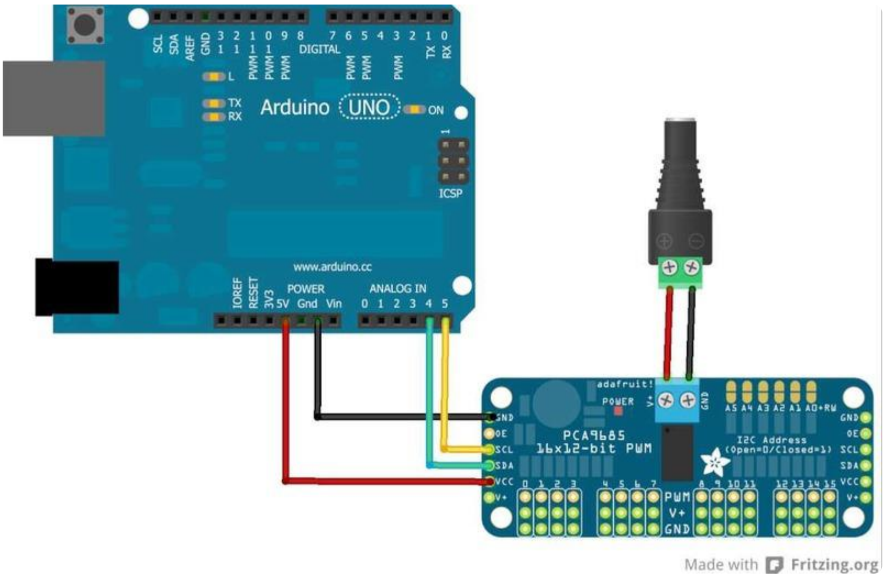

Adding a Capacitor to the thru-hole capacitor slot

We have a spot on the PCB for soldering in an electrolytic capacitor. Based on your usage, you may or may not need a capacitor. If you are driving a lot of servos from a power supply that dips a lot when the servos move, n * 100uF where n is the number of servos is a good place to start - eg 470uF or more for 5 servos. Since its so dependent on servo current draw, the torque on each motor, and what power supply, there is no "one magic capacitor value" we can suggest which is why we don't include

a capacitor in the kit.

Connecting a Servo

Most servos come with a standard 3-pin female connector that will plug directly into the headers on the Servo Driver. Be sure to align the plug with the ground wire (usually black or brown) with the bottom row and the signal wire (usually yellow or white) on the top.

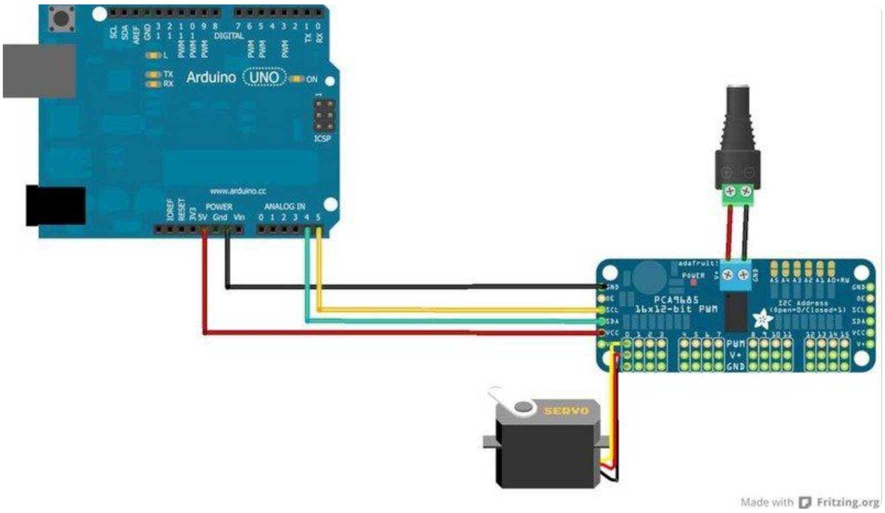

Adding More Servos

Up to 16 servos can be attached to one board. If you need to control more than 16 servos, additional boards can be chained as described on the next page.

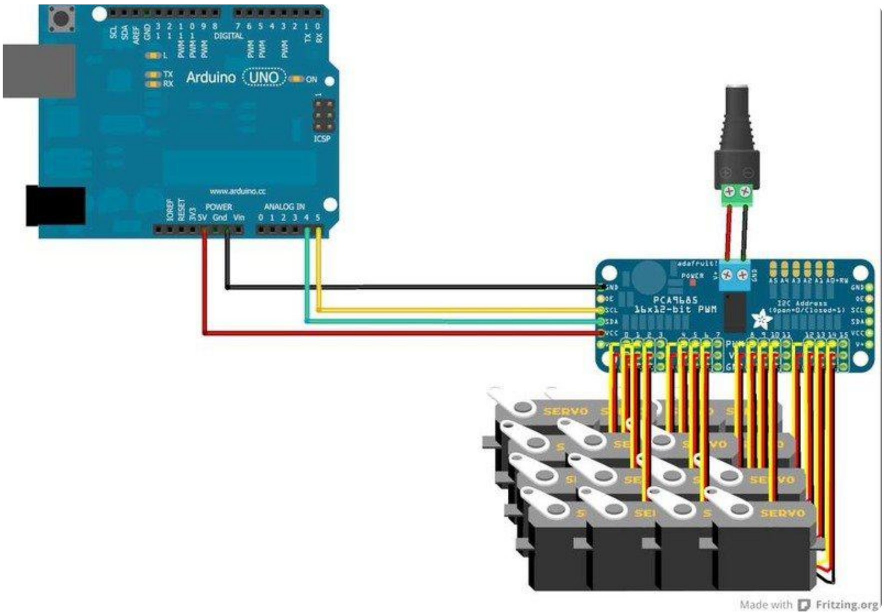

Chaining Drivers

Multiple Drivers (up to 62) can be chained to control still more servos. With headers at both ends of the board, the wiring is as simple as connecting a 6-pin parallel cable (http://adafru.it/206) from one board to the next.

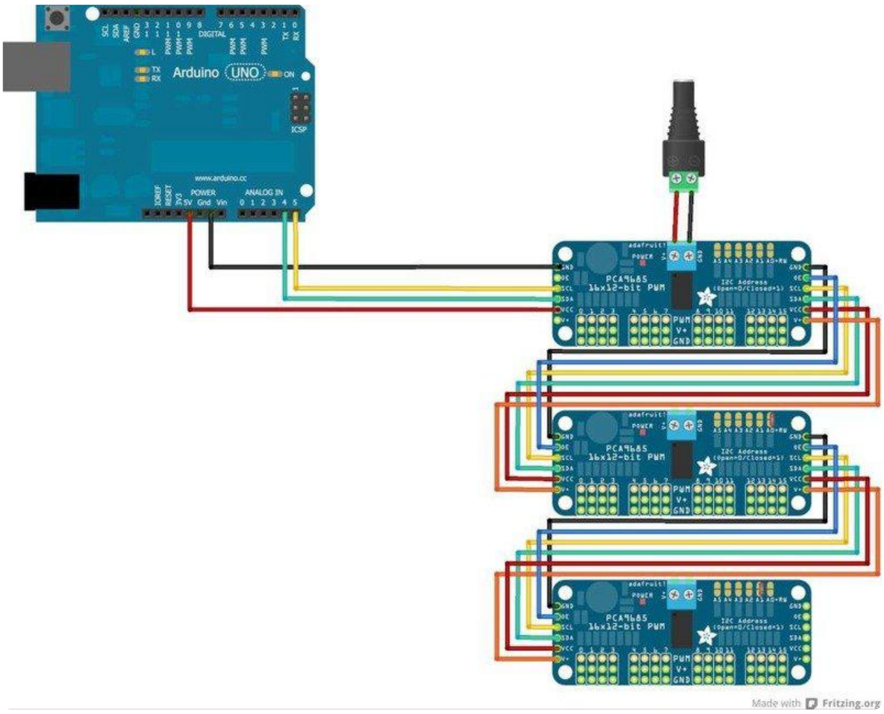

Addressing the Boards

Each board in the chain must be assigned a unique address. This is done with the address jumpers on the upper right edge of the board. The I2C base address for each board is 0x40. The binary address that you program with the address jumpers is added to the base I2C address.

To program the address offset, use a drop of solder to bridge the corresponding address jumper for each binary '1' in the address.

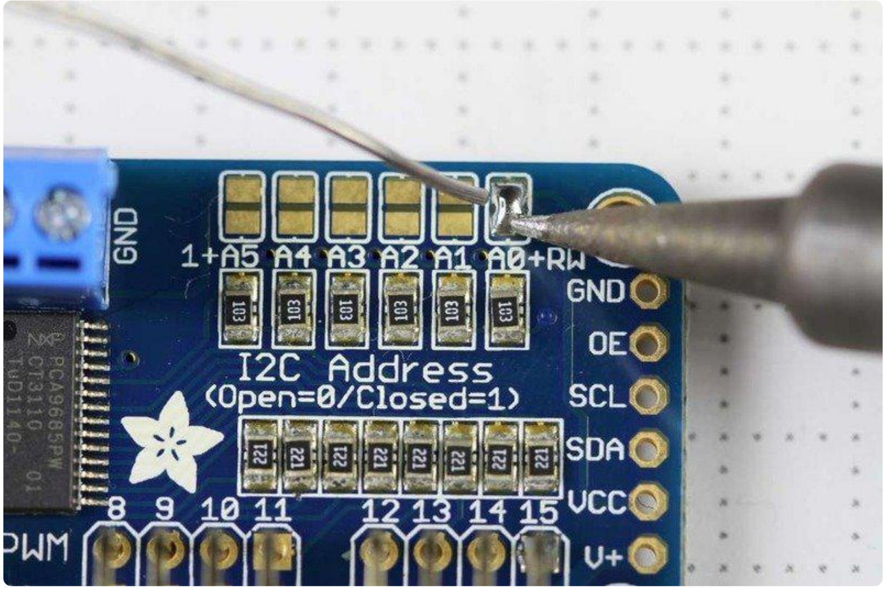

Board 0: Address = 0x40 Offset = binary 00000 (no jumpers required)
Board 1: Address = 0x41 Offset = binary 00001 (bridge A0 as in the photo above)
Board 2: Address = 0x42 Offset = binary 00010 (bridge A1)
Board 3: Address = 0x43 Offset = binary 00011 (bridge A0 & A1)
Board 4: Address = 0x44 Offset = binary 00100 (bridge A2)

etc.

In your sketch, you'll need to declare a separate pobject for each board. Call begin on each object, and control each servo through the object it's attached to. For example:

```
#include &lt;Wire.h&gt;
#include &lt;Adafruit_PWMServoDriver.h&gt;

Adafruit_PWMServoDriver pwm1 = Adafruit_PWMServoDriver(0x40);
Adafruit_PWMServoDriver pwm2 = Adafruit_PWMServoDriver(0x41);

void setup() {
  Serial.begin(9600);
  Serial.println("16 channel PWM test!");

  pwm1.begin();
  pwm1.setPWMFreq(1600);  // This is the maximum PWM frequency

  pwm2.begin();
  pwm2.setPWMFreq(1600);  // This is the maximum PWM frequency
}
```

Using the Adafruit Library

Since the PWM Servo Driver is controlled over I2C, its super easy to use with any microcontroller or microcomputer. In this demo we'll show using it with the Arduino IDE but the C++ code can be ported easily

Install Adafruit PCA9685 library

To begin reading sensor data, you will need to install the Adafruit_PWMServo library (code on our github repository) (https://adafru.it/aQl). It is available from the Arduino library manager so we recommend using that.

From the IDE open up the library manager...

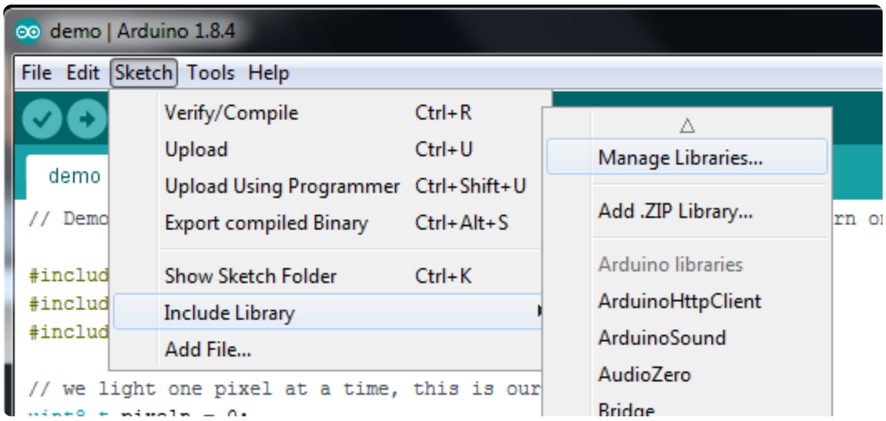

And type in adafruit pwm to locate the library. Click Install

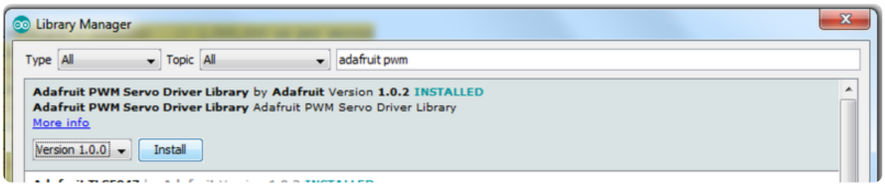

We also have a great tutorial on Arduino library installation at: http://learn.adafruit.com/adafruit-all-about-arduino-libraries-install-use (https://adafru.it/aYM)

Test with the Example Code:

First make sure all copies of the Arduino IDE are closed.

Next open the Arduino IDE and select File->Examples->Adafruit_PWMServoDriver->Servo. This will open the example file in an IDE window.

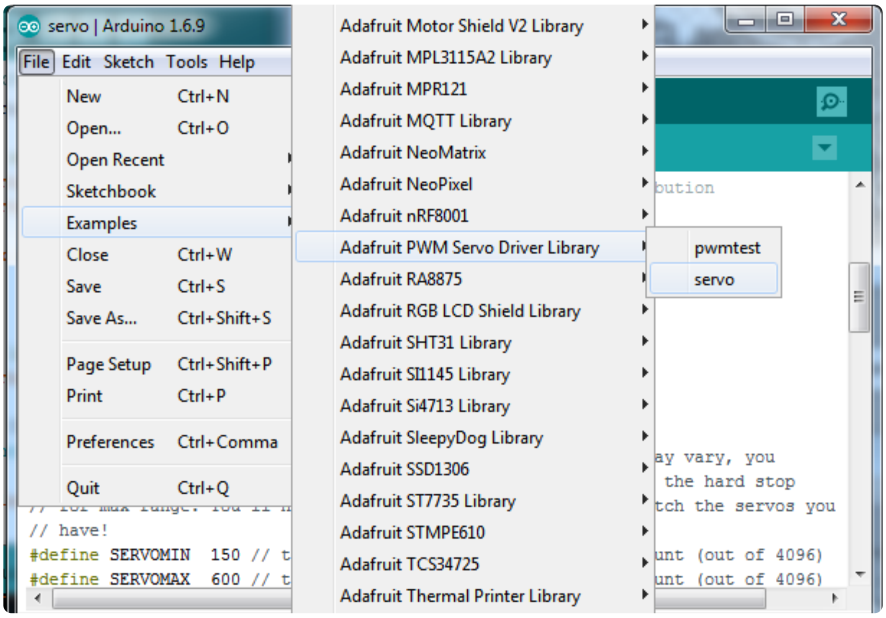

If using a Breakout:

Connect the driver board and servo as shown on the previous page. Don't forget to provide power to both Vin (3-5V logic level) and V+ (5V servo power). Check the green LED is lit!

If using a Shield:

Plug the shield into your Arduino. Don't forget you will also have to provide 5V to the V+ terminal block. Both red and green LEDs must be lit.

If using a FeatherWing:

Plug the FeatherWing into your Feather. Don't forget you will also have to provide 5V to the V+ terminal block. Check the green LED is lit!

Connect a Servo

A single servo should be plugged into the PWM #0 port, the first port. You should see the servo sweep back and forth over approximately 180 degrees.

Calibrating your Servos

Servo pulse timing varies between different brands and models. Since it is an analog control circuit, there is often some variation between samples of the same brand and model. For precise position control, you will want to calibrate the minumum and maximum pulse-widths in your code to match known positions of the servo.

Find the Minimum:
Using the example code, edit SERVOMIN until the low-point of the sweep reaches the minimum range of travel. It is best to approach this gradually and stop before the physical limit of travel is reached.

Find the Maximum:
Again using the example code, edit SERVOMAX until the high-point of the sweep reaches the maximum range of travel. Again, is best to approach this gradually and stop before the physical limit of travel is reached.

Use caution when adjusting SERVOMIN and SERVOMAX. Hitting the physical limits of travel can strip the gears and permanently damage your servo.

Converting from Degrees to Pulse Length

The Arduino "map()" function (https://adafru.it/aQm) is an easy way to convert between degrees of rotation and your calibrated SERVOMIN and SERVOMAX pulse lengths. Assuming a typical servo with 180 degrees of rotation; once you have calibrated SERVOMIN to the 0-degree position and SERVOMAX to the 180 degree position, you can convert any angle between 0 and 180 degrees to the corresponding pulse length with the following line of code:

```
pulselength = map(degrees, 0, 180, SERVOMIN, SERVOMAX);
```

Library Reference

setPWMFreq(freq)

Description

This function can be used to adjust the PWM frequency, which determines how many full 'pulses' per second are generated by the IC. Stated differently, the frequency

determines how 'long' each pulse is in duration from start to finish, taking into account both the high and low segments of the pulse.

Frequency is important in PWM, since setting the frequency too high with a very small duty cycle can cause problems, since the 'rise time' of the signal (the time it takes to go from 0V to VCC) may be longer than the time the signal is active, and the PWM output will appear smoothed out and may not even reach VCC, potentially causing a number of problems.

Arguments

- freq: A number representing the frequency in Hz, between 40 and 1600

Example

The following code will set the PWM frequency to 1000Hz:

```
pwm.setPWMFreq(1000)
```

setPWM(channel, on, off)

Description

This function sets the start (on) and end (off) of the high segment of the PWM pulse on a specific channel. You specify the 'tick' value between 0..4095 when the signal will turn on, and when it will turn off. Channel indicates which of the 16 PWM outputs should be updated with the new values.

Arguments

- channel: The channel that should be updated with the new values (0..15)
- on: The tick (between 0..4095) when the signal should transition from low to high
- off:the tick (between 0..4095) when the signal should transition from high to low

Example

The following example will cause channel 15 to start low, go high around 25% into the pulse (tick 1024 out of 4096), transition back to low 75% into the pulse (tick 3072), and remain low for the last 25% of the pulse:

```
pwm.setPWM(15, 1024, 3072)
```

Using as GPIO

There's also some special settings for turning the pins fully on or fully off

You can set the pin to be fully on with

`pwm.setPWM(pin, 4096, 0);`

You can set the pin to be fully off with

`pwm.setPWM(pin, 0, 4096);`

Arduino Library Docs

Arduino Library Docs (https://adafru.it/Au7)

Python & CircuitPython

It's easy to use the PCA9685 sensor with Python or CircuitPython and the Adafruit CircuitPython PCA9685 (https://adafru.it/tZF) module. This module allows you to easily write Python code that control servos and PWM with this breakout.

You can use this sensor with any CircuitPython microcontroller board or with a computer that has GPIO and Python thanks to Adafruit_Blinka, our CircuitPython-for-Python compatibility library (https://adafru.it/BSN).

CircuitPython Microcontroller Wiring

First wire up a PCA9685 to your board exactly as shown on the previous pages for Arduino. Here's an example of wiring a Feather M0 to the sensor with I2C:

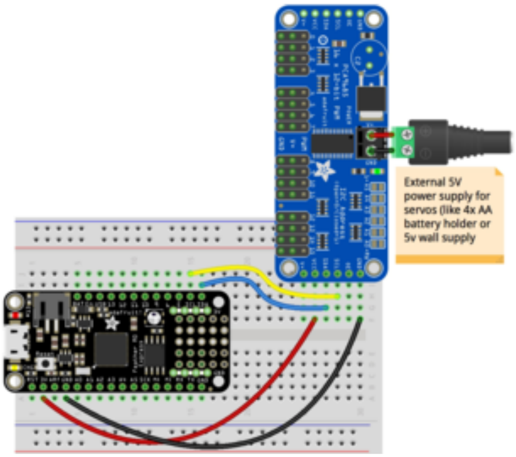

- Board 3V to sensor VCC
- Board GND to sensor GND
- Board SCL to sensor SCL
- Board SDA to sensor SDA

Python Computer Wiring

Since there's dozens of Linux computers/boards you can use we will show wiring for Raspberry Pi. For other platforms, please visit the guide for CircuitPython on Linux to see whether your platform is supported (https://adafru.it/BSN).

Here's the Raspberry Pi wired with I2C:

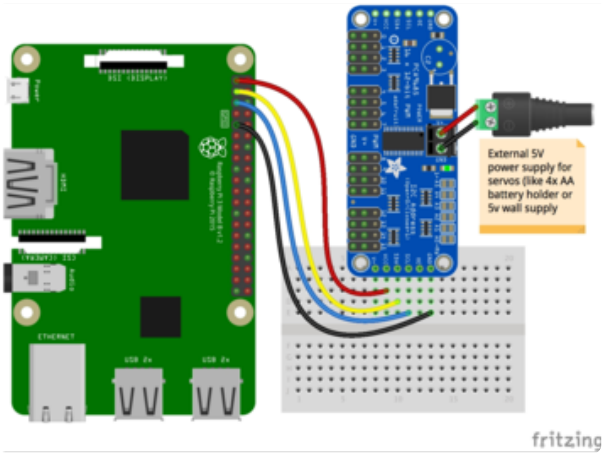

- Pi 3V3 to sensor VCC
- Pi GND to sensor GND
- Pi SCL to sensor SCL
- Pi SDA to sensor SDA

Don't try to power your servos from the RasPi or Linux board's 5V power, you can easily cause a power supply brown-out and mess up your Pi! Use a separate 5v 2A or 4A adapter

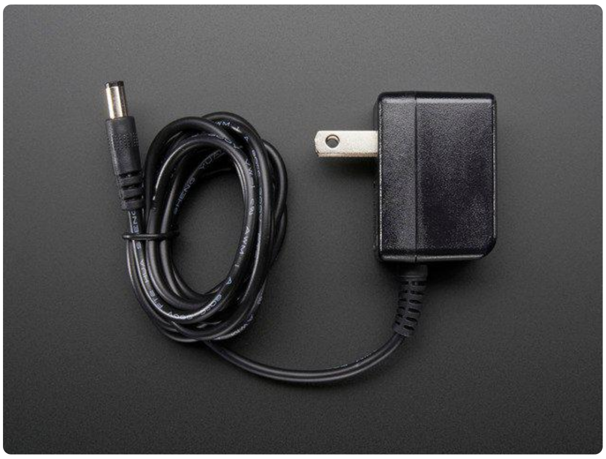

5V 2A (2000mA) switching power supply - UL Listed
This is an FCC/CE certified and UL listed power supply. Need a lot of 5V power? This switching supply gives a clean regulated 5V output at up to 2000mA. 110 or 240 input, so it works...
https://www.adafruit.com/product/276

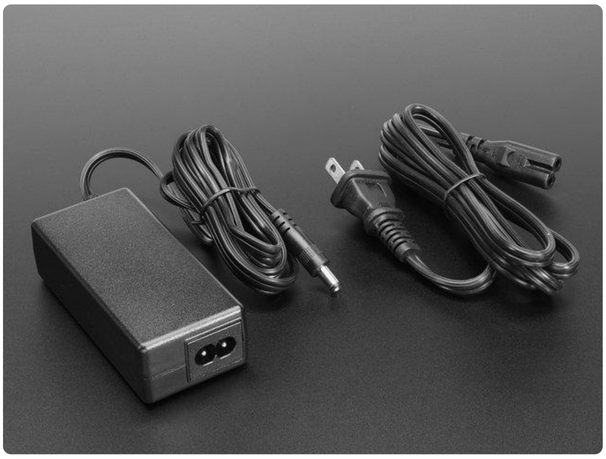

5V 4A (4000mA) switching power supply - UL Listed
Need a lot of 5V power? This switching supply gives a clean regulated 5V output at up to 4 Amps (4000mA). 110 or 240 input, so it works in any country. The plugs are "US...
https://www.adafruit.com/product/1466

CircuitPython Installation of PCA9685 and ServoKit Libraries

You'll need to install the Adafruit CircuitPython PCA9685 (https://adafru.it/tZF) library on your CircuitPython board.

First make sure you are running the latest version of Adafruit CircuitPython (https://adafru.it/Amd) for your board.

Next you'll need to install the necessary libraries to use the hardware--carefully follow the steps to find and install these libraries from Adafruit's CircuitPython library bundle (https://adafru.it/uap). Our CircuitPython starter guide has a great page on how to install the library bundle (https://adafru.it/ABU).

For non-express boards like the Trinket M0 or Gemma M0, you'll need to manually install the necessary libraries from the bundle:

- adafruit_pca9685.mpy
- adafruit_bus_device

- adafruit_register
- adafruit_motor
- adafruit_servokit

Before continuing make sure your board's lib folder or root filesystem has the adafruit_pca9685.mpy, adafruit_register, adafruit_servokit, adafruit_motor and adafruit_bus_device files and folders copied over.

Next connect to the board's serial REPL (https://adafru.it/Awz) so you are at the CircuitPython >>> prompt.

Python Installation of PCA9685 and ServoKit Libraries

You'll need to install the Adafruit_Blinka library that provides the CircuitPython support in Python. This may also require enabling I2C on your platform and verifying you are running Python 3. Since each platform is a little different, and Linux changes often, please visit the CircuitPython on Linux guide to get your computer ready (https://adafru.it/BSN)!

Once that's done, from your command line run the following commands:

- `sudo pip3 install adafruit-circuitpython-pca9685`
- `sudo pip3 install adafruit-circuitpython-servokit`

If your default Python is version 3 you may need to run 'pip' instead. Just make sure you aren't trying to use CircuitPython on Python 2.x, it isn't supported!

CircuitPython & Python Usage

The following section will show how to control the PCA9685 from the board's Python prompt / REPL. You'll learn how to interactively control servos and dim LEDs by typing in the code below.

Dimming LEDs

Run the following code to import the necessary modules and initialize the I2C connection with the sensor:

```
import board
import busio
import adafruit_pca9685
i2c = busio.I2C(board.SCL, board.SDA)
pca = adafruit_pca9685.PCA9685(i2c)
```

Each channel of the PCA9685 can be used to control the brightness of an LED. The PCA9685 generates a high-speed PWM signal which turns the LED on and off very quickly. If the LED is turned on longer than turned off it will appear brighter to your eyes.

First wire a LED to the board as follows. Note you don't need to use a resistor to limit current through the LED as the PCA9685 will limit the current to around 10mA:

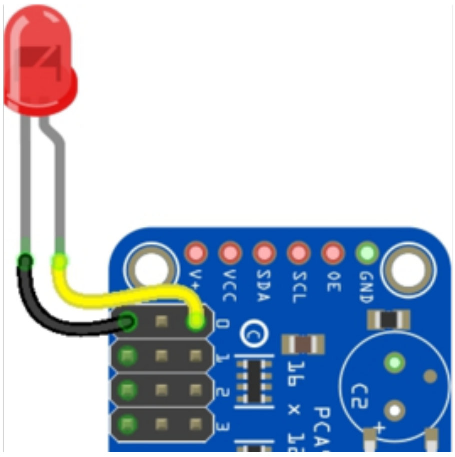

- LED cathode / shorter leg to PCA9685 channel GND / ground.
- LED anode / longer leg to PCA9685 channel PWM.

The PCA9685 class provides control of the PWM frequency and each channel's duty cycle. Check out the PCA9685 class documentation (https://adafru.it/C5n) for more details.

For dimming LEDs you typically don't need to use a fast PWM signal frequency and can set the board's PWM frequency to 60hz by setting the frequency attribute:

```
pca.frequency = 60
```

The PCA9685 supports 16 separate channels that share a frequency but can have independent duty cycles. That way you could dim 16 LEDs separately!

The PCA9685 object has a channels attribute which has an object for each channel that can control the duty cycle. To get the individual channel use the [] to index into ch annels.

```
led_channel = pca.channels[0]
```

Now control the LED brightness by controlling the duty cycle of the channel connected to the LED. The duty cycle value should be a 16-bit value, i.e. 0 to 0xffff, which represents what percent of the time the signal is on vs. off. A value of 0xffff is 100% brightness, 0 is 0% brightness, and in-between values go from 0% to 100% brightness.

For example set the LED completely on with a duty cycle of 0xffff:

```
led_channel.duty_cycle = 0xffff
```

After running the command above you should see the LED light up at full brightness!

Now turn the LED off with a duty cycle of 0:

```
led_channel.duty_cycle = 0
```

Try an in-between value like 1000:

```
led_channel.duty_cycle = 1000
```

You should see the LED dimly lit. Try experimenting with other duty cycle values to see how the LED changes brightness!

For example make the LED glow on and off by setting duty_cycle in a loop:

```
# Increase brightness:
for i in range(0xffff):
    led_channel.duty_cycle = i

# Decrease brightness:
for i in range(0xffff, 0, -1):
    led_channel.duty_cycle = i
```

These for loops take a while because 16-bits is a lot of numbers. CTRL-C to stop the loop from running and return to the REPL.

Full Example Code

```
# SPDX-FileCopyrightText: 2021 ladyada for Adafruit Industries
# SPDX-License-Identifier: MIT

# This simple test outputs a 50% duty cycle PWM single on the 0th channel. Connect
an LED and
# resistor in series to the pin to visualize duty cycle changes and its impact on
brightness.

from board import SCL, SDA
import busio

# Import the PCA9685 module.
from adafruit_pca9685 import PCA9685

# Create the I2C bus interface.
i2c_bus = busio.I2C(SCL, SDA)

# Create a simple PCA9685 class instance.
pca = PCA9685(i2c_bus)

# Set the PWM frequency to 60hz.
pca.frequency = 60

# Set the PWM duty cycle for channel zero to 50%. duty_cycle is 16 bits to match
other PWM objects
# but the PCA9685 will only actually give 12 bits of resolution.
pca.channels[0].duty_cycle = 0x7FFF
```

Controlling Servos

We've written a handy CircuitPython library for the various PWM/Servo kits called Adafruit CircuitPython ServoKit (https://adafru.it/Dpu) that handles all the complicated setup for you. All you need to do is import the appropriate class from the library, and then all the features of that class are available for use. We're going to show you how to import the `ServoKit` class and use it to control servo motors with the Adafruit 16-channel breakout.

If you aren't familiar with servos be sure to first read this intro to servos page (https://adafru.it/scW) and this in-depth servo guide page (https://adafru.it/scS).

First connect the servo to channel 0 on the PCA9685. Here is an example of a servo connected to channel 0:

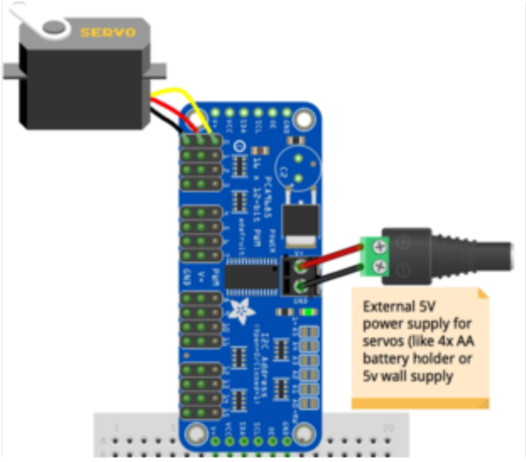

- Servo orange wire to breakout PWM on channel 0
- Servo red wire to breakout V+ on channel 0
- Servo brown wire to breakout Gnd on channel 0

Check your servo data sheet to verify how to connect it!

Be sure you've turned on or plugged in the external 5V power supply to the PCA9685 board too!

First you'll need to import and initialize the `ServoKit` class. You must specify the number of channels available on your board. The breakout has 16 channels, so when you create the class object, you will specify `16`.

```
from adafruit_servokit import ServoKit
kit = ServoKit(channels=16)
```

Now you're ready to control both standard and continuous rotation servos.

Standard Servos

To control a standard servo, you need to specify the channel the servo is connected to. You can then control movement by setting `angle` to the number of degrees.

For example to move the servo connected to channel `0` to `180` degrees:

```
pca.frequency = 50
```

Now that the PCA9685 is set up for servos lets make a Servo object so that we can adjust the servo based on angle instead of duty_cycle.

By default the Servo class will use actuation range, minimum pulse-width, and maximum pulse-width values that should work for most servos. However check the

Servo class documentation (https://adafru.it/BNE) for more details on extra parameters to customize the signal generated for your servos.

```
import adafruit_motor.servo
servo = adafruit_motor.servo.Servo(servo_channel)
```

With Servo, you specify a position as an angle. The angle will always be between 0 and the actuation range given when Servo was created. The default is 180 degrees but your servo might have a smaller sweep--change the total angle by specifying the actuation_angle parameter in the Servo class initializer above.

Now set the angle to 180, one extreme of the range:

```
    kit.servo[0].angle = 180
```

To return the servo to `0` degrees:

```
kit.servo[0].angle = 0
```

With a standard servo, you specify the position as an angle. The angle will always be between 0 and the actuation range. The default is 180 degrees but your servo may have a smaller sweep. You can change the total angle by setting `actuation_range`.

For example, to set the actuation range to 160 degrees:

```
servokit.servo[0].actuation_range = 160
```

Often the range an individual servo recognises varies a bit from other servos. If the servo didn't sweep the full expected range, then try adjusting the minimum and maximum pulse widths using `set_pulse_width_range(min_pulse, max_pulse)`.

To set the pulse width range to a minimum of 1000 and a maximum of 2000:

```
kit.servo[0].set_pulse_width_range(1000, 2000)
```

That's all there is to controlling standard servos with the PCA9685 breakout, Python and `ServoKit`!

Continuous Rotation Servos

To control a continuous rotation servo, you must specify the channel the servo is on. Then you can control movement using `throttle`.

For example, to start the continuous rotation servo connected to channel `1` to full throttle forwards:

```
kit.continuous_servo[1].throttle = 1
```

To start the continuous rotation servo connected to channel `1` to full reverse throttle:

```
kit.continuous_servo[1].throttle = -1
```

To set half throttle, use a decimal:

```
kit.continuous_servo[1].throttle = 0.5
```

And, to stop continuous rotation servo movement set `throttle` to `0`:

```
kit.continuous_servo[1].throttle = 0
```

That's all there is to controlling continuous rotation servos with the the PCA9685 16-channel breakout, Python and `ServoKit`!

Full Example Code

```
# SPDX-FileCopyrightText: 2021 ladyada for Adafruit Industries
# SPDX-License-Identifier: MIT

"""Simple test for a standard servo on channel 0 and a continuous rotation servo on
channel 1."""
import time
from adafruit_servokit import ServoKit

# Set channels to the number of servo channels on your kit.
# 8 for FeatherWing, 16 for Shield/HAT/Bonnet.
kit = ServoKit(channels=8)

kit.servo[0].angle = 180
kit.continuous_servo[1].throttle = 1
time.sleep(1)
kit.continuous_servo[1].throttle = -1
time.sleep(1)
kit.servo[0].angle = 0
kit.continuous_servo[1].throttle = 0
```

Python Docs

Python Docs (https://adafru.it/C5p)

Python Docs: ServoKit

Python Docs: ServoKit (https://adafru.it/Dkx)

Downloads

Files

- PCA9685 datasheet (https://adafru.it/okB)
- Arduino driver library (https://adafru.it/aQI)
- EagleCAD PCB files on GitHub (https://adafru.it/rME)
- Fritzing object in the Adafruit Fritzing library (https://adafru.it/aP3)

Schematic & Fabrication Print

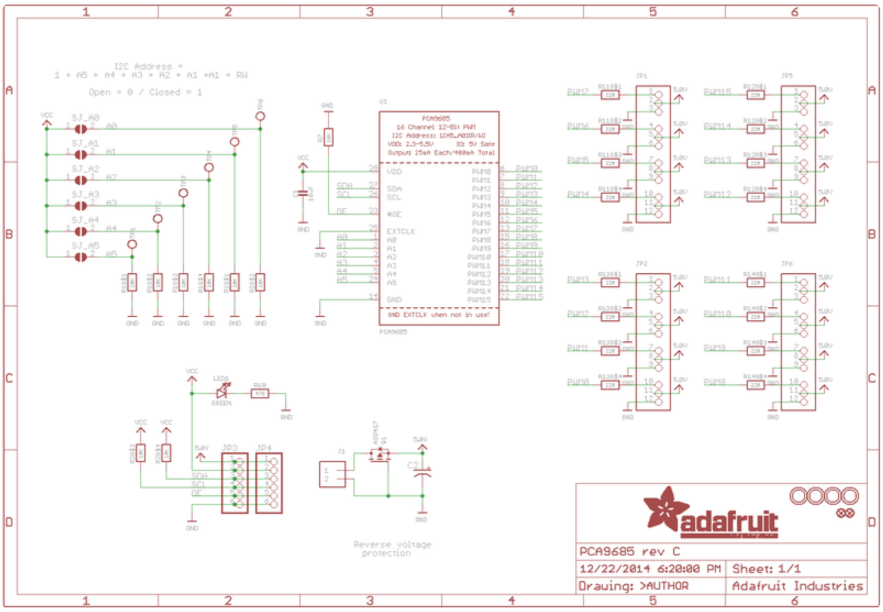

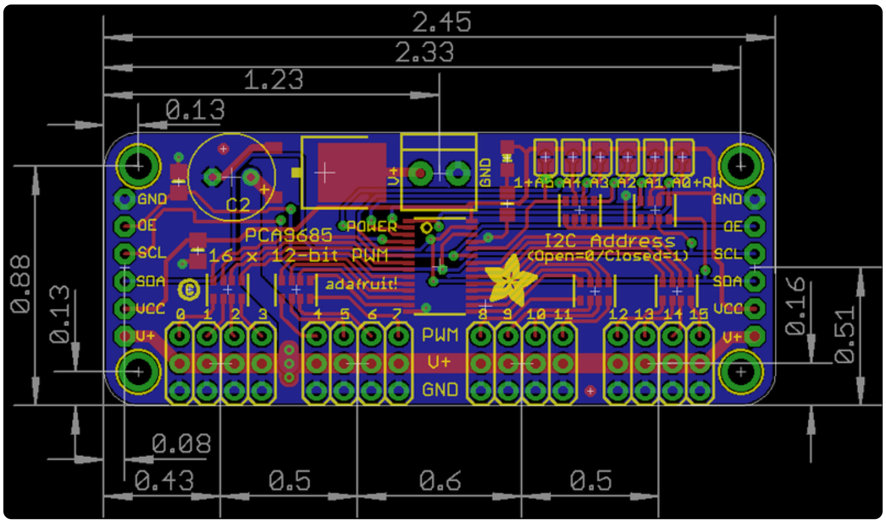

Holes are 2.5mm diameter

FAQ

Can this board be used for LEDs or just servos?

It can be used for LEDs as well as any other PWM-able device!

I am having strange problems when combining this shield with the Adafruit LED Matrix/7Seg Backpacks

The PCA9865 chip has an "All Call" address of 0x70. This is in addition to the configured address. Set the backpacks to address 0x71 or anything other than the default 0x70 to make the issue go away.

With LEDs, how come I cant get the LEDs to turn completely off?

If you want to turn the LEDs totally off use setPWM(pin, 4096, 0); not setPWM(pin, 4095, 0);
