# Supplementary material for: A low-cost, open-source device to evaluate limb stiffness in a rabbit model of cerebral palsy
Source: Front Bioeng Biotechnol. 2025 Jun 5;13:1554775. doi: 10.3389/fbioe.2025.1554775 (PMC12177462; doi:10.3389/fbioe.2025.1554775)
Supplement: Supplementary file 2 [file DataSheet1.zip › MarinManuel-TorqueMeter-772995c/Assets/Datasheets/5862K141_HIGH-PULL RARE EARTH MAGNETIC DISC specs.PDF]

Neodymium Magnet

Magnetized Through Thickness, 1/16" Thick, 1/4" OD

1-49 Each $0.65
50 or more $0.48
5862K141

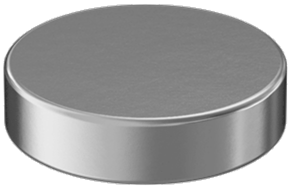

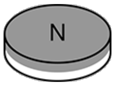

Magnetized Through Thickness

Shape	Disc
Mount Type	Standard
Direction of Magnetization	Through Thickness
Thickness	1/16"
Thickness Tolerance	-0.005" to 0.005"
OD	1/4"
OD Tolerance	-0.005" to 0.005"
Maximum Pull	1.4 lbs.
Sets of Poles	1
Min. Temperature	Not Rated
Maximum Temperature	175° F
Grade	N52
Material	Neodymium (Rare Earth)
Finish	Nickel Plated
Color	Silver
Machinable	No
Maximum Energy Product	414 kJ/m^3
Flux Density	14,800 G
Fabrication	Sintered
System of Measurement	Inch
Additional Specifications	SDS
RoHS	RoHS 3 (2015/863/EU) Compliant
REACH	REACH (EC 1907/2006) (01/19/2021, 211 SVHC) Compliant
DFARS	Not Specialty Metals Compliant
Country of Origin	Peoples Republic of China
Schedule B	850511.0070
ECCN	EAR99
Shipping	Regulated by the U.S. Department of Transportation

Made of neodymium, these rare earth magnets are the strongest for their size. The nickel-plated finish resists corrosion.

Warning: Maximum pull ratings are based on direct contact with rust-free and unpainted iron plate. Variations in material condition will significantly reduce these ratings. Do not use for lifting over people.

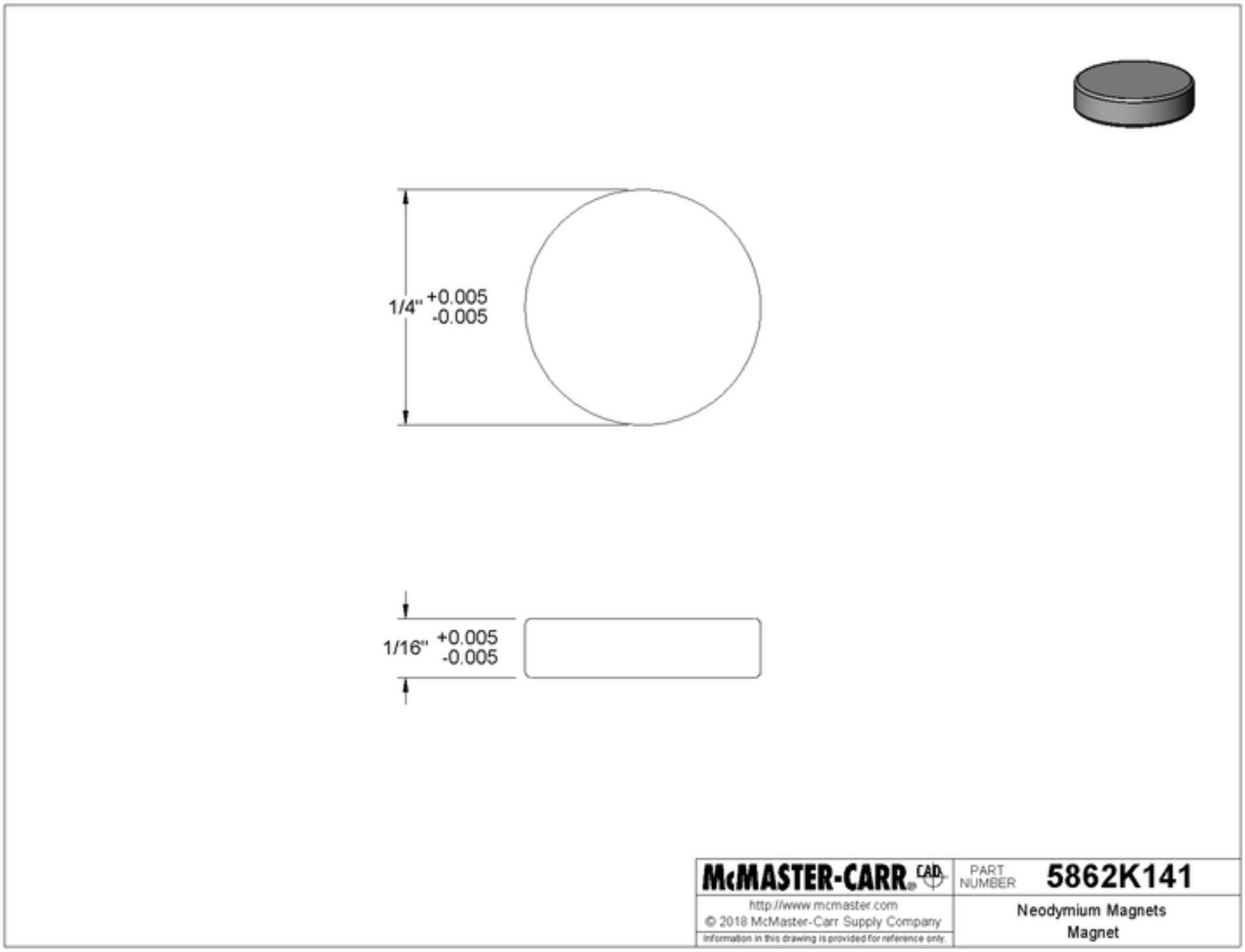

The information in this 3-D model is provided for reference only.
