# Supplementary material for: A low-cost, open-source device to evaluate limb stiffness in a rabbit model of cerebral palsy
Source: Front Bioeng Biotechnol. 2025 Jun 5;13:1554775. doi: 10.3389/fbioe.2025.1554775 (PMC12177462; doi:10.3389/fbioe.2025.1554775)
Supplement: Supplementary file 2 [file DataSheet1.zip › MarinManuel-TorqueMeter-772995c/Assets/Datasheets/6455K270_GENERAL PURPOSE PLASTIC BALL BEARING specs.PDF]

McMASTER-CARR®

Plastic Ball Bearing

with 316 Stainless Steel Ball, Trade No. R10A, for 1/2" Shaft Diameter

$6.95 Each
6455K27

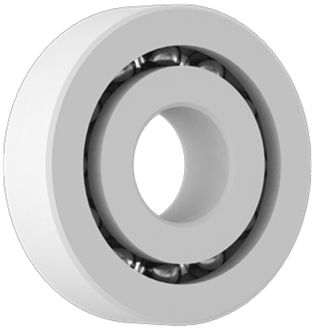

Bearing Trade Number	R10A
Bearing Type	Ball
For Load Direction	Radial
Construction	Single Row
Seal Type	Open
Inner Ring Type	Standard
Ball Bearing Type	Standard
For Shaft Type	Round
For Shaft Diameter	1/2"
ID	0.5"
ID Tolerance	0" to 0.003"
For Housing ID	1 3/8"
OD	1.375"
OD Tolerance	-0.004" to 0"
Width	3/8"
Width Tolerance	-0.005" to 0.005"
Ring Material	Acetal Plastic
Cage Material	Acetal Plastic
Radial Load Capacity, lbs.	
Dynamic	65
Static	45
Maximum Speed	1,000 rpm
Lubrication	Not Required
Shaft Mount Type	Press Fit
Temperature Range	-40° to 180° F
ABEC Rating	Not Rated
Radial Clearance	0.001" to 0.009"
Ball Material	316 Stainless Steel
RoHS	RoHS 3 (2015/863/EU) Compliant
REACH	REACH (EC 1907/2006) (01/19/2021, 211 SVHC) Compliant
DFARS	Specialty Metals COTS-Exempt
Country of Origin	United States
USMCA Qualifying	No
Schedule B	848210.5048
ECCN	1C999

These acetal bearings have good all-around corrosion and chemical resistance.

316 stainless steel balls have excellent corrosion resistance.
