# Supplementary material for: A low-cost, open-source device to evaluate limb stiffness in a rabbit model of cerebral palsy
Source: Front Bioeng Biotechnol. 2025 Jun 5;13:1554775. doi: 10.3389/fbioe.2025.1554775 (PMC12177462; doi:10.3389/fbioe.2025.1554775)
Supplement: Supplementary file 2 [file DataSheet1.zip › MarinManuel-TorqueMeter-772995c/Assets/Datasheets/91292A312 18-8 Stainless Steel Socket Head Screw, M2.5x22mm.pdf]

McMASTER-CARR®

18-8 Stainless Steel Socket Head Screw

M2.5 x 0.45 mm Thread, 22 mm Long

$10.26 per pack of 25
91292A312

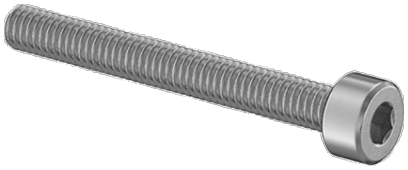

Head Type	Socket
Socket Head Profile	Standard
Drive Style	Hex
System of Measurement	Metric
Thread Direction	Right Hand
Thread Size	M2.5
Thread Pitch	0.45 mm
Thread Type	Metric
Thread Fit	Class 6g
Length	22 mm
Threading	Fully Threaded
Thread Spacing	Coarse
Head	
Diameter	4.5 mm
Height	2.5 mm
Drive Size	2 mm
Material	18-8 Stainless Steel
Tensile Strength	70,000 psi
Hardness	Not Rated
Specifications Met	DIN 912, ISO 4762
RoHS	RoHS 3 (2015/863/EU) Compliant
REACH	REACH (EC 1907/2006) (06/10/2022, 224 SVHC) Compliant
DFARS	Not Specialty Metals Compliant
Country of Origin	Belgium, France, Germany, India, Indonesia, Italy, Japan, Malaysia, Peoples Republic Of China, Philippines, Poland, South Korea, Switzerland, Taiwan, Thailand, or Vietnam
Schedule B	731815.9000
ECCN	EAR99

Made from 18-8 stainless steel, these screws have good chemical resistance and may be mildly magnetic. Length is measured from under the head.

Metric screws are also known as A2 stainless steel screws.

Coarse threads are the industry standard; choose these screws if you don’t know the pitch or threads per inch.

Screws that meet ASME B1.1, ASME B18.3, ISO 21269, and ISO 4762 (formerly DIN 912) comply with standards for dimensions.

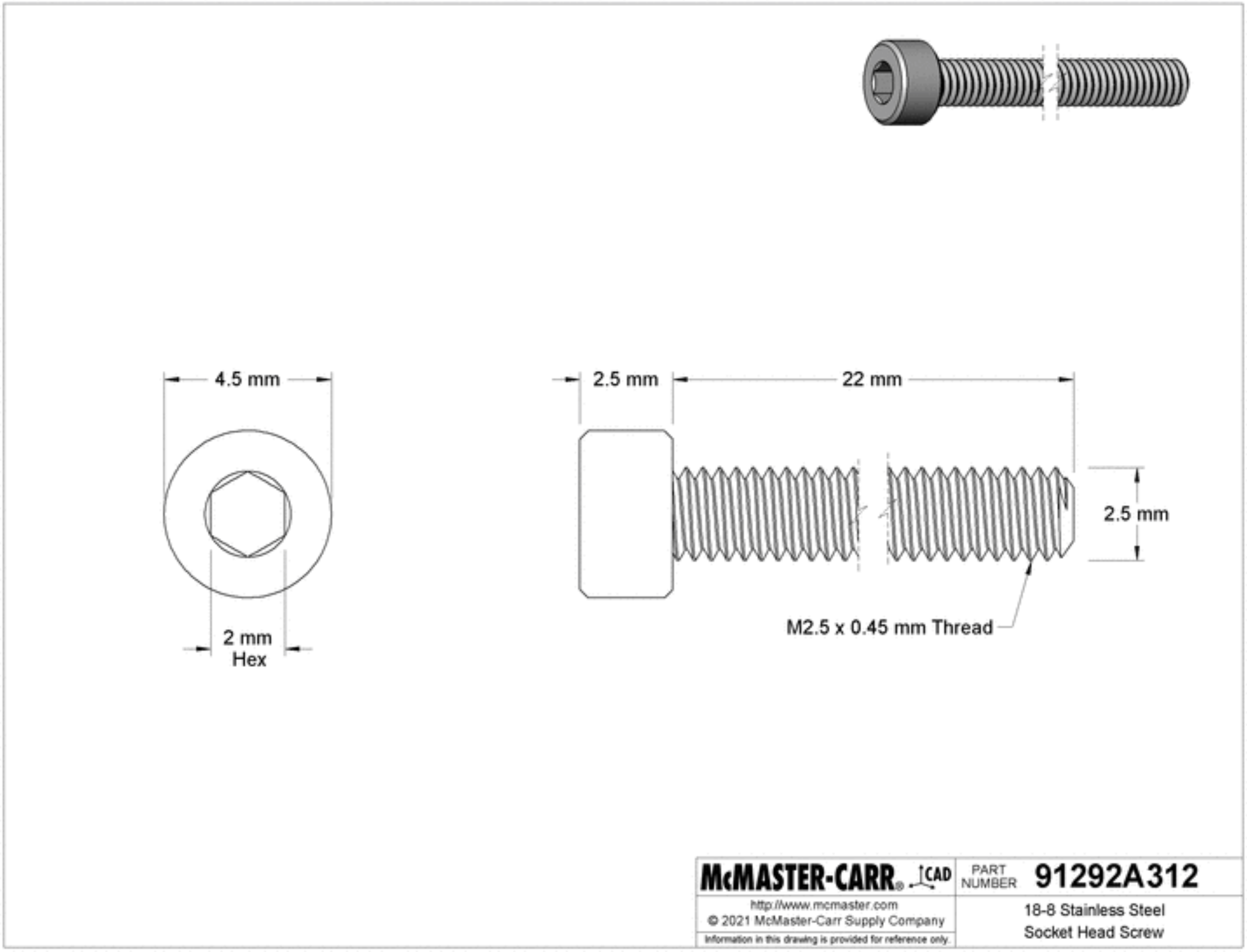

The information in this 3-D model is provided for reference only.
