# Supplementary material for: A low-cost, open-source device to evaluate limb stiffness in a rabbit model of cerebral palsy
Source: Front Bioeng Biotechnol. 2025 Jun 5;13:1554775. doi: 10.3389/fbioe.2025.1554775 (PMC12177462; doi:10.3389/fbioe.2025.1554775)
Supplement: Supplementary file 2 [file DataSheet1.zip › MarinManuel-TorqueMeter-772995c/Assets/Datasheets/91828A211 18-8 Stainless Steel Hex Nut, M3.pdf]

McMASTER-CARR®

18-8 Stainless Steel Hex Nut

$4.73 per pack of 100
91828A211

M3 x 0.5 mm Thread

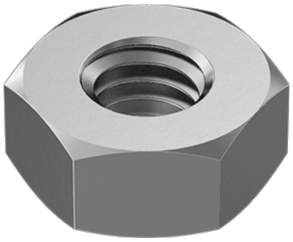

Material	18-8 Stainless Steel
Thread	
Size	M3
Pitch	0.5 mm
Type	Metric
Spacing	Coarse
Fit	Class 6H
Direction	Right Hand
Width	5.5 mm
Height	2.4 mm
Specifications Met	DIN 934
Drive Style	External Hex
Nut Type	Hex
Hex Nut Profile	Standard
System of Measurement	Metric
RoHS	RoHS 3 (2015/863/EU) Compliant
REACH	REACH (EC 1907/2006) (06/10/2022, 224 SVHC) Compliant
DFARS	Not Specialty Metals Compliant
Country of Origin	Varies
Schedule B	731816.0000
ECCN	EAR99

These nuts have good chemical resistance and may be mildly magnetic. Metric 18-8 stainless steel is also known as A2 stainless steel.

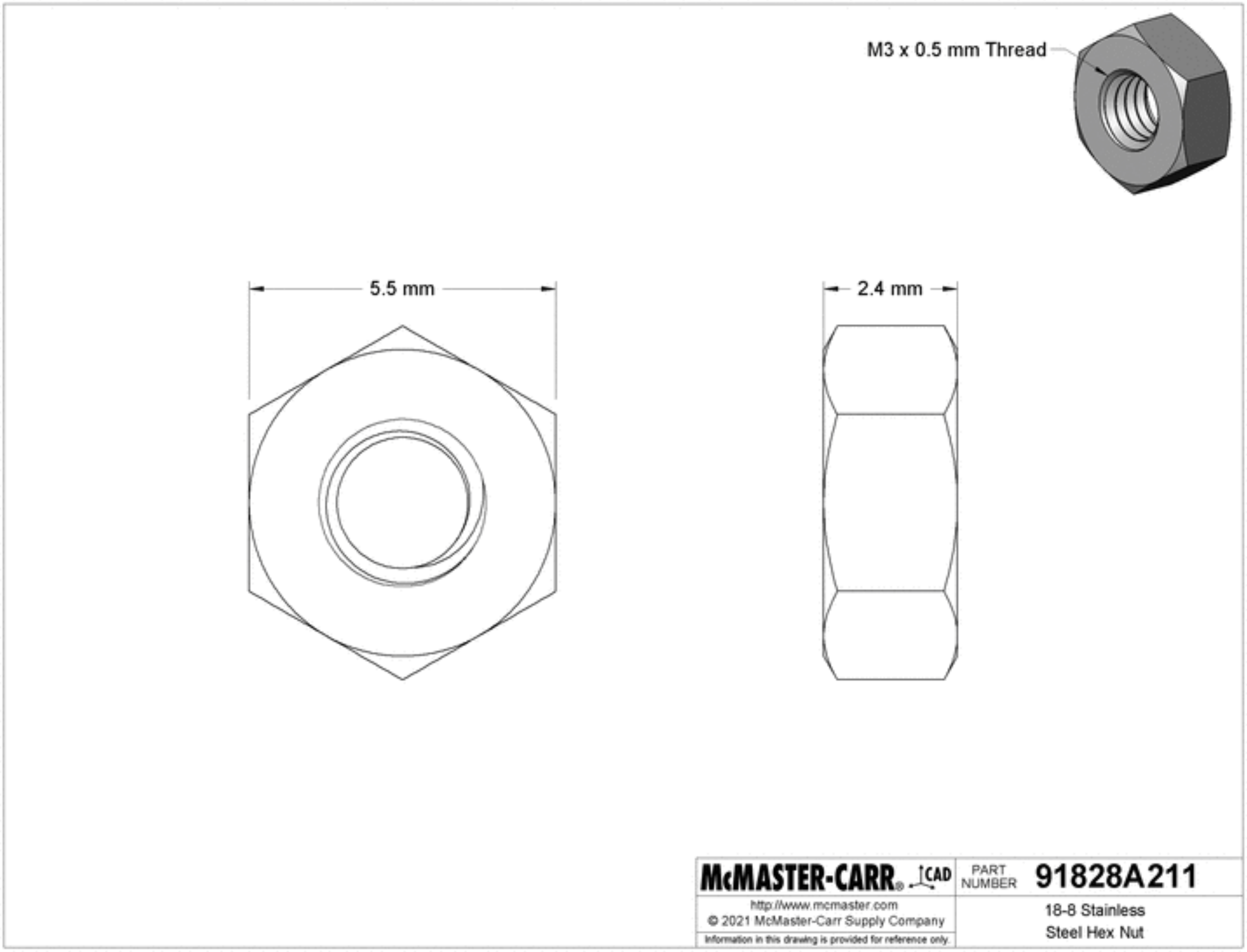

The information in this 3-D model is provided for reference only.
