# Supplementary material for: A low-cost, open-source device to evaluate limb stiffness in a rabbit model of cerebral palsy
Source: Front Bioeng Biotechnol. 2025 Jun 5;13:1554775. doi: 10.3389/fbioe.2025.1554775 (PMC12177462; doi:10.3389/fbioe.2025.1554775)
Supplement: Supplementary file 2 [file DataSheet1.zip › MarinManuel-TorqueMeter-772995c/Assets/Datasheets/92095A187 18-8 Stainless Steel Socket Head Screw M3x30mm.pdf]

Button Head Hex Drive Screw

Passivated 18-8 Stainless Steel, M3 x 0.50 mm Thread, 30mm Long

$7.85 per pack of 50
92095A187

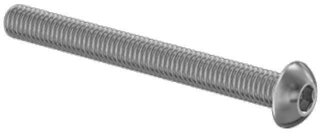

Thread Size	M3
Thread Pitch	0.5 mm
Length	30 mm
Threading	Fully Threaded
Head Diameter	5.70 mm
Head Height	1.65 mm
Drive Style	Hex
Drive Size	2 mm
Material	Passivated 18-8 Stainless Steel
Hardness	Not Rated
Tensile Strength	70,000 psi
Thread Type	Metric
Thread Spacing	Coarse
Thread Fit	Class 6g
Thread Direction	Right Hand
Head Type	Rounded
Rounded Head Style	Button
Rounded Head Profile	Standard
System of Measurement	Metric
Specifications Met	ISO 7380
RoHS	RoHS 3 (2015/863/EU) Compliant
REACH	REACH (EC 1907/2006) (06/10/2022, 224 SVHC) Compliant
DFARS	Not Specialty Metals Compliant
Country of Origin	Varies
Schedule B	731815.9000
ECCN	EAR99

Made from 18-8 stainless steel, these button head screws have good chemical resistance and may be mildly magnetic. Length is measured from under the head.

Passivated stainless steel screws provide added protection against oxidation and corrosion.

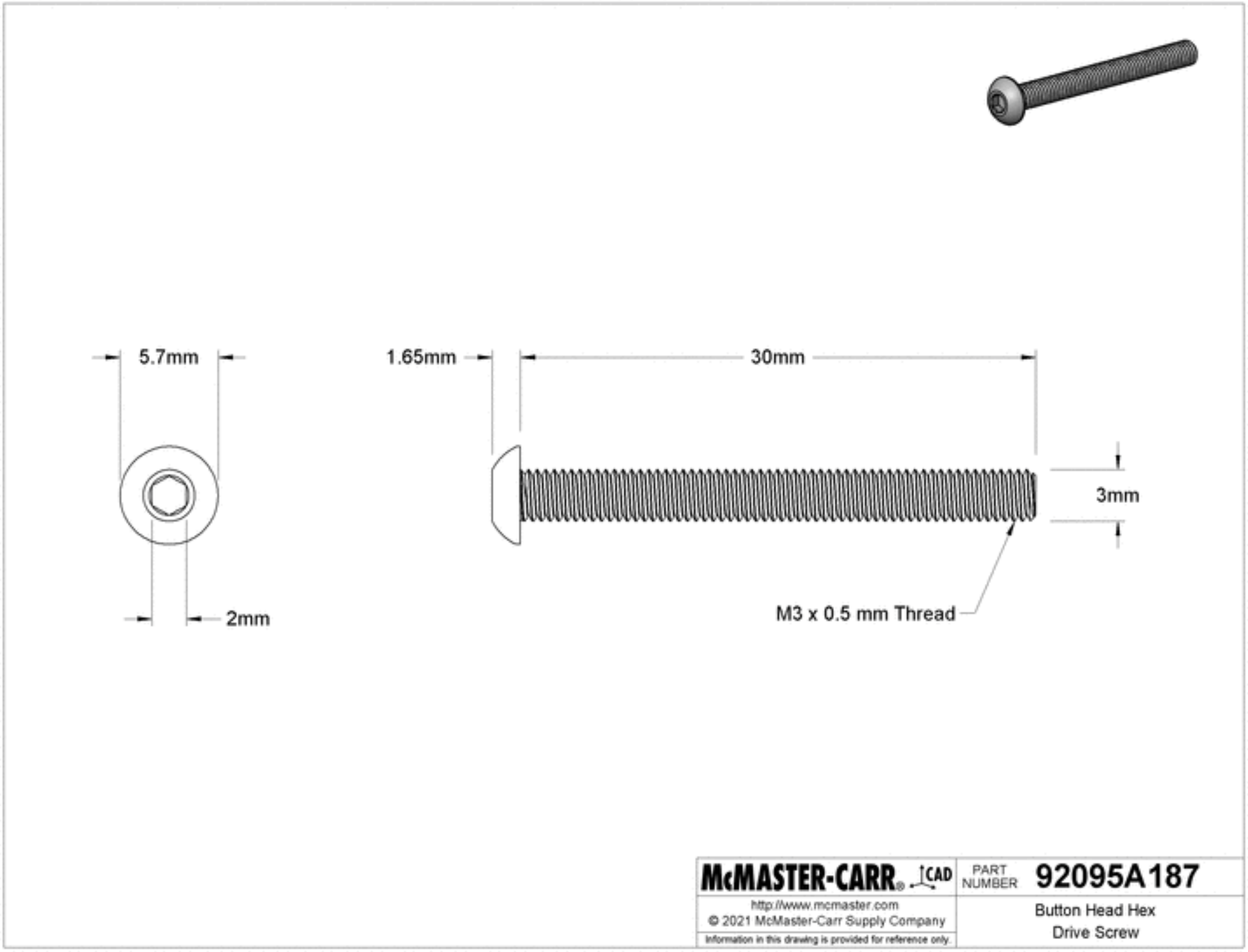

The information in this 3-D model is provided for reference only.
