# Supplementary material for: A low-cost, open-source device to evaluate limb stiffness in a rabbit model of cerebral palsy
Source: Front Bioeng Biotechnol. 2025 Jun 5;13:1554775. doi: 10.3389/fbioe.2025.1554775 (PMC12177462; doi:10.3389/fbioe.2025.1554775)
Supplement: Supplementary file 2 [file DataSheet1.zip › MarinManuel-TorqueMeter-772995c/Assets/Datasheets/9309K79_TIGHT-GRIP PUSH-IN BUMPERS specs.PDF]

McMASTER-CARR®

Tight-Grip Push-In Bumpers

for 5/16" ID and 9/32" Thickness, 13/32" Ridge Diameter, 1/4" High, SBR

$9.31 per pack of 10
9309K79

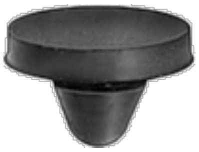

2

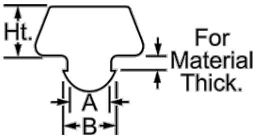

1-3

Mount Type	Push-In Stem
Shape	Round
Material	SBR Rubber
For ID (A)	5/16"
Ridge Diameter (B)	13/32"
For Material Thickness	9/32"
OD	7/8"
Height	1/4"
Temperature Range	-20° to 180° F
For Use Outdoors	No
Hardness Rating	Medium Hard
Hardness	Durometer 60A
Nonmarking	No
Color	Black
RoHS	RoHS 3 (2015/863/EU) Compliant
REACH	REACH (EC 1907/2006) (01/19/2021, 211 SVHC) Compliant
DFARS	Specialty Metals COTS-Exempt
Country of Origin	United States
USMCA Qualifying	Yes
Schedule B	401699.6000
ECCN	EAR99

A ridge on the stem provides a tighter grip than other push-in bumpers. Push the stem into a hole for quick installation. These bumpers are commonly used as feet on instruments and bench-top equipment to keep them from sliding. They're also good as spacers between sheets of material. Also known as stem and grommet bumpers.

Bumpers with a hard and medium-hard rating withstand high levels of impact. The harder the bumper, the more impact it withstands.

SBR bumpers have good abrasion resistance. They are not for use outdoors.

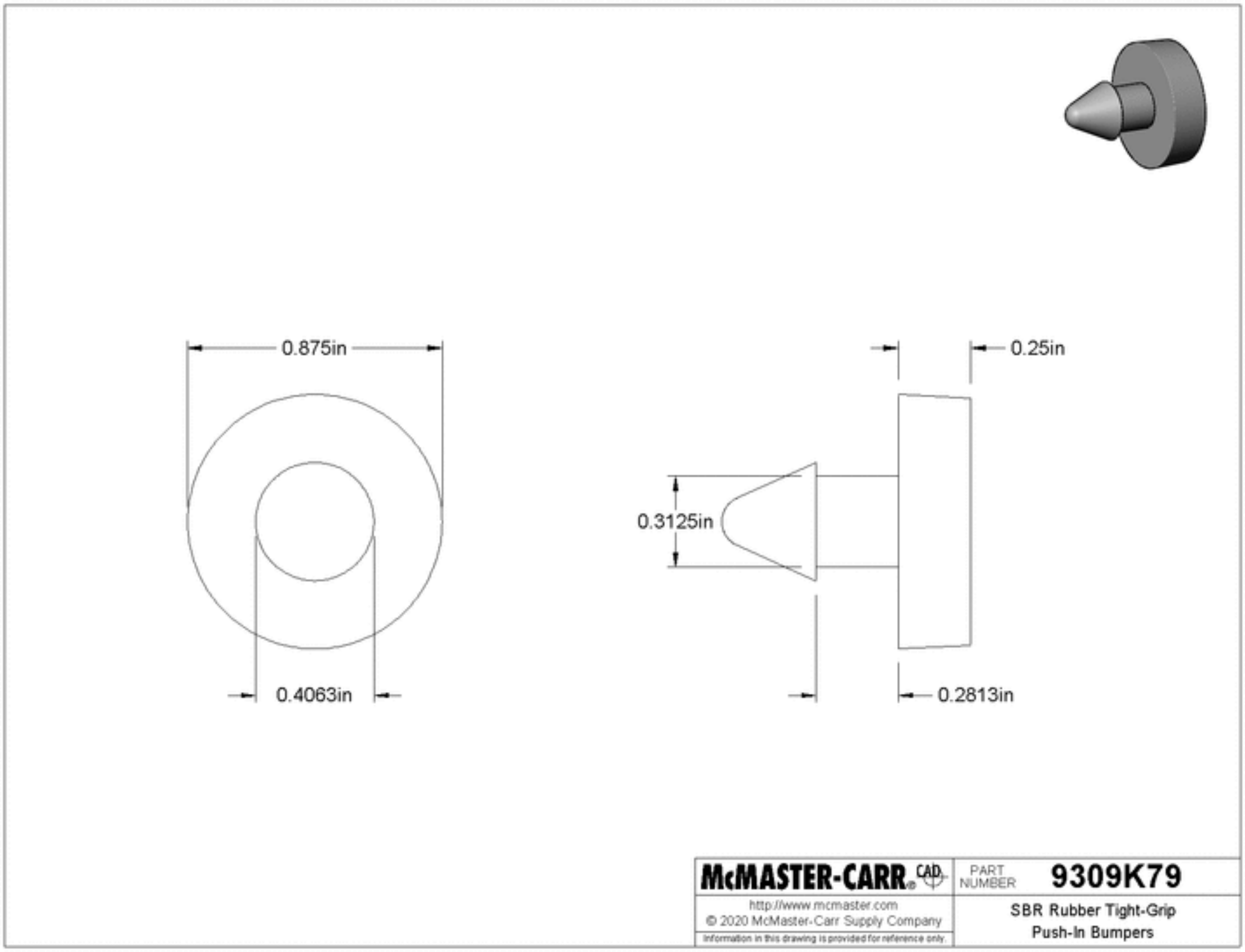

The information in this 3-D model is provided for reference only.
