# Supplementary material for: A low-cost, open-source device to evaluate limb stiffness in a rabbit model of cerebral palsy
Source: Front Bioeng Biotechnol. 2025 Jun 5;13:1554775. doi: 10.3389/fbioe.2025.1554775 (PMC12177462; doi:10.3389/fbioe.2025.1554775)
Supplement: Supplementary file 2 [file DataSheet1.zip › MarinManuel-TorqueMeter-772995c/Assets/Datasheets/Load Cell Amplifier HX711 Breakout Hookup Guide.pdf]

Load Cell Amplifier HX711 Breakout Hookup Guide

CONTRIBUTORS: *SARAH AL-MUTLAQ*, *ALEX THE GIANT*

FAVORITE 7

Introduction

The HX711 load cell amplifier is used to get measurable data out from a load cell and strain gauge. This Hookup Guide will show you how to get started with this amplifier using some of the various load cells we carry at SparkFun.

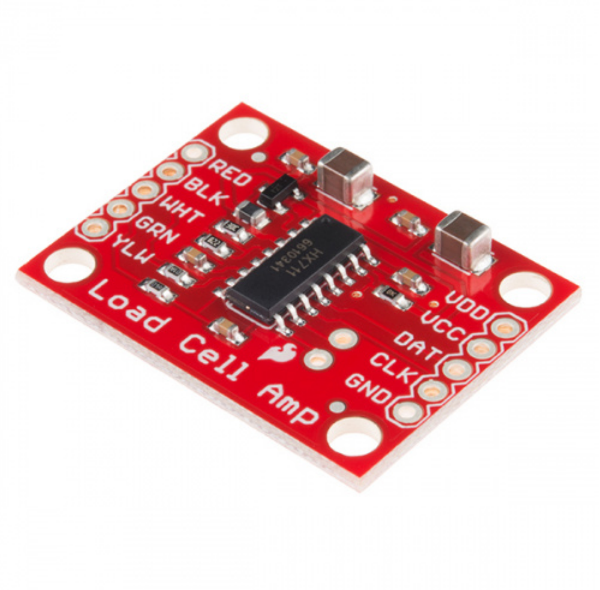

SparkFun Load Cell Amplifier - HX711
SEN-13879
$9.95
38

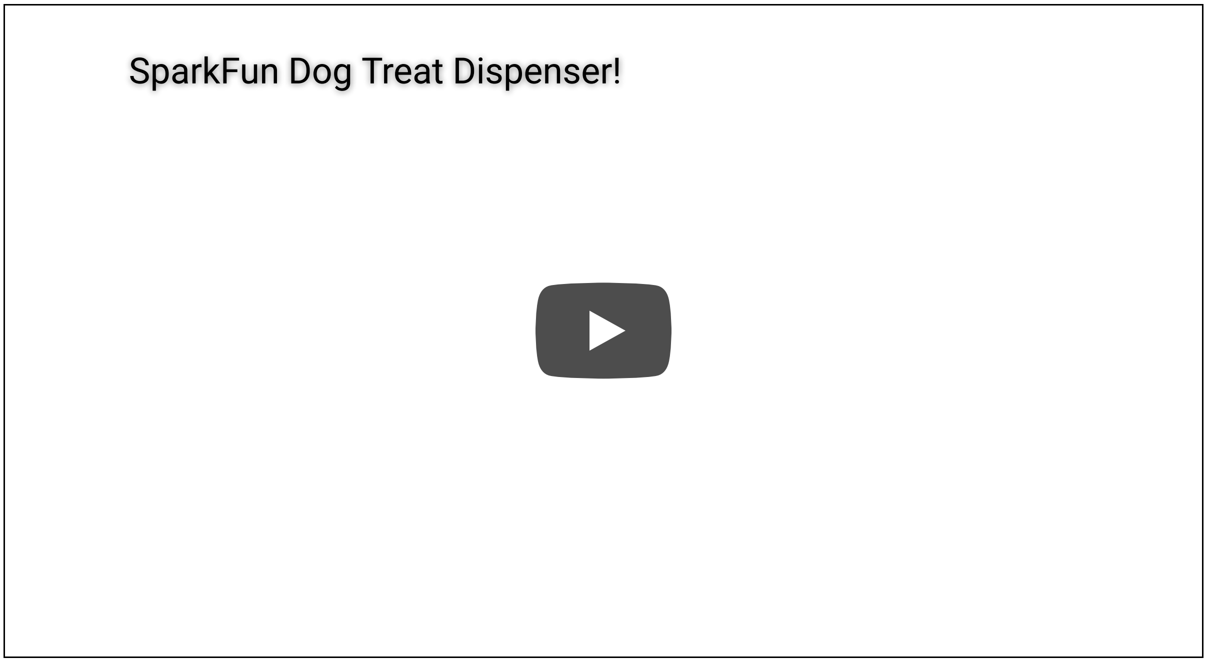

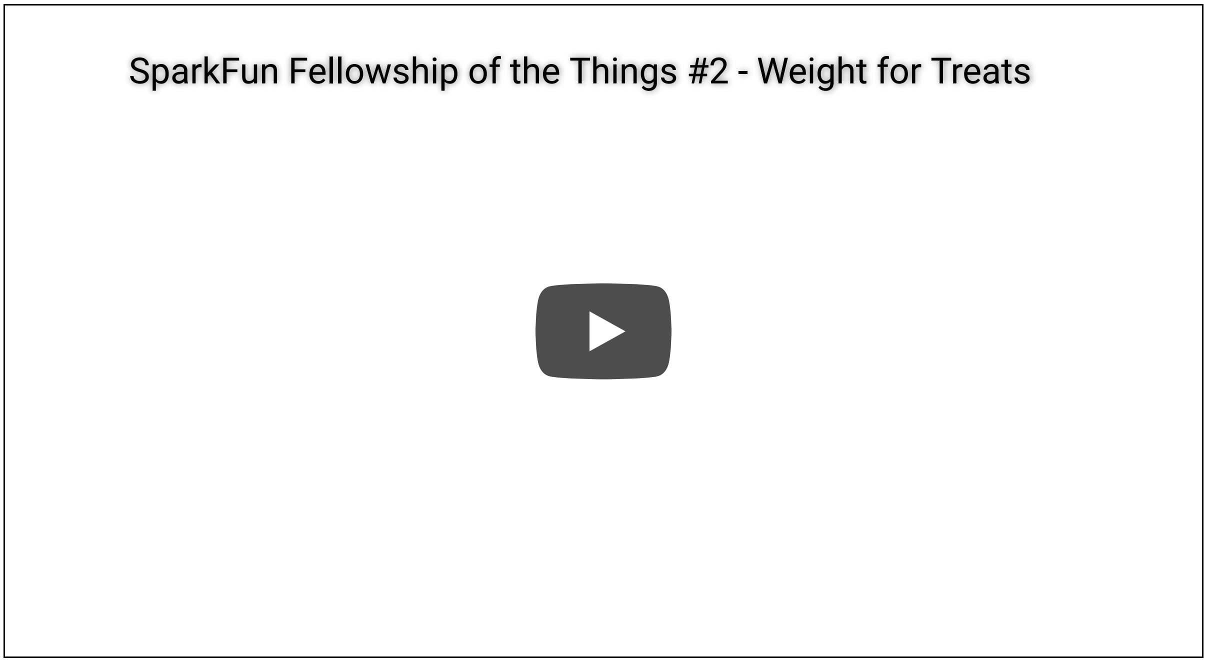

Required Materials

For this simple hookup guide, we will just be hooking up a load cell with the HX711 amplifier, and showing how you would hook up four load sensors with a combinator board and the HX711 amplifier. To follow along, you'll need:

- SparkFun Load Cell Amplifier - HX711
- Any Strain Gauge Based Load Cell:

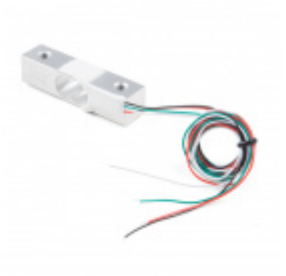

Load Cell - 5kg, Straight Bar (TAL220B)

SEN-14729

$10.95

★★★★★ 1

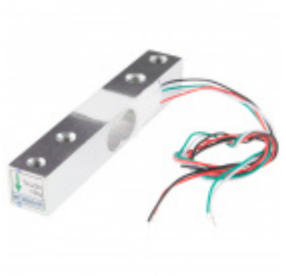

Load Cell - 10kg, Straight Bar (TAL220)

SEN-13329

$8.50

★★★★☆ 3

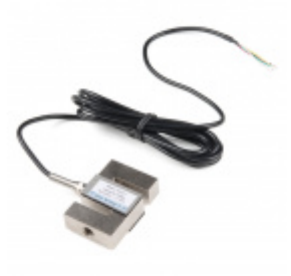

Load Cell - 200kg, S-Type (TAS501)

SEN-14282

$59.95

★★★★½ 3

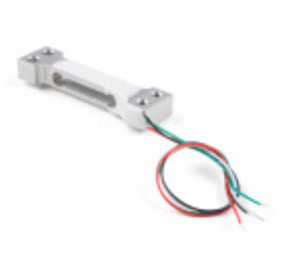

Mini Load Cell - 500g, Straight Bar (TAL221)

SEN-14728

$9.95

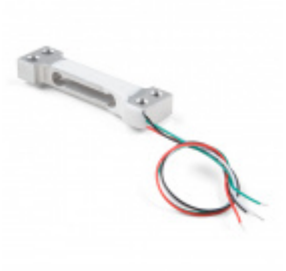

Mini Load Cell - 100g, Straight Bar (TAL221)

SEN-14727

$8.95

★★★★☆ 1

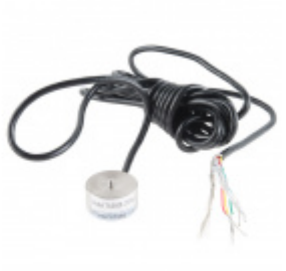

Load Cell - 200kg, Disc (TAS606)

SEN-13332

$59.95

★★★★½ 4

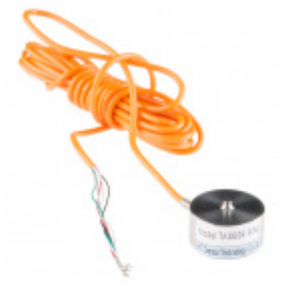

Load Cell - 50kg, Disc (TAS606)
SEN-13331
$59.95
☆☆☆☆☆ 1

If you are planning on using load sensors[1] you will need to obtain or purchase four units. We recommend our Combinator Board to make it easy to turn the four strain gauges into a wheatstone bridge type load cell. (Single strain gauge load cells only have three wires instead of four.)

Suggested Reading

If you aren't familiar with the following concepts, we recommend reviewing them before beginning to work with the HX711 Load Cell Amplifier Board.

How to Solder: Through-Hole Soldering
This tutorial covers everything you need to know about through-hole soldering.

Battery Technologies
The basics behind the batteries used in portable electronic devices: LiPo, NiMH, coin cells, and alkaline.

How to Power a Project
A tutorial to help figure out the power requirements of your project.

Getting Started with Load Cells
A tutorial defining what a load cell is and how to use one.

1. [Strain gauges are two wired organized metal foil or wires that are set up in such a way that the resistance changes when it is compressed or stretched. When a strain gauge is placed on something (usually metallic in nature) its resistance changes based on the stress experienced by that something. When a single strain gauge is hooked up to a metallic cell, we are calling that a load sensors, which have three output wires. Load cells usually has four strain gauges hooked up in a wheatstone bridge formation, which have four output wires. For more information on load cells, strain gauges, and wheatstone bridges see our tutorial.]↩

Load Cell Setup

Depending on the type of load cell you are using, the configuration of how it should be hooked up to plates or surfaces will change. We'll list a few different types of setups below.

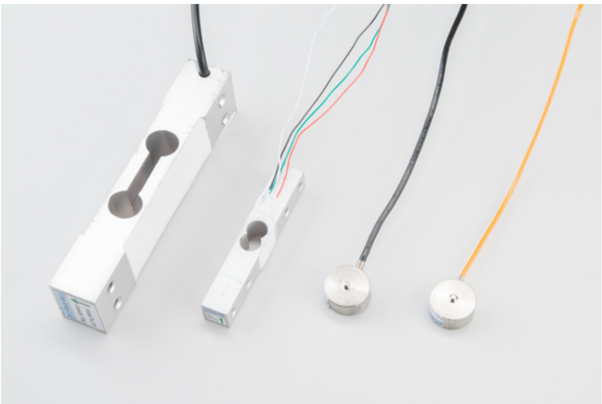

A selection of different load cells

Bar-Type Load Cell

Usually with larger, non-push button bar load cells you will want to hook up the load cell between two plates in a "Z" shape.

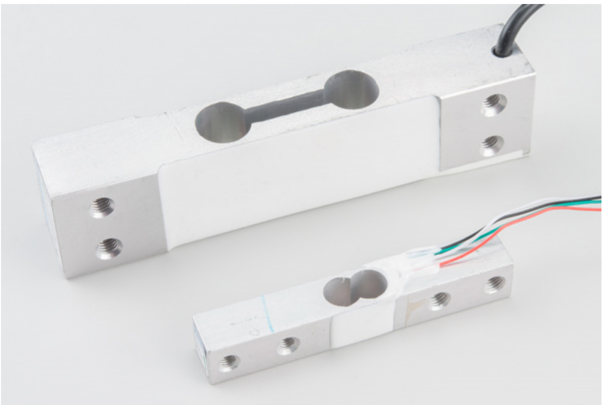

Bar strain gauge based load cells

As shown below, the bar-type load cell is mounted with fitting screws and spacers so that the strain can be correctly measured. Note that only one side of the load cell is screwed into each board when a bar-type load cell is placed between two plates. This provides a moment of force, or torque, on the strain gauge rather than just compression force, which is easier to measure and much more accurate.

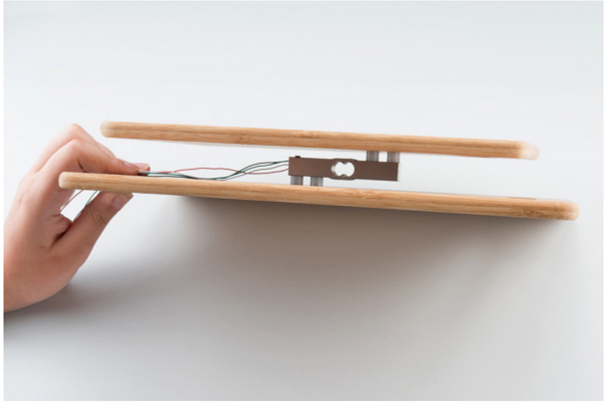

Bar load cell between a two plate configuration

S-Type Load Cell

S-type load cells can measure compression, tension, or both due to its design.

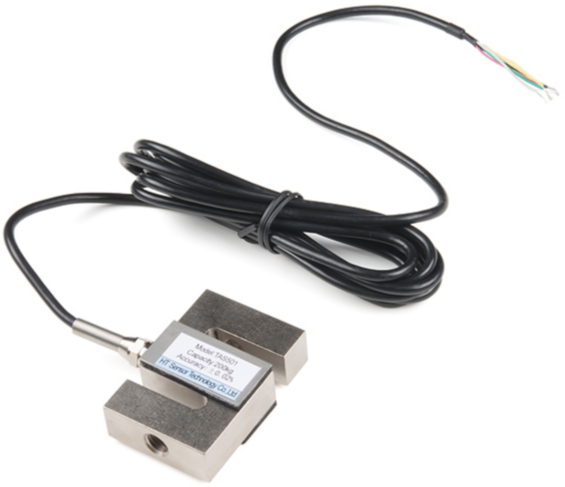

Besides placing the load cell between plates, the s-type can be used to measure suspended tanks or hoppers. You can think of this as a load attached to a crane. Below is an s-type load cell with two threaded, rod end bearings.

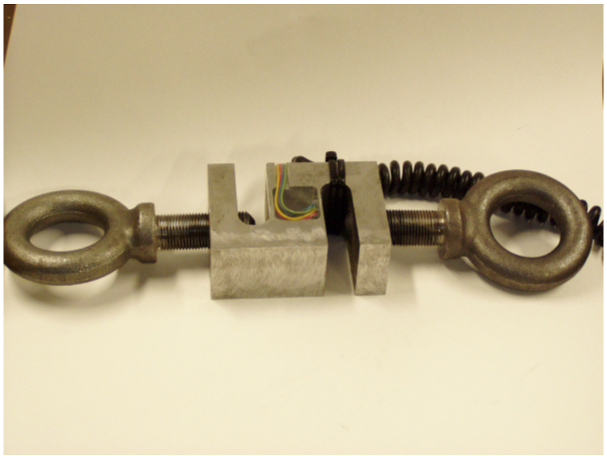

S-type load cell configuration

Single, Strain Gauge Load Cells (i.e. Load Sensors)

For single, strain gauge load cells, they can be placed at equal distances with respect to each other underneath a platform.

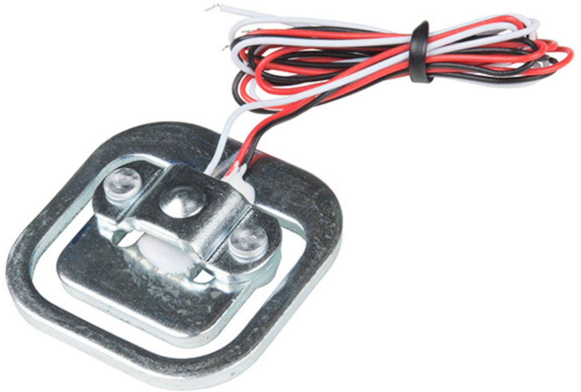

The image below shows four, single strain gauge (i.e. the load sensor) arranged in a wheatstone bridge configuration. This configuration is also possible with four button-type load cells.

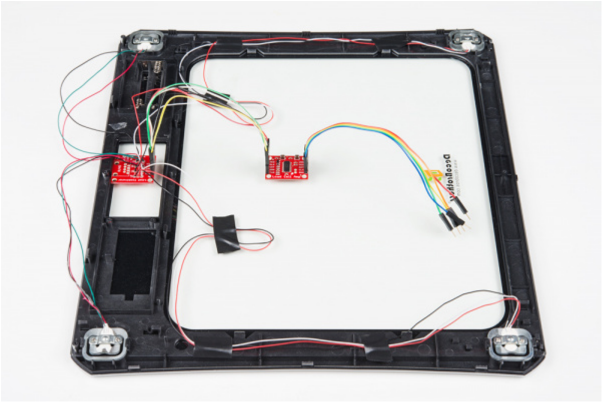

SparkFun's strain gauge load cell combinator board hooked up to a home scale, possible four disc load cell configuration in something like a bathroom scale

Note: If you are hooking together four of the SparkFun Load Sensors using the Combinator board, you should position the four load sensors **equidistant** from each other, just like the bathroom scales shown in this tutorial.

Button/Disk-Type Load Cells

For smaller, push-button or disc load cells, you will want to make sure to screw in the disc to a bottom plate (or surface you are measuring force against), and center the beam, plate, or whatever else you are wishing to measure the force of onto the "button" on the top.

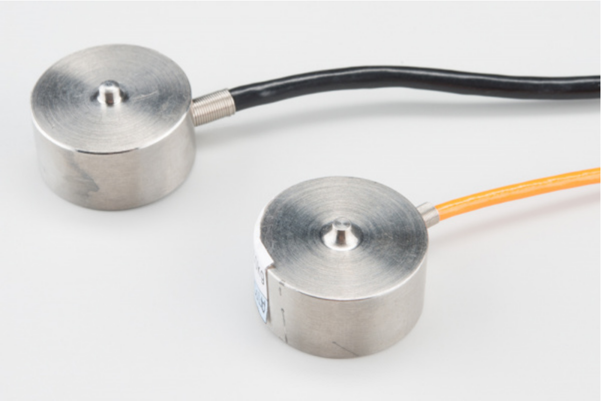

Usually another plate with a hole is used to make sure whatever you are measuring is hitting the same spot on the load cell each time, but it is not necessary. Below are images of four button-type load cells placed at equal distances with respect to each other under a platform. Make sure to read the datasheet for the load cell you are using and get the correct screws to fit into it.

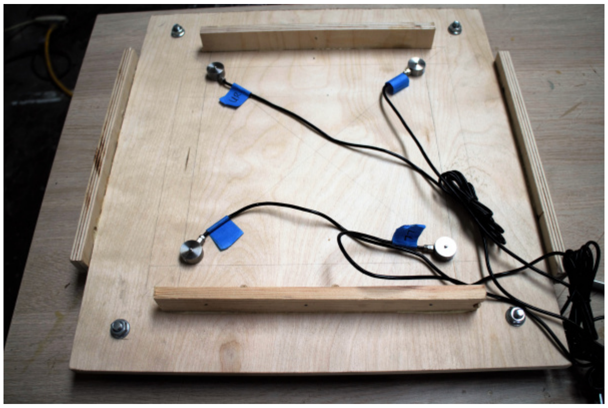	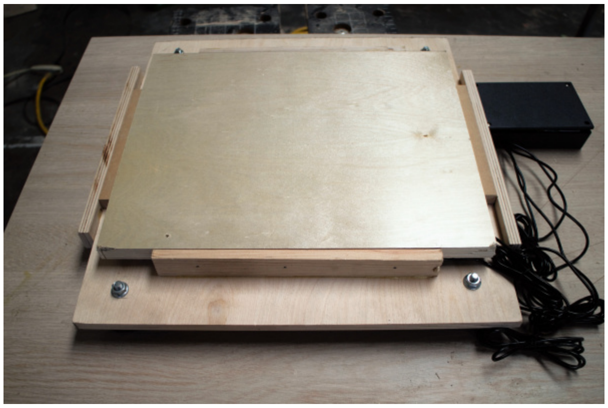
Push Button Load Cells from the IoT Industrial Scale tutorial	*Push Button Load Cells Inside a Custom Built Scale*

Variances in Measurements

Load cell measurements can be off by +/- 5% due to a range of things including temperature, creep, vibration, drift, and other electrical and mechanical interferences. Before you install your scale, take a moment and design your system to allow for easy calibration or be able to adjust the code parameters to account for these variations.

Hardware Hookup

The HX711 Load Cell Amplifier accepts five wires from the load cell. These pins are labeled with colors; **RED, BLK, WHT, GRN, and YLW**.

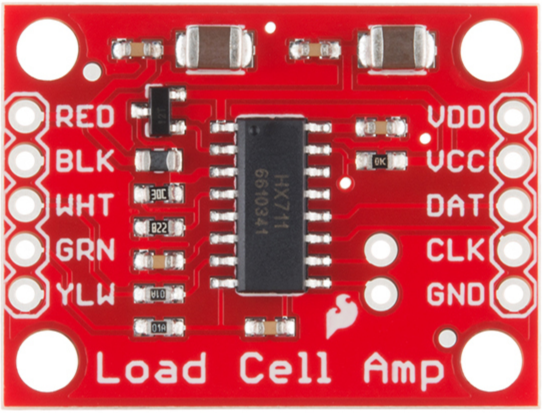

These colors correspond to the conventional color coding of load cells, where red, black, green and white wires come from the strain gauge on the load cell and yellow is an optional ground wire that is not hooked up to the strain gauge but is there to ground any small outside EMI (electromagnetic interference). Sometimes instead of a yellow wire there is a larger black wire, foil, or loose wires to shield the signal wires to lessen EMI.

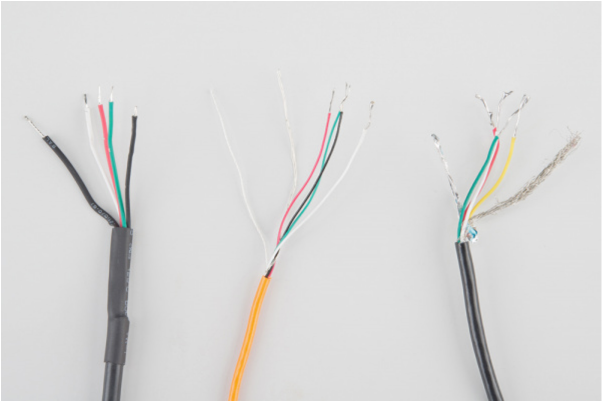

Here we have a large black wire, some loose wires, and foil and loose wires respectively as EMI buffers

In general, each load cell has four strain gauges that are hooked up in a **wheatstone bridge** formation as shown below.

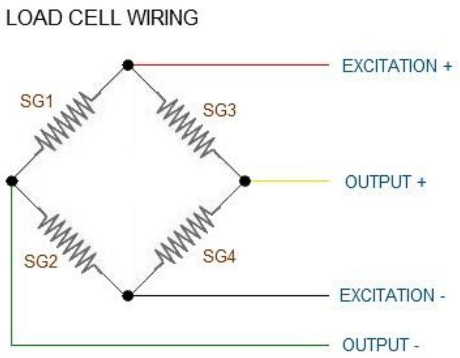

Four strain gauges (SG1 through 4) hooked up in a wheatstone bridge formation

The four wires coming out from the wheatstone bridge on the load cell are "usually":

Wheatstone Bridge Node	"Typical" Wire Color
Excitation+ (E+) or VCC	RED

Excitation- (E-) or GND	BLACK or YELLOW
Output- (O-), Signal- (S-), or Amplifier- (A-)	WHITE
O+, S+, or A+	GREEN or BLUE

Some load cells might have slight variations in color coding such as blue instead of green or yellow instead of black or white if there are only four wires (meaning no wire used as an EMI buffer). You might have to infer a little from the colors that you have or check the datasheet on the load cell, but in general you will usually see these colors.

If the readings from the HX711 are opposite of what you are expect (for example the values decrease as you increase weight) simply reverse the O+/O- wires.

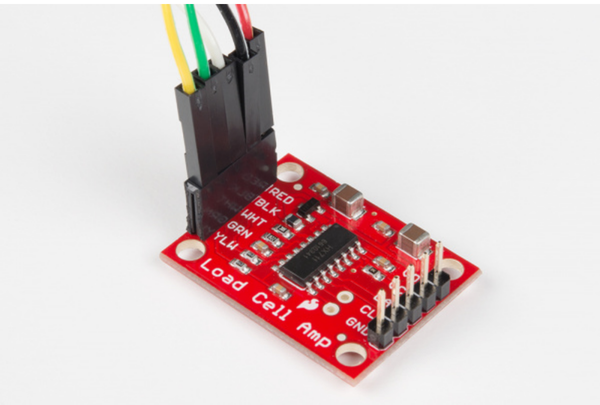

Load cell wires hooked up to the HX711 Amplifier board

Once the load cell is is hooked up to the amplifier, you can hook up VDD, VCC, DAT, CLK, and GND to a microcontroller such as a RedBoard or Arduino board.

Note: VCC is the analog voltage to power the load cell. VDD is the digital supply voltage used to set the logic level.

PRO TIP: In many cases, you can just short VCC and VDD together. If your microcontroller uses 3.3V logic however, you'll want to connect VCC to 5V and VDD to 3.3V.

The example code has **DAT and CLK** hooked up to **pin 3 and 2** respectively, but this is easily changed in the code. Any GPIO pin will work for either. Then VCC and VDD just need to be hooked up to **2.7-5V** and GND to ground on your microcontroller.

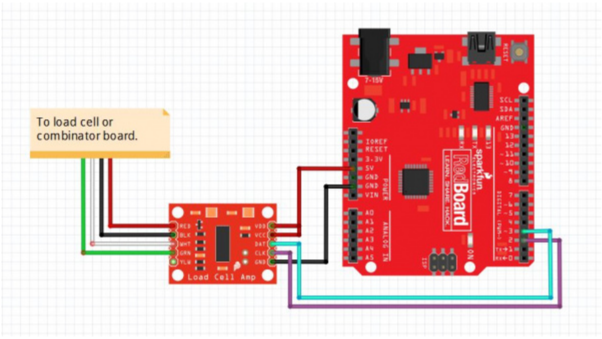

Fritzing diagram of HX711 amplifier connected to a RedBoard

Strain Gauges with the Load Cell Combinator Board

Now, if you would like to set up four single load sensors with our combinator board and amplifier, connect the five pins labeled RED, BLK, WHT, GRN, YLW on the combinator to the matching pins on the amplifier. Next, connect each of the four load sensors to the following pins:

Single, Strain Gauge (i.e. Load Sensor) "Typical" Wire Color	Load Combinator Board
RED	C
WHITE	+
BLACK	-

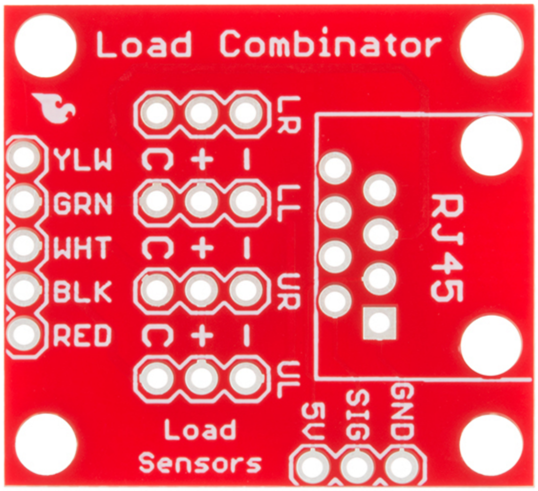

Note: The combinator board also has room for an 8 pin RJ45 socket, which can be used to connect your project via Ethernet cables for long distance applications.

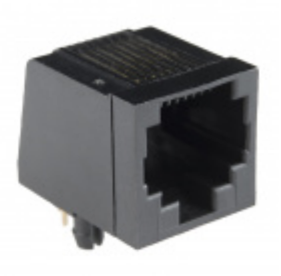

RJ45 8-Pin Connector
PRT-00643
$1.50
★★★★★ 1

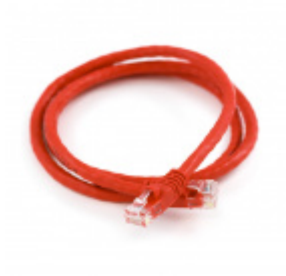

CAT 6 Cable - 3ft
CAB-08915
$1.95

Another nice thing about our combinator board is that most home scales use four single strain gauge load sensors, so this is a handy board for hacking your own scales at home!

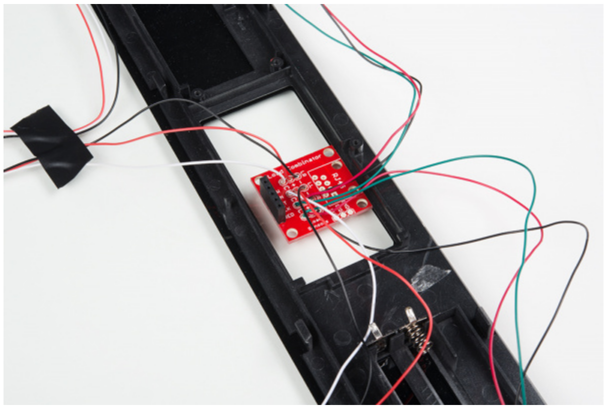

Hacked home scale's four load sensors hooked up to our combinator board

For load sensors, there isn't a set color coded standard. The hacked home scale can have different wire colors. Comparing the scale pictured above with the load sensor schematic, while the black wires matched, the red and white wires were swapped. Also, only two of the four sensors used a white wire for the 'center tap' of the load sensor, the other two used green. I connected the black wires to "**-**", the red to "**+**", and the white and green wires to "**C**".

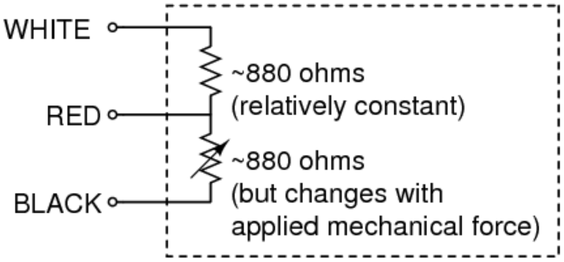

Example of a single strain gauge, or load sensor. Here RED is the center tap

To determine how to hook up your single strain gauge load cells to the combinator, measure the resistance with a multimeter between the three wires. You should find a larger resistance (close to double) between a pair. In our example, the resistance between red and black was 1.6 kΩ (i.e. 1600Ω), and the resistance between

white/green and red was 800 Ω. Therefore, the center tap to the strain gauge is the white/green wire. The center tap or center pin of your strain gauge connects to the "C" pin on the combinator. The larger resistance wires (red and black in this example) connect to the "+" and "-" pins on the combinator.

The combinator board hooks up the four load sensors in such a way that two resistors in the wheatstone bridge configuration are constant values and the other two are variable in this way:

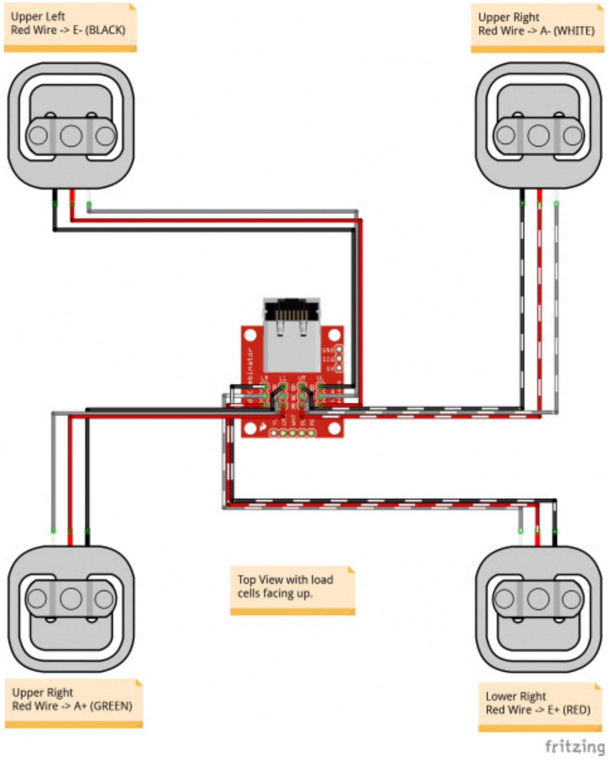

Once you have the combinator board successfully soldered to the twelve wires, you can now connect it to the HX711 amplifier board via the 4 standard load cell wires. Connecting the yellow pin is optional. You can use short jumper wires or if your electronics are a long distance away from your scale consider using an RJ45 connector and an ethernet cable to connect the combinator to the HX711 amplifier.

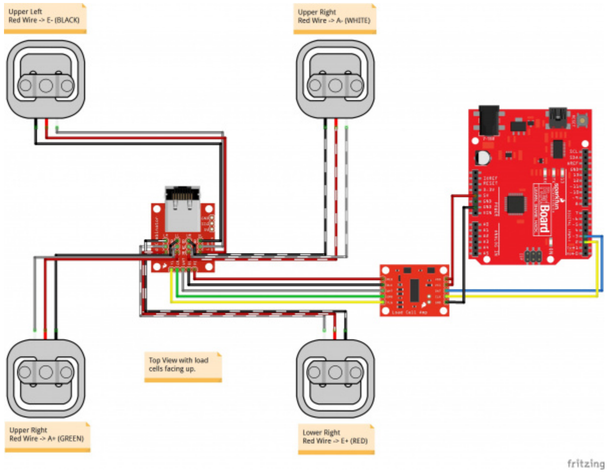

When finished, your setup should look similar to the image below. Make sure to secure the wires before placing it in an enclosure.

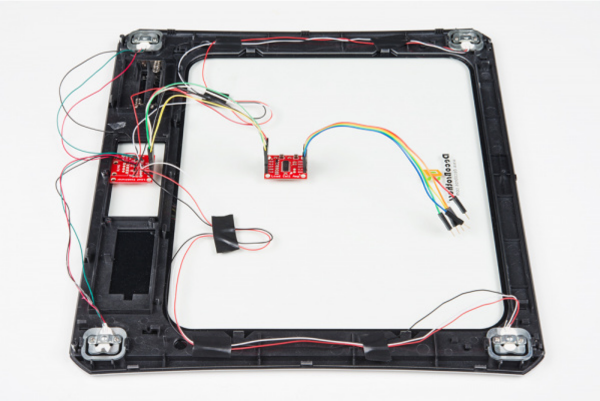

To hook up the combinator board to the HX711 match the RED, BLK, WHT, and GRN pins

Installing the HX711 Arduino Library and Examples

> **Note:** This example assumes you are using the latest version of the Arduino IDE on your desktop. If this is your first time using Arduino, please review our tutorial on installing the Arduino IDE. If you have not previously installed an Arduino library, please check out our installation guide.

Arduino Library

Now that you have your load cell, amplifier, and microcontroller hooked up, you can add your code and start calibrating your setup. You will need to download the excellent HX711 library from user **bogde**. As of the writing of this tutorial, the library has been tested on ATmega328P, ESP8266, ESP32, and STM32. Open the Arduino IDE's library manager and search for "**HX711 Arduino Library**". Click on the library to install. You can also download the ***.ZIP** file from the GitHub repo to manually install the library.

HX711 ARDUINO LIBRARY (ZIP)

Examples

You will also need to download the most up-to-date example code from the GitHub repository to follow along.

SPARKFUN HX711 LOAD CELL AMPLIFIER EXAMPLES (ZIP)

Arduino Example

The first thing you will want to work with is the calibration code "*SparkFun_HX711_Calibration.ino*" from the SparkFun examples.

```
/*
 Example using the SparkFun HX711 breakout board with a scale
 By: Nathan Seidle
 SparkFun Electronics
 Date: November 19th, 2014
 License: This code is public domain but you buy me a beer if you use this and we mee
t someday (Beerware license).

 This is the calibration sketch. Use it to determine the calibration_factor that the
main example uses. It also
 outputs the zero_factor useful for projects that have a permanent mass on the scale
in between power cycles.

 Setup your scale and start the sketch WITHOUT a weight on the scale
 Once readings are displayed place the weight on the scale
 Press +/- or a/z to adjust the calibration_factor until the output readings match th
e known weight
 Use this calibration_factor on the example sketch

 This example assumes pounds (lbs). If you prefer kilograms, change the Serial.print
(" lbs"); line to kg. The
 calibration factor will be significantly different but it will be linearly related t
o lbs (1 lbs = 0.453592 kg).

 Your calibration factor may be very positive or very negative. It all depends on the
setup of your scale system
 and the direction the sensors deflect from zero state
 This example code uses bogde's excellent library: https://github.com/bogde/HX711
 bogde's library is released under a GNU GENERAL PUBLIC LICENSE
 Arduino pin 2 -> HX711 CLK
 3 -> DOUT
 5V -> VCC
 GND -> GND

 Most any pin on the Arduino Uno will be compatible with DOUT/CLK.

 The HX711 board can be powered from 2.7V to 5V so the Arduino 5V power should be fin
e.

*/

#include "HX711.h"

#define DOUT  3
#define CLK  2

HX711 scale;

float calibration_factor = -7050; //-7050 worked for my 440lb max scale setup

void setup() {
```

```
  Serial.begin(9600);
  Serial.println("HX711 calibration sketch");
  Serial.println("Remove all weight from scale");
  Serial.println("After readings begin, place known weight on scale");
  Serial.println("Press + or a to increase calibration factor");
  Serial.println("Press - or z to decrease calibration factor");

  scale.begin(DOUT, CLK);
  scale.set_scale();
  scale.tare(); //Reset the scale to 0

  long zero_factor = scale.read_average(); //Get a baseline reading
  Serial.print("Zero factor: "); //This can be used to remove the need to tare the sc
ale. Useful in permanent scale projects.
  Serial.println(zero_factor);
}

void loop() {

  scale.set_scale(calibration_factor); //Adjust to this calibration factor

  Serial.print("Reading: ");
  Serial.print(scale.get_units(), 1);
  Serial.print(" lbs"); //Change this to kg and re-adjust the calibration factor if y
ou follow SI units like a sane person
  Serial.print(" calibration_factor: ");
  Serial.print(calibration_factor);
  Serial.println();

  if(Serial.available())
  {
    char temp = Serial.read();
    if(temp == '+' || temp == 'a')
      calibration_factor += 10;
    else if(temp == '-' || temp == 'z')
      calibration_factor -= 10;
  }
}
```

Once you have calculated your calibration factor of your load cell set up, you can move on to other code, such as the simple scale output example code, "*SparkFun_HX711_Example.ino*":

```
/*
 Example using the SparkFun HX711 breakout board with a scale
 By: Nathan Seidle
 SparkFun Electronics
 Date: November 19th, 2014
 License: This code is public domain but you buy me a beer if you use this and we mee
t someday (Beerware license).

 This example demonstrates basic scale output. See the calibration sketch to get the
calibration_factor for your
 specific load cell setup.

 This example code uses bogde's excellent library: https://github.com/bogde/HX711
 bogde's library is released under a GNU GENERAL PUBLIC LICENSE

 The HX711 does one thing well: read load cells. The breakout board is compatible wit
h any wheat-stone bridge
 based load cell which should allow a user to measure everything from a few grams to
tens of tons.
 Arduino pin 2 -> HX711 CLK
 3 -> DAT
 5V -> VCC
 GND -> GND

 The HX711 board can be powered from 2.7V to 5V so the Arduino 5V power should be fin
e.

*/

#include "HX711.h"

#define calibration_factor -7050.0 //This value is obtained using the SparkFun_HX711_
Calibration sketch

#define DOUT  3
#define CLK  2

HX711 scale;

void setup() {
  Serial.begin(9600);
  Serial.println("HX711 scale demo");

  scale.begin(DOUT, CLK);
  scale.set_scale(calibration_factor); //This value is obtained by using the SparkFun
_HX711_Calibration sketch
  scale.tare(); //Assuming there is no weight on the scale at start up, reset the sca
le to 0

  Serial.println("Readings:");
}
```

```
void loop() {
  Serial.print("Reading: ");
  Serial.print(scale.get_units(), 1); //scale.get_units() returns a float
  Serial.print(" lbs"); //You can change this to kg but you'll need to refactor the c
alibration_factor
  Serial.println();
}
```

Check out the other example codes provided in the GitHub repo for powering down the HX711 and known zero startup).

Resources and Going Further

Now that you've successfully got your HX711 up and running, it's time to incorporate it into your own project!

For more information, check out the resources below:

- Schematic (PDF)
- Eagle Files (ZIP)
- HX711 Datasheet (PDF)
- GitHub
    - Arduino Library
    - Design Files & Example Code
- SFE Product Showcase: Dog Treat Dispenser
- SparkFun Fellowship of the Things #2 - Weight for Treats
    - Particle Photon Example Code

Need some inspiration for your next project? Check out some of these related tutorials:

OpenScale Applications and Hookup Guide

OpenScale allows you to have a permanent scale for industrial and biological applications. Learn how to use the OpenScale board to read and configure load cells.

IoT Industrial Scale

What does a baby elephant weigh? How much impact force does a jump have? Answer these questions and more by building your very own IoT industrial scale using the SparkFun OpenScale.

IoT Weight Logging Scale

This tutorial will show you how to make a scale that logs your weight to a custom website on the Internet. The principles can be extrapolated to any type of data.

Qwiic Scale Hookup Guide

Create your own digital scale quickly and easily using the Qwiic Scale!

Need even more? Check out these additional links below:

- SparkFun IoT Beehive
  - The Internet of Bees: Adding Sensors to Monitor Hive Health
  - GitHub Repo: Open Scale
- Phidgets: Load Cell Primer - Article about wheatstone bridges and load cell types
