# Supplementary material for: A low-cost, open-source device to evaluate limb stiffness in a rabbit model of cerebral palsy
Source: Front Bioeng Biotechnol. 2025 Jun 5;13:1554775. doi: 10.3389/fbioe.2025.1554775 (PMC12177462; doi:10.3389/fbioe.2025.1554775)
Supplement: Supplementary file 2 [file DataSheet1.zip › MarinManuel-TorqueMeter-772995c/Assets/Datasheets/RP3502_Series.pdf]

SERIES RP 3502 SWITCHES

PUSHBUTTON SWITCHES - PANEL MOUNT

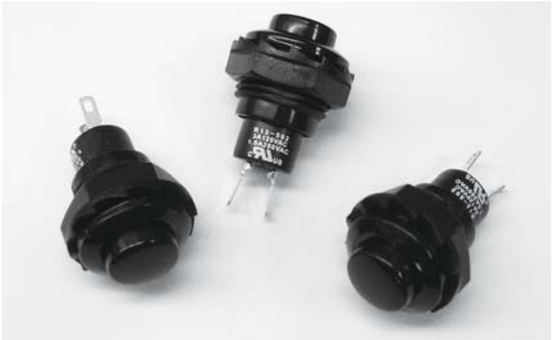

SPECIFICATIONS

Contact Rating:	3A @ 120 VAC<br>1.5A @ 250 VAC
Life Expectancy (Mechanical):	6,000 cycles
Life Expectancy (Electrical):	6,000 cycles
Contact Resistance:	50mΩ (initial max.)
Insulation Resistance:	500MΩ min.
Dielectric Strength:	1,000 VAC 1 minute
Operating Temperature:	-20°C to +65°C

MATERIALS

Plastic:	Nylon (UL flame: 94V-2)
Metal:	Nickel plated brass

FEATURES & BENEFITS

- Single Pole, Single throw with momentary function
- Rugged, Panel mount design
- Available in Nylon or metal frame body

APPLICATIONS/MARKETS

- Test & Instrumentation
- Industrial controls
- Telecommunications
- Networking

HOW TO ORDER

SERIES	MODEL NO.	BODY STYLE	FUNCTION	BUTTON COLOR
RP	3502	None = Plastic<br>M = Metal	A = OFF-(ON)<br>B = ON-(OFF)	BLK = Red<br>RED = Red<br>GRN = Green<br>YEL = Yellow

Example Ordering Number

RP–3502–M–A–BLK

Specifications subject to change without notice.

RP 3502

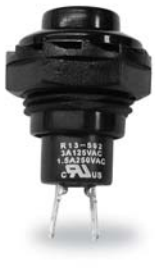

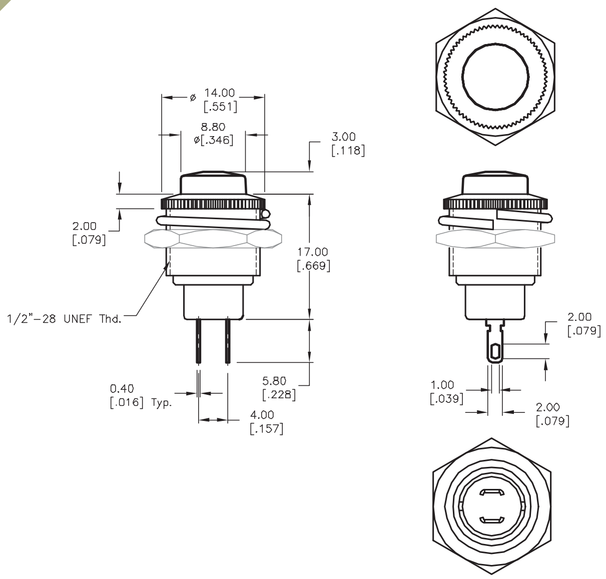

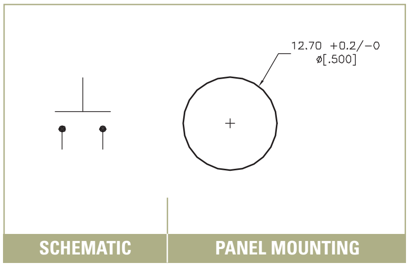

E•SWITCH®
Phone: 800-867-2717
Fax: 763-531-8235
