# Supplementary material for: A low-cost, open-source device to evaluate limb stiffness in a rabbit model of cerebral palsy
Source: Front Bioeng Biotechnol. 2025 Jun 5;13:1554775. doi: 10.3389/fbioe.2025.1554775 (PMC12177462; doi:10.3389/fbioe.2025.1554775)
Supplement: Supplementary file 2 [file DataSheet1.zip › MarinManuel-TorqueMeter-772995c/Assets/Datasheets/TAL221.pdf]

WWW.HTC-SENSOR.COM

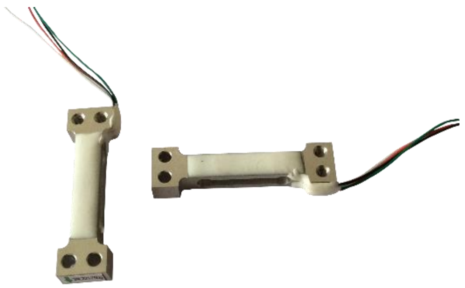

Features:

◆Capacity： 100~1500g
◆Material: aluminum-alloy
◆Type: Parallel beam type
◆Defend grade: IP65
◆Application : body scales, hand scales, kitchen scales, postal scales, fishing scales, baby body scales and other micro electronic weighing systems.

Electrical connection and Dimensions:(dimension unit: mm)

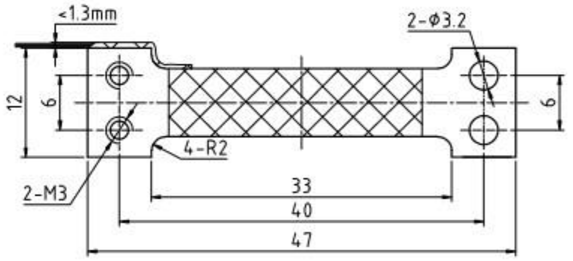

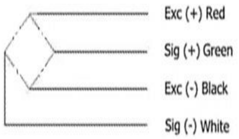

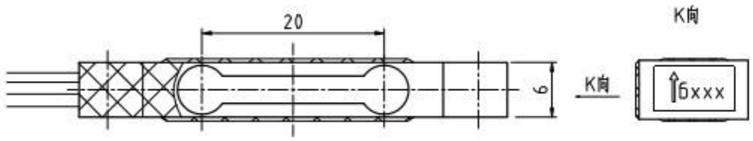

Specifications:				
capacity	g	100, 150, 200	300,500,750	1000,1500
rated output	mV/V	0.6 ± 0.15	0.7 ± 0.15	1.0 ± 0.15
safe overload	%FS	150		
ultimate overload	%FS	200		
excitation voltage	Vdc	≤6		
combined error	%FS	0.05		
zero balance	%FS	± 0.1		
non-linearity	%FS	± 0.05		
hysteresis	%FS	± 0.05		
repeatability	%FS	± 0.05		
creep	%FS/3min	± 0.05		
input resistance	Ω	1090 ± 10		
output resistance	Ω	1000 ± 10		
insulation resistance	M Ω	≥ 2000 @ 50 Vdc		
operating temperature range	℃	-10 ~ +40		
compensated temperature range	℃	-20 ~ +60		
temperature coefficient of SPAN	%FS/10℃	± 0.1		
temperature coefficient of ZERO	%FS/10℃	± 0.1		
Corner correction	%FS	± 0.1		
Electrical connection	cable	4 color wire, Ø0.6 × 110 mm<br>excitation(+):Red excitation(-):Black<br>signal(+):Green signal(-):White		

※**Ordering code:** model-capacity- rated output-accuracy-defend grade- the length of cable
