# Supplementary material for: A low-cost, open-source device to evaluate limb stiffness in a rabbit model of cerebral palsy
Source: Front Bioeng Biotechnol. 2025 Jun 5;13:1554775. doi: 10.3389/fbioe.2025.1554775 (PMC12177462; doi:10.3389/fbioe.2025.1554775)

SERVO MOTOR SG90 DATA SHEET

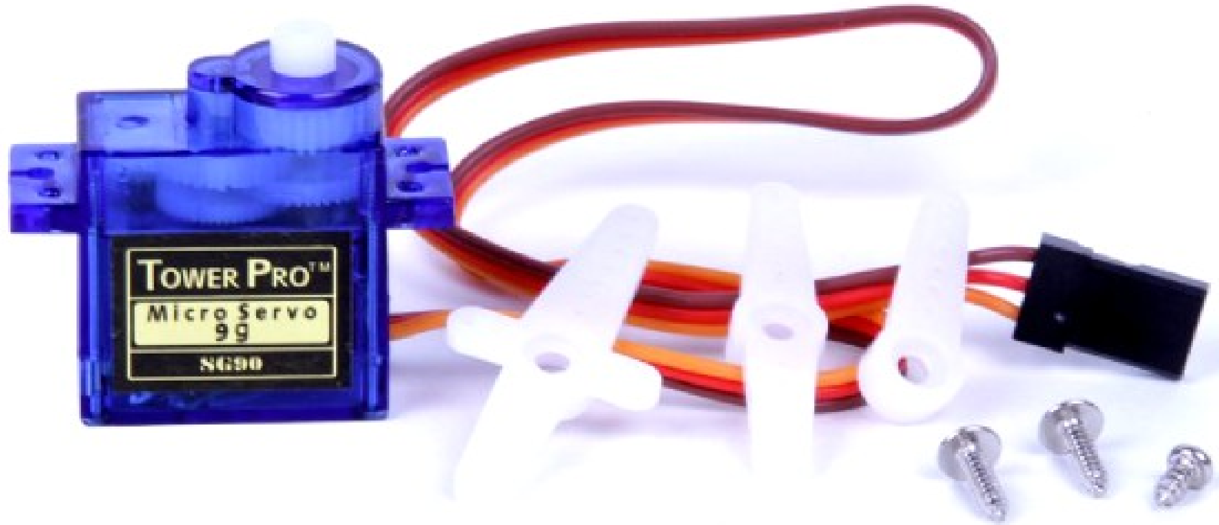

Tiny and lightweight with high output power. Servo can rotate approximately 180 degrees (90 in each direction), and works just like the standard kinds but smaller. You can use any servo code, hardware or library to control these servos. Good for beginners who want to make stuff move without building a motor controller with feedback & gear box, especially since it will fit in small places. It comes with a 3 horns (arms) and hardware.

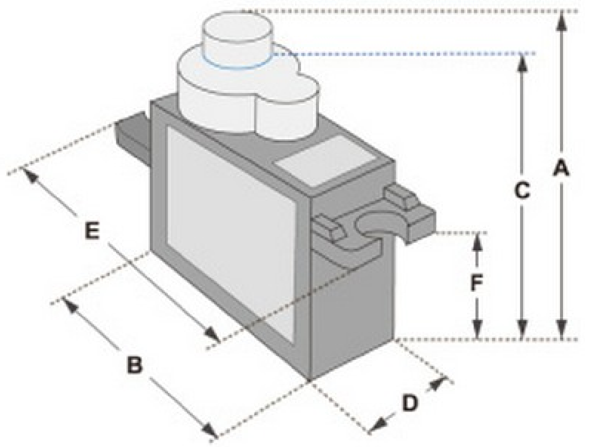

Dimensions & Specifications
A (mm) : 32
B (mm) : 23
C (mm) : 28.5
D (mm) : 12
E (mm) : 32
F (mm) : 19.5
Speed (sec) : 0.1
Torque (kg-cm) : 2.5
Weight (g) : 14.7
Voltage : 4.8 - 6

Position "0" (1.5 ms pulse) is middle, "90" (~2ms pulse) is middle,
is all the way to the right, "-90" (~1ms pulse) is all the way to the left.

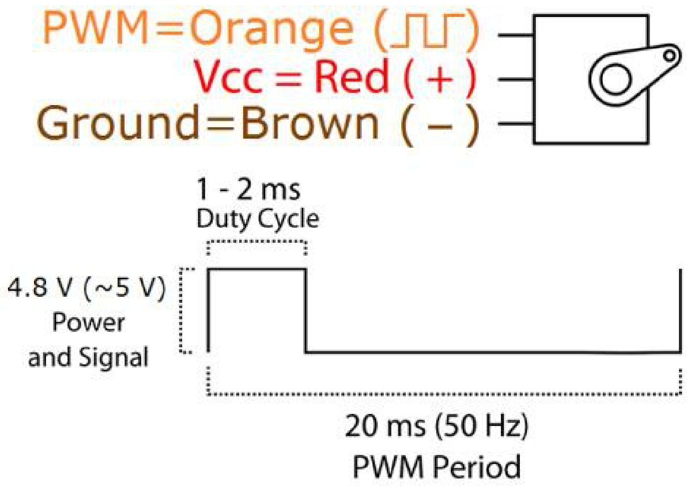

Supplement: Supplementary file 2 [file DataSheet1.zip › MarinManuel-TorqueMeter-772995c/Assets/Datasheets/sg90_datasheet.pdf]
