# Supplementary figures and images for: A low-cost, open-source device to evaluate limb stiffness in a rabbit model of cerebral palsy
Source: Front Bioeng Biotechnol. 2025 Jun 5;13:1554775. doi: 10.3389/fbioe.2025.1554775 (PMC12177462; doi:10.3389/fbioe.2025.1554775)

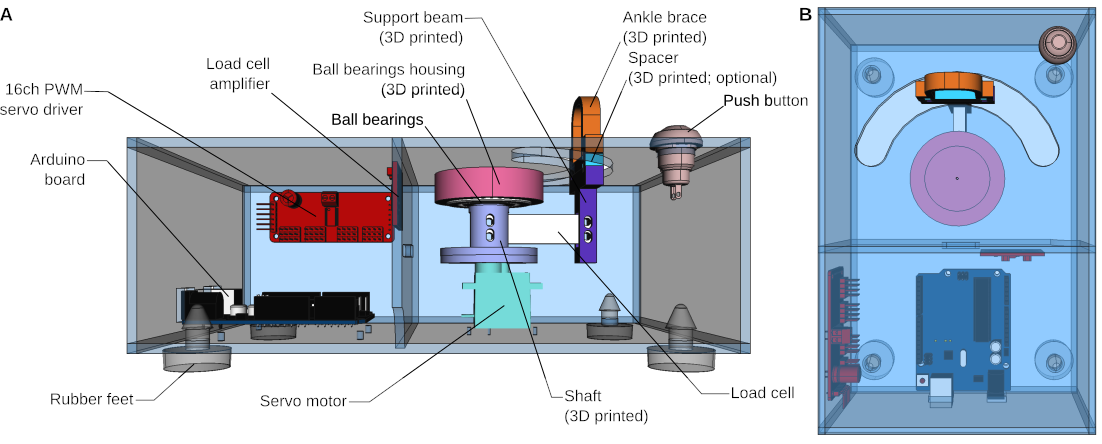

Supplement: Supplementary file 2 [file DataSheet1.zip › MarinManuel-TorqueMeter-772995c/Assets/Figure 1.png]
